# Structure of Pb(Fe_2/3_W_1/3_)O_3_ single crystals with partial cation order

**DOI:** 10.1038/s41598-020-71438-4

**Published:** 2020-09-03

**Authors:** S. A. Ivanov, A. I. Stash, L. Riekehr, Y.-S. Chen, Z.-G. Ye

**Affiliations:** 1 Department of Chemistry, M.V. Lomonosov Moscow State University, Leninskie Gory 1/3, Moscow, Russia 119991; 2 Department of Engineering Sciences, Solid State Physics, Angstrom Laboratory, Uppsala University, Box 534, 751 21 Uppsala, Sweden; 3 A. N. Nesmeyanov Institute of Organoelement Compounds of Russian Academy of Science, Vavilov St., 28, Moscow, Russia 119991; 4 Department of Engineering Sciences, Solid State Electronics, Angstrom Laboratory, Uppsala University, Box 534, 751 21 Uppsala, Sweden; 5 NSF’s ChemMatCARS Beamline@APS, The University of Chicago, Argonne, IL 60439 USA; 6 Department of Chemistry and 4D LABS, Simon Fraser University, 8888 University Drive, Burnaby, BC V5A 1S6 Canada

**Keywords:** Materials science, Condensed-matter physics, Ferroelectrics and multiferroics

## Abstract

Despite intensive studies on the complex perovskite Pb(Fe_2/3_W_1/3_)O_3_ (PFWO) relaxor, understanding the exact nature of its multifunctional properties has remained a challenge for decades. In this work we report a comprehensive structural study of the PFWO single crystals using a combination of synchrotron X-ray diffraction and high-resolution electron microscopy. The set of {h + ½, k + ½, l + ½} superlattice reflections was observed for the first time based on single-crystal synchrotron X-ray experiments (100–450 K) and transmission electron microscopy investigations, which indicates some kind of B-cation ordering in PFWO which had been thought to be totally disordered. It was found that (1) the crystal structure of PFWO should be described by a partly ordered cubic perovskite (i.e. *Fm* − *3m*), (2) the weak ferromagnetic properties and excess magnetic moment of PFWO can be understood based on non-random distribution of Fe cations between the *4a* and *4b* sites, and (3) the Pb displacement disorder is present in this material and the cations are probably displaced along the <100> directions. The X-ray diffraction results of this investigation show that partial cation ordering indeed exists in PFWO, which makes it necessary to revisit the generally accepted interpretations of the results obtained up to date. In agreement with X-ray diffraction study the main results of TEM study include: (1) a long range order that can be described with the *Fm* − *3m* symmetry is reliably detected, (2) the coherence length of that long range order is in the order of 1–2 nm and (3) no remarkable chemical inhomogeneity is found in the tested PFWO crystal, excluding the possibility of a compositional ordering arising from substitutional defects in the perovskite structure.

## Introduction

Pb(Fe_2/3_W_1/3_)O_3_ (PFWO) belongs to the family of Pb-based multiferroic relaxor ferroelectric complex perovskites (${\text{AB}}_{{{1} - {\text{x}}}}^{\prime } {\text{B}}_{{\text{x}}}^{\prime \prime } {\text{O}}_{{3}}$)^1–3^. PFWO is formed from simple perovskite ABO_3_ with the magnetic ions Fe^3+^ (3d^5^, S = 5/2) and the non-magnetic ions W^6+^ (5d^0^, S = 0) sharing the B-site of the perovskite-type structure. It is generally accepted that this material exhibits a disordered perovskite structure, where Fe^3+^ and W^6+^ ions are randomly distributed at the centers of the BO_6_ octahedra^4–6^. PFWO has been the subject of numerous studies due to its attractive combination of spin and dipole orderings^7–10^. An interesting feature of PFWO is associated with the presence of magnetic ions Fe^3+^ with a relatively high occupancy of 2/3 on the B-octahedral sites, leading to one of highest magnetic ordering temperatures established in multiferroics materials^6^, with the para- to antiferromagnetic transition occurring around T_N_ = 340–380 K^11–14^, where the magnetic ordering is due to the superexchange interaction between the Fe ions through the O ions. Also, this material is a relaxor ferroelectric with a broad and frequency-dependent dielectric maximum (also called diffuse phase transition) around T_max_ (or T_C_) = 150–200 K^15,16^.

Despite the 60-year history of PFWO^17,18^, it is still impossible to say with confidence that its physico-chemical properties have been fully understood. For instance, the question of the existence of a certain degree of cation order was raised in the first study of this material^17–19^, but it remains an open question. Initial idea was to order the ions and create in the crystal two sublattices corresponding to formula Pb[Fe]_0*.*5_[Fe_1*/*3_W_2*/*3_]_0*.*5_O_3_. Then the magnetic moments of the two sublattices directed oppositely to each other would not be equal. As a result, the sum moment of the magnetization would not be equal to zero, and the crystal would be ferrimagnetic^19^. On the basis of the experimental results obtained by that time, it became quite obvious that the method of preparation of PFWO samples plays a decisive role and, under optimal synthetic conditions, a certain degree of order was experimentally discovered^20–22^. The problem was redoubled by the fact that the preparation of single-phase ceramic samples of the required stoichiometry was a rather difficult task due to the formation of several stable impurity phases in the system accompanying this compound. The resulting impurity phases did not help to resolve the problem of order–disorder, making it all a more urgent task of obtain perfectly pure perovskite single crystals. A number of attempts to grow single crystals have been made^2–4,17,18^, but only those reported in Refs^23,24^ can be considered successful. A comprehensive study of the physico-chemical properties of the PFWO only confirmed the disordered arrangement of Fe and W cations in the perovskite B sublattice^25–27^.

It was observed also that the ordering degree in PFWO is hardly modified by annealing treatments, but is very sensitive to doping^28–36^. Investigations of the effects of different additives on the order in the PFWO-based ceramics demonstrated that relatively small alterations in the chemistry can produce very large changes in the thermodynamic stability of the ordered structure and induce significant modifications in the magnetic behavior and dielectric/ferroelectric properties, for example, the influences of the Na, Mg, Ti, Zr, Sc, and Yb dopings^37–48^. Small amounts of Na, Sc and Yb cation substitutions promote a transformation to an ordered cubic (*Fm* − 3*m*) structure. Specific mechanisms of the influence of the compositional order–disorder on the relaxor/ferroelectric phase transition in PFWO are still a topic of intensive discussion. It is reported that quenching from a high temperature can eliminate the B site ordering in PFWO and a long time annealing at a relatively low temperature can recover the B site ordering^13,34^. The order–disorder transformation in PFWO could be triggered also by mechanical activation^49^. The fact that a rather small concentration (only 5 at.%) of dopants is sufficient to induce for disorder–order structural transition only adds more intrigue to the problem and makes the study of structural order in PFWO a more interesting subject.

It is important to note that the structural properties of the PFWO single crystals were studied quite insufficiently with lack of reliable data^14,23,24^. Crystallochemical studies of the cubic phase of perovskites traditionally have largely been neglected. This is owing to the presumption that the structure has all atoms at their special positions fixed by the crystallographic symmetry, and so there are only few structural parameters to vary. Accordingly, little attention has been paid to the details of the cubic phase and especially the degree of order–disorder. The published structural data about the crystal structure parameters of the title compound are scarce and confusing. The tolerance factor t value (see definition in ^1^) was used as a guide to predict the cubic crystal symmetry of PFWO (t = 1.059), though its usefulness is limited because it is based on a purely geometrical consideration. At the same time the crystal structural solution of Pb-based perovskites is often very challenging, even using the well established methodology of single-crystal X-ray crystallography. This task becomes even more difficult due to possible order–disorder effects, large absorption, strong extinction and weak oxygen contribution. Despite the interest in the physical properties of PFWO, several features of the underlying crystal structure still remain unresolved. For example, two competing space groups, *Pm* − *3m*^14,23,24^ and *Fm* − *3m* (current research), have been proposed for the crystal structure. Apart from the above-mentioned studies, almost no other detailed structural investigations have been carried out on the cubic phase of PFWO, presumably because one would not normally expect anything new and interesting.

Because the relaxor and magnetic properties of PFWO are critically dependent on the nature of octahedral order–disorder, a proper appreciation of the structural details of the cubic phase is paramount for the understanding of structure–property relationships. In this work, we demonstrate that a combination of two modern techniques, synchrotron X-ray crystallography and electron microscopy, is a viable solution to resolve the problem of cation ordering in PFWO.

In order to avoid any contradicting results caused by sample inhomogeneity, PFWO single crystals were used in this study and the powder samples were obtained from the crystals.

We find with great interest that the suspected cation ordering indeed exists in the PFWO crystals and the ordered *Fm* − *3m* structure persists in temperature range of 100–450 K. Our finding of cation ordering in PFWO makes it necessary to revisit the existing view of the structural order–disorder in this material and the interpretation of its relaxor and multiferroic properties.

## Results

### Refinement of XRD data

As a first approach, two simple models were proposed for the structure of PFWO: a complete disordered structure (cubic symmetry, e.g. *Pm* − *3m*) with a random distribution of cations over the octahedral sites, and a complete ordered superstructure (e.g. *Fm* − *3m*).

During the first stage of structural investigation X-ray powder diffraction (XRPD) pattern of phase pure PFWO powder prepared from single crystals at 295 K was indexed using the space group *Pm* − *3m* with a unit-cell parameter similar to the ideal cubic perovskite aristotype, a_0_ = 3.979(1) Å. Any possibility of long-range order between the two cations occupying the B-site positions was excluded because there are no additional superstructure reflections on XRPD pattern.

However, a series of high-resolution XRD experiments carried out on the PFWO single crystals shows that the cubic phase is more complicated than thought hitherto. The main aim of this research was to determine the crystallographic parameters of crystals such as lattice constants, atomic positions and degree of cationic order. Fortunately, the X-ray scattering lengths for Fe and W are strongly different with an attractive level of contrast.

**Figure 1:**
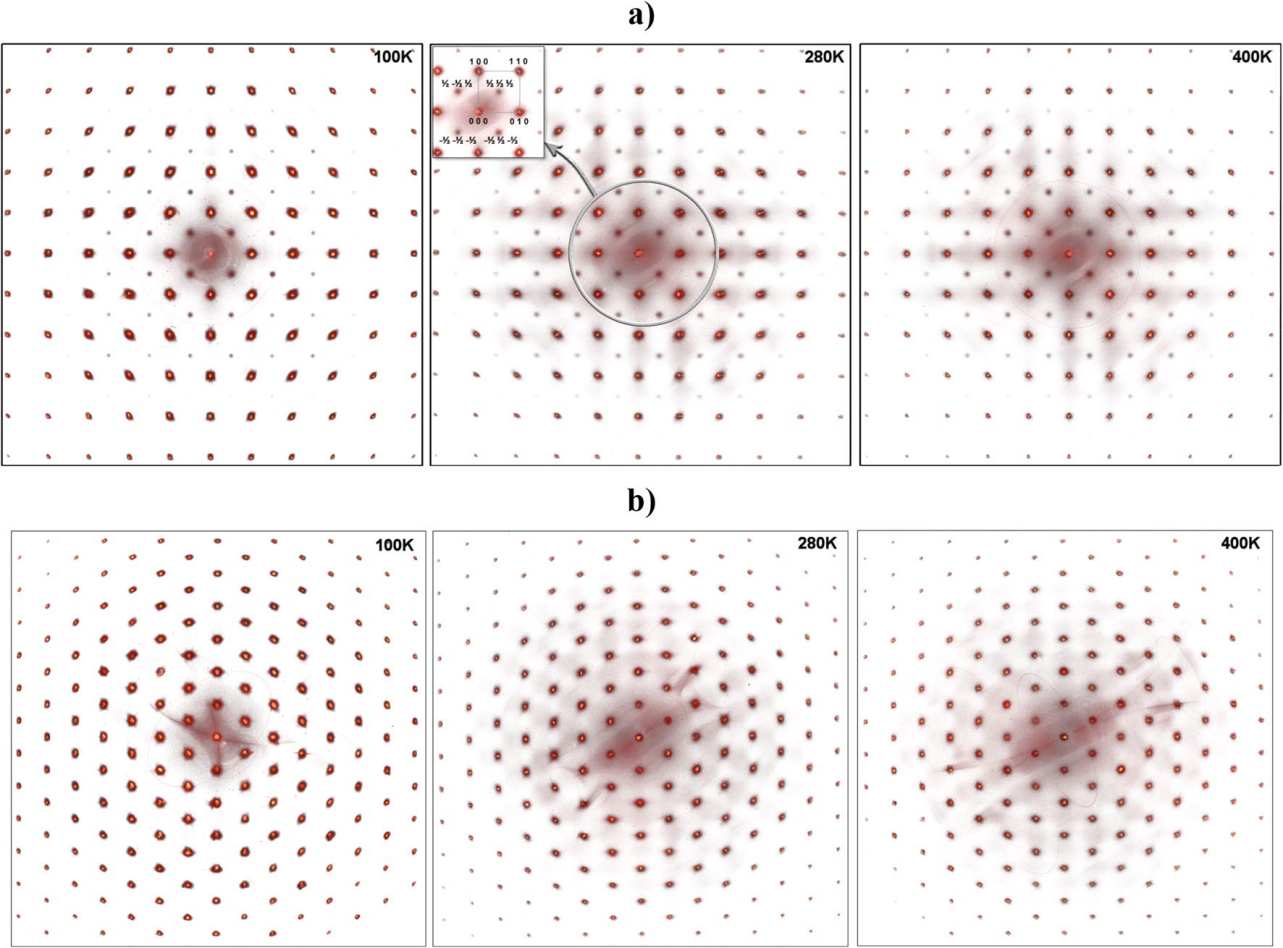
Selected-area diffraction patterns of PFWO taken along the [001] (**a**) and [111] (**b**) directions at different temperatures. Note the presence of the weak superstructural reflections of (½, ½, ½)-type which can be seen at all the investigated temperatures. Strong reflections indicate a dominant 3.97 Å primitive cubic subcell. The figures were obtained by converting each pixel of the frames after subtracting the background into points of reciprocal space using the CMC3dsi program [https://chemmatcars.uchicago.edu/software/].

**Figure 2:**
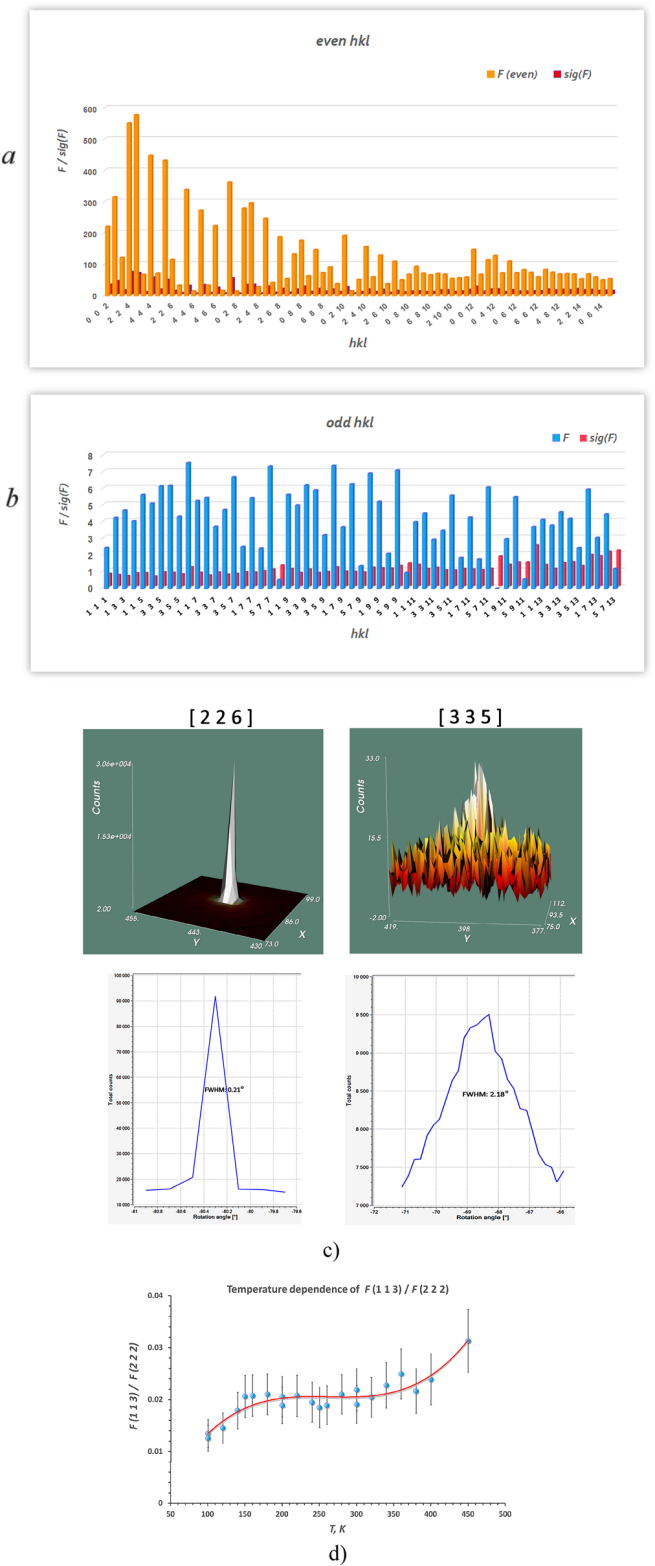
Histogram of the observed main (**a**) and superstructural (**b**) reflections for PFWO; (**c**) 3D view and rocking curves for the (226) and (335) reflections. (**d**) Temperature evolution of the ratio of structural amplitude for the (113) and (222) reflections.

The set of the X-ray diffraction frames of PFWO obtained at different temperatures is shown in Fig. 1. The pattern contained reflections from a cubic perovskite structure together with quite strong and sharp additional half-integer superstructure peaks, i.e. (h + ½, k + ½, l + ½), which is characteristic of an F-type doubled lattice, indicating possible long-range Fe/W ordering between the two different cation sites (4a and 4b). Two types of observed reflections for PFWO are shown on Fig. 2a,b. On Fig. 2c, a 3D view and rocking curves for the main (226) and superstructural (335) reflections are presented. It has been found (see Fig. 2d) that the superstructural reflections remain practically unchanged with the change of temperature in terms of both intensity and peak shape.

**Figure 3:**
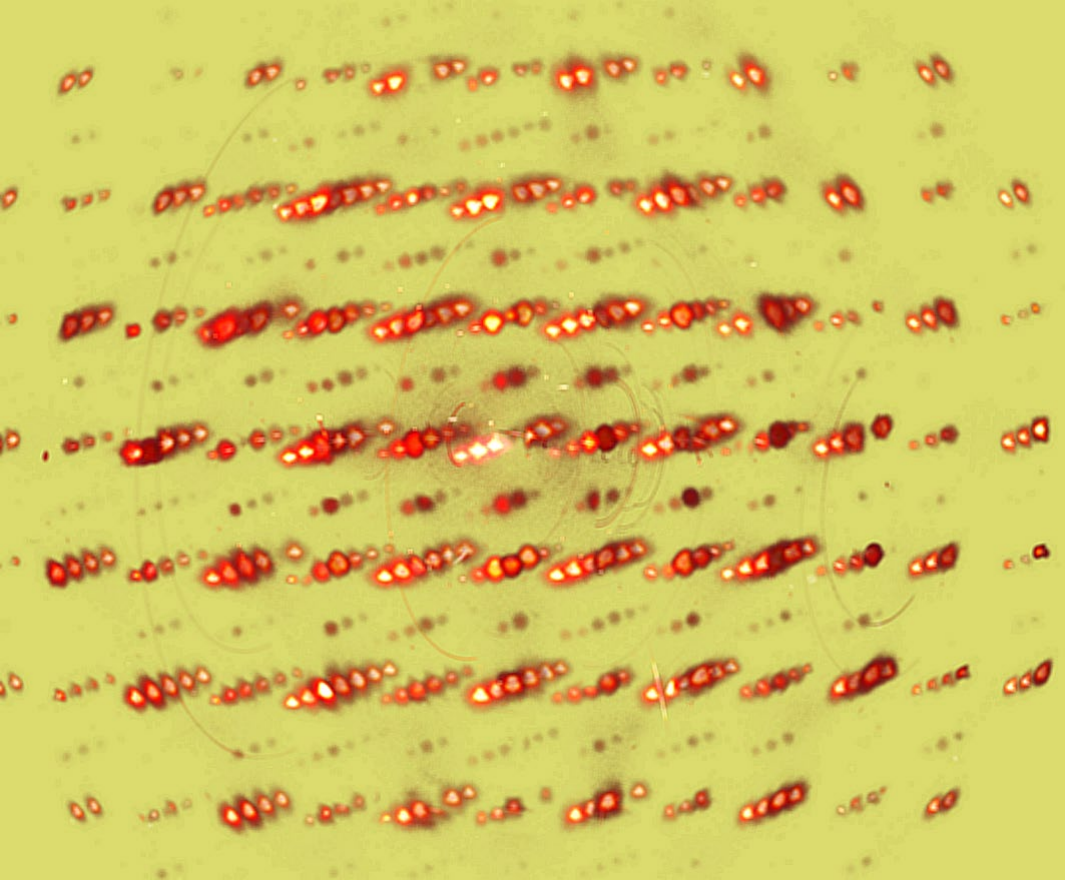
Set of the main and superstructural reflections in reciprocal space for PFWO.

The position of reflections in reciprocal space are presented on Fig. 3. On the first stage of structural analysis of PFWO a disordered model (i.e. *Pm* − *3m*) without superstructural reflections was tested. Refinement of disordered B-site cation structure gave us *R*-value = 4.9%.

In the cubic *Fm* − *3m* phase of the PFWO crystal all the atoms except oxygen are in special positions. Thus, from the structural parameters, the coordinate of the oxygen anion (shift along *B'–O–B"* bond), the isotropic thermal parameters for all the atoms, and the occupancy positions for the Pb, Fe and W cations were refined.

Taking advantage of the larger contrast existing between X-ray scattering factors of Fe and W, the determination of the anti-site effect was effectively performed from high-resolution XRD. From the EDS analysis results and preliminary refinements, it was found that the *4a* and *4b* site populations are equal to 1 within two standard deviations. The initial values of occupation factors for the *4a* and *4b* positions were selected as 2/3 and 1/3 for Fe and W, respectively.

The final refinement was carried out using the linear constraints on the occupation of these positions, so that we obtained the refined crystallographic formula of this compound to be Pb_2_[Fe_0.69_W_0.31_]_4a_[Fe_0.64_W_0.36_]_4b_O_6_.

The extent of the B site cation order remained effectively unchanged in the whole tested temperature range. So, as a good approximation, we can assume that this compound presents a partial B-site cation order and the chemical contents of the *4a* and *4b* positions are not equivalent.

**Figure 4:**
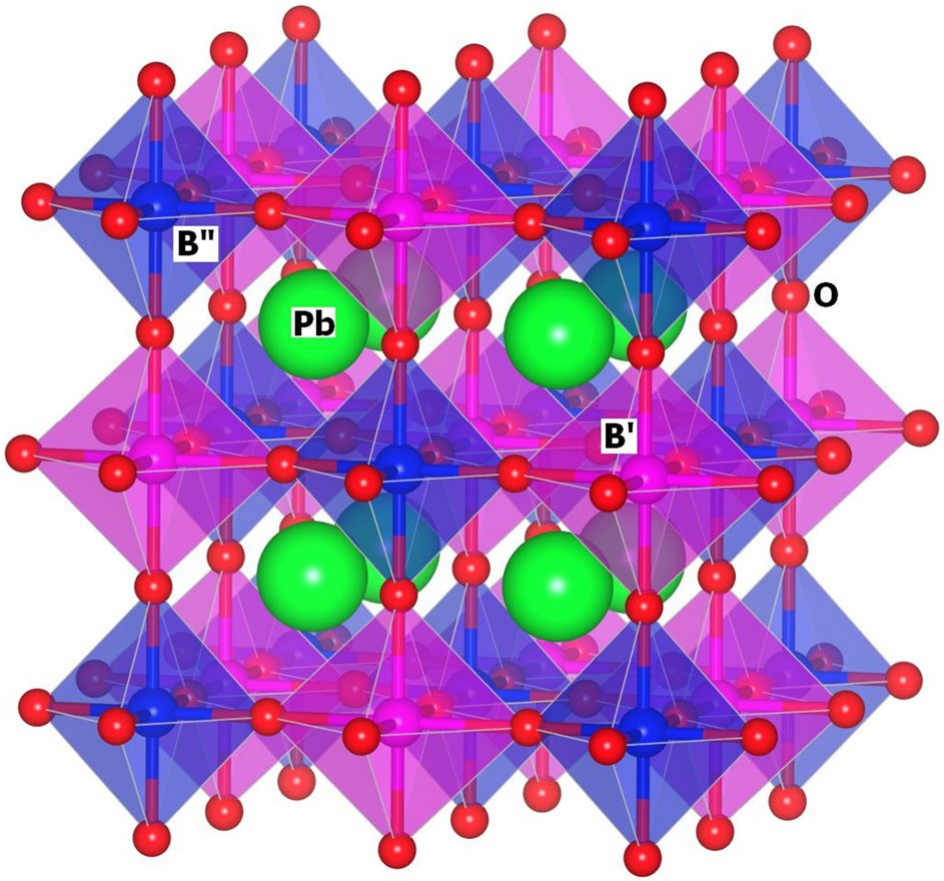
Polyhedral representation of the ordered structure of PFWO crystal.

The ordering degree *S* can be defined as *S* = *S*_(*4a*)_ − *S*_(*4b*)_, where *S*_(*4a*)_ *and S*_(*4b*)_ denote the fractional site occupancies of the respective crystallographic sites. The results of the structural refinement for the PFWO cubic phase are given in Tables 1 and 2. All the octahedral sites of the PFWO structure are filled by cations B' and B", alternating along the axes of the cube. The octahedra B'O_6_ and B"O_6_ have different sizes due to small shift of the O anion towards B" of higher-valence cation (see Fig. 4). More detailed structural data collected at 100, 280 and 400 K are provided in Supplementary Materials in .CIF format.

**Table 1:** Summary of structural refinement results of PFWO sample at different temperatures using synchrotron X-ray single crystal diffraction data.

T, K		100	280	400
a, Å		7.9537(4)	7.9582(4)	7.9686(4)
V, Å^3^		503.16(8)	504.02(8)	505.99(9)
Pb	x	0.2201(2)	0.2198(2)	0.2207(2)
	y	1/4		
	z	¼		
	Uani	0.0206(6)	0.0184(5)	0.0217(6)
B’	x	0		
	y	0		
	z	0		
	Uani	0.0089(3)	0.0068(3)	0.0083(3)
	n Fe/W	0.685/0.315	0.693/0.307	0.692/0.308
B”	x	1/2		
	y	1/2		
	z	½		
	Uani	0.0082(4)	0.0066(3)	0.0079(3)
	n Fe/W	0.649/0.351	0.641/0.359	0.642/0.358
O	x	0.2502(3)	0.2502(2)	0.2502(1)
	y	0		
	z	0		
	Uani	0.0163(7)	0.0147(7)	0.0172(6)
R[F^2^ > 2σ(F^2^)]		0.0216	0.0186	0.0197
Number of reflections		121	128	129
Extinction ()		0.29(3)	0.62(5)	0.57(4)

**Table 2:** Temperature evolution of the thermal parameters of PFWO.

Cation	T, K	*U11*	*U22*	*U33*	*U23*
Pb	100	0.0134(7)	0.0242(6)	0.0242(6)	− 0.0008(1)
	280	0.0111(6)	0.0220(5)	0.0220(5)	− 0.0009(1)
	400	0.0146(6)	0.0253(5)	0.0253(5)	− 0.0008(1)
B’	100	0.0089(3)	0.0089(3)	0.0089(3)	
	280	0.0068(3)	0.0068(3)	0.0068(3)	
	400	0.0083(3)	0.0083(3)	0.0083(3)	
B”	100	0.0082(4)	0.0082(4)	0.0082(4)	
	280	0.0066(3)	0.0066(3)	0.0066(3)	
	400	0.0079(3)	0.0079(3)	0.0079(3)	
O	100	0.0089(14)	0.0200(10)	0.0200(10)	
	280	0.0078(14)	0.0175(11)	0.0175(11)	
	400	0.0084(12)	0.0215(9)	0.0215(9)

From Table 1 it is evident that the thermal parameter of Pb cation is much bigger than the thermal parameters of O and Fe/W cations, and it is similar to that of other ferroelectric materials that contain lead^50–52^ and consistent with our previous report on PFWO^13^. The unusually strong ionic displacement parameters associated with the Pb and O sites, and the low bond valence sums of these ions, provide clear evidence for the disordered ionic displacements in PFWO in the whole temperature range. Such behavior is characteristic of cubic perovskites that contain Pb^2+^ ion on the A site, which is due to the existence of a stereochemically active lone electron pair on lead ion Pb^2+^^50^. To retain the cubic symmetry, the Pb cation adopts split positions for the A-site. In contrast, the Fe and W cations and the oxygen anion were kept on their special sites since their calculated displacements could not improve the refinement results. In this way, the structure was well refined as a disordered cubic one, with the disorder being confined on the Pb site only. The disordered shifts of the lead from its special position along the cubic directions give rise to a strongly marked minimum of the R factor versus shift amplitude. This minimum is associated with a shift value of about 0.24 Å, where normal thermal parameter values are recovered. The Pb atom exhibited an anisotropic displacement ellipsoid of disc shape.

**Figure 5:**
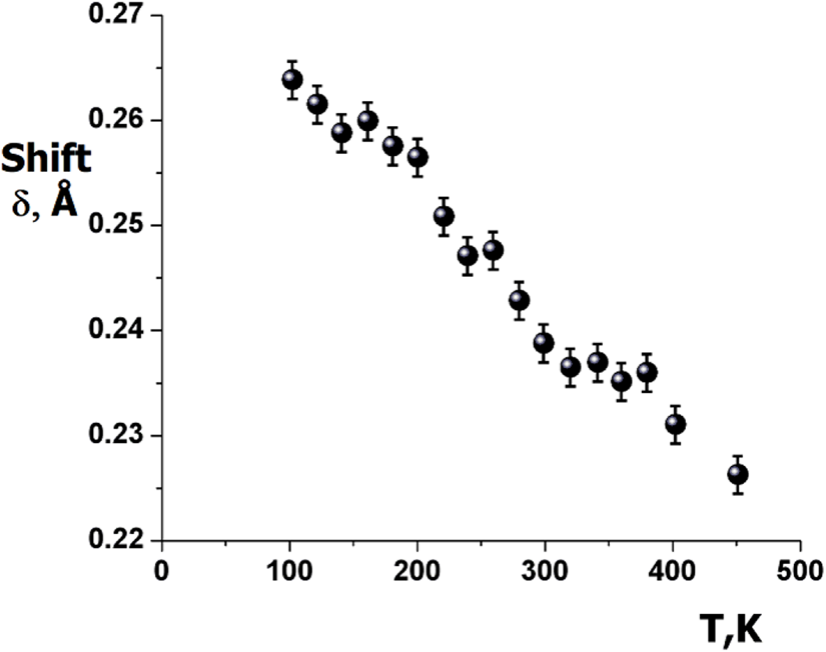
Thermal evolution of the Pb cation shift (δ) along [100].

Several attempts were performed to model these shifts by displacing the Pb^2+^ ion away from its high-symmetry site in a disordered manner. However, the results were not fully conclusive in the cases of [110] and [111] shifts (see Table 3). Nonetheless, it can be seen from the ionic displacement parameters shown in Table 2 that the Pb cations are indeed displaced locally from their ideal sites. Figure 5 shows the variation of displacement parameter as a function of temperature for the Pb cation along the [100] direction. From stereochemical considerations, the Pb cation of lone pair electrons would never be located at the origin of the cubic perovskite unit cell. It should be noted that the large Debye–Waller factor was not due to thermal vibrations, but more probably resulted from the local displacements of Pb cations away from their average lattice positions. Since these shifts are correlated over short distances only, the long-range structure remains cubic, with an increasing thermal factor.

**Table 3:** Deviation shift of Pb cation (*δ*) from the position (¼, ¼, ¼) (in Å) for different crystallographical directions.

T, K	*δ*_<*100*>_	*R*_<*100*>_	*δ*_<*110*>_	*R*_<*110*>_	*δ*_<*111*>_	*R*_<*111*>_
100	0.230(1)	0.0216	0.226(3)	0.0224	0.221(1)	0.0236
280	0.231(1)	0.0186	0.237(3)	0.0212	0.224(1)	0.0239
400	0.229(1)	0.0197	0.223(2)	0.0216	0.217(2)	0.0249

Values R-factors are also given for different models and temperatures.

The differential map for PFWO indicates primarily the [100]-disorder for the Pb ion, instead of along [111] as suggested from our earlier NPD studies on ceramic sample^13^. All other types of Pb displacements led to larger values of R_B_ .

We also studied the possibility that the O anions could be statistically disordered around their average positions by displacing them within a small distance. This indeed improved the agreement factor. However, the best fit was achieved by introducing the refined anisotropic displacement parameters for the O anions.

It is worth noting that in the perovskites there could be several possible sources of superstructure reflections: the transformation of the *Pm* − *3m* phase to a lower symmetry (tetragonal) one; the tilting of octahedra; the antiparallel displacement of cations; and the ordering of chemical species, such as dissimilar A- or B-site cations and oxygen vacancies. Having a cubic structure, we can always choose a space group with lower symmetry related with octahedral tilting. In the case of PFWO, the cubic *Fm* − *3m* space group can be easily converted into tetragonal I4/mcm, originated owing to octahedral tilting. During this conversion a new condition for structural amplitude (0kl: k,l = 2n) appears. Consequently, we can lose more than 10% of the observed experimental reflections (e.g., 277 of 2,673 at 280 K) that agree with the conditions for systematic absences. These reflections have an intensity of up to 10sigma (F). In the case of e.g. *Fm* − *3m* all the registered structural amplitudes meet the conditions for the reflections. We tried to solve the structure in other space groups related with the tilting, but they did not produce satisfactory results. Non-centrosymmetric structures are excluded from consideration because the crystals of PFWO are found to be centrosymmetrical by the method of second harmonic generation using laser radiation.

The (h + ½, k + ½, l + ½) reflections could not come from the octahedra tilts because PFWO has a tolerance factor t = 1.059, which falls into the range of 0.985 < t < 1.06 where no octahedral tilts should occur^53,54^. The superstructure reflections could be generated by antiparallel Pb cationic displacements in perovskite compounds like PbZrO_3_ with an orthorhombic symmetry, but not in the framework of a cubic symmetry. As a rule theoe compounds are antiferroelectrics while PFWO is ferroelectric relaxor. Thus, the oxygen tilting and Pb-cation displacements can be excluded as a source responsible for the observed superlattice reflections of PFWO. It is also important to point out that even if the reflections related with oxygen sublattice were allowed, this would not mean that the intensity of the superstructure reflection in X-ray diffraction experiment could be sufficiently strong to be resolved from the background. In fact, the reflections related with minor oxygen nonstoichiometry are expected to be particularly weak.

Therefore, based on the above considerations, the origin of the observed superlattice reflections must be attributed to the chemical ordering of Fe/W via concentration gradient between *4a* and *4b* oxygen octahedra.

### Temperature evolution of lattice parameters

**Figure 6:**
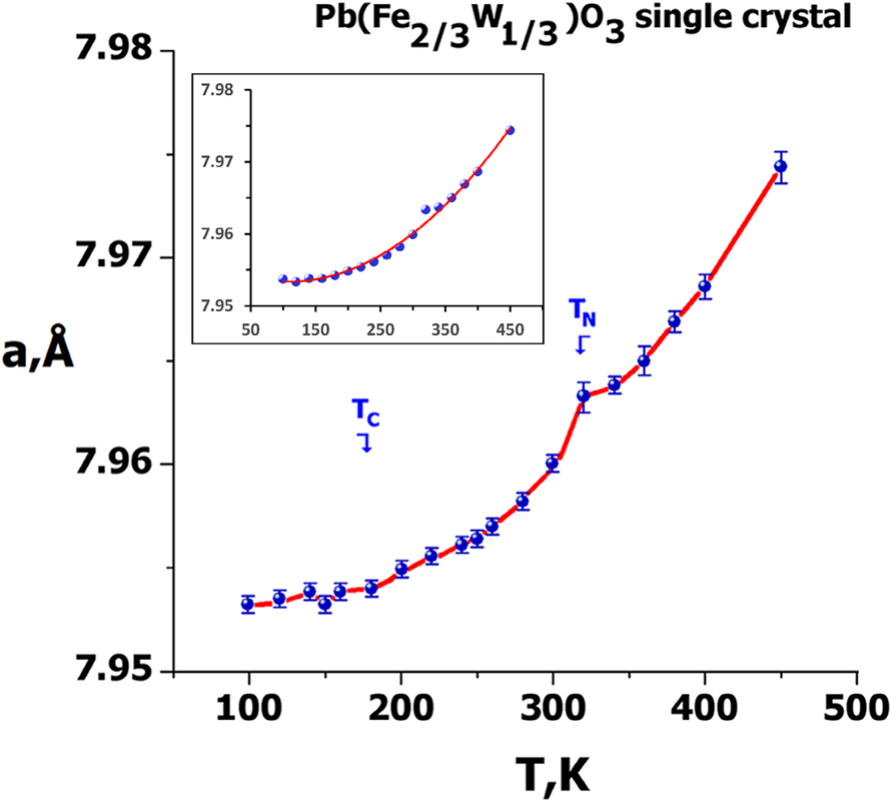
Thermal evolution of the lattice parameter of PFWO. Insert: polynomial approximation of experimental points.

In addition to the extended structural data collected at 100, 280 and 400 K, relatively short runs were carried out in small increments between the end temperature points. The high quality of the data sets allowed us to obtain very accurate temperature evolution of lattice parameters over the entire temperature range examined. The temperature dependence of the lattice constant is plotted in Fig. 6 where the temperature of the maximum dielectric permittivity of relaxor ferroelectric behavior (T_max_ or T_C_) and the antiferromagnetic Neel temperature (T_N_), respectively^14,15,55^, are indicated. Below T_max_ = 180 K, the lattice parameter remains roughly constant with an abnormally weak thermal expansion (< 2 × 10^−6^ K^−l^), revealing the existence of spontaneous strain near 180 K. The thermal expansion coefficient above T_N_ is practically constant and it is equal to 2 × 10^−5^ K^−l^, a value generally found in other Pb-based perovskite oxides^56^.

The temperature evolution of lattice parameters is consistent with our previous finding of thermal expansion from neutron powder diffraction data^13^. However, it is important to note that lattice parameters from X-ray diffraction experiments are even more precise in most cases.

Based on the available data it is difficult to speculate about the anomalies near T_max_ and T_C_ (see inset on Fig. 6 for polynomial approximation of the experimental points) and additional measurements with a small temperature interval must be performed in order to give a definite answer. It is possible to discern only some small abnormal changes in the temperature curve of lattice parameter a(T) quite close to T_C_ and T_N_, and it is more evident near T_N_. The possible structural anomalies near T_C_ and T_max_ in PFWO, if confirmed, could be attributed to the additional strains arising from electrostriction near T_max_ and spin–lattice coupling (magnetostriction) near T_C_. The detailed mechanisms of such electro- and magneto-strictive effects need to be studied in more detail.

### TEM investigation

Using long-range techniques such as X-ray diffraction, only the average periodic structure is determined, and the complementary use of electron diffraction and microscopy is mandatory to achieve a more accurate and more complete structural model for complex materials. Thus, in this work, complementary information is obtained by the TEM techniques via selected area electron diffraction (SAED), dark field (DF) imaging and high-resolution TEM (HRTEM).

**Figure 7:**
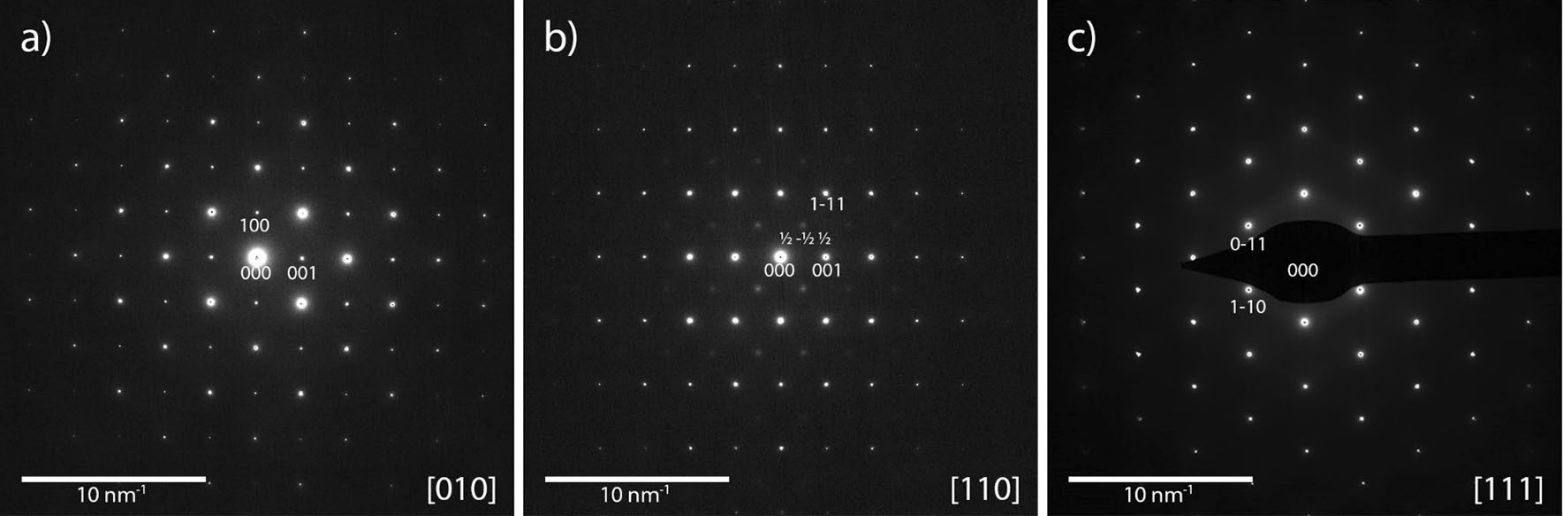
SAED patterns of (**a**) [010]-zone axis, (**b**) [110]-zone axis showing the [− ½ ½ ½] superlattice reflections, and (**c**) [111]-zone axis.

**Figure 8:**
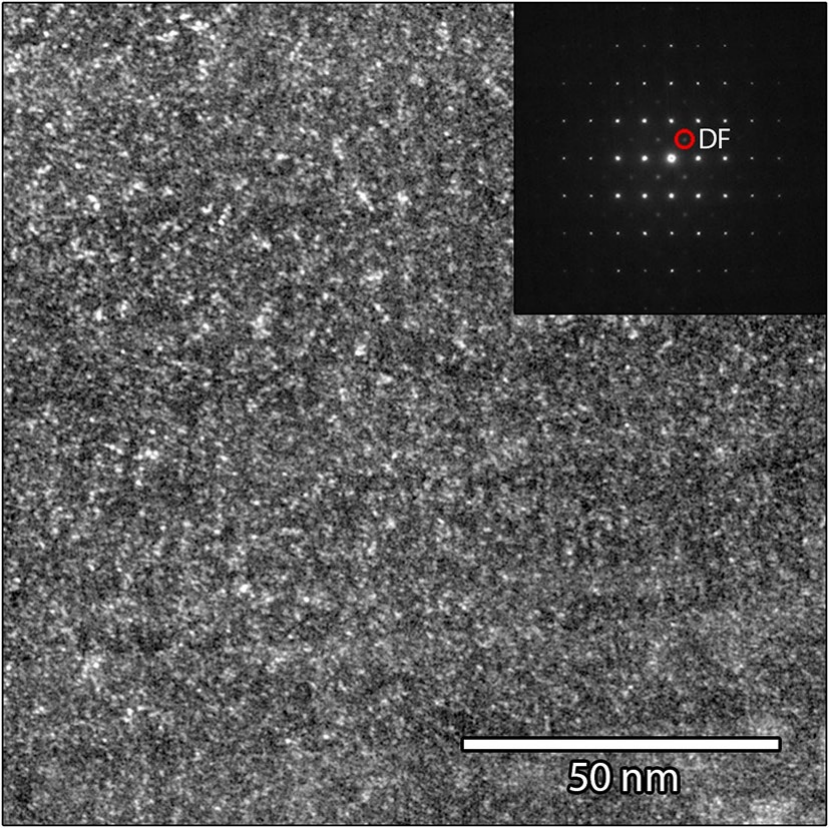
Dark field image using the ½ ½ ½ type superlattice reflection as indicated in the inset showing the electron diffraction pattern. The bright contrast represents the domains with the *Fm* − *3m* ordering that cause the appearance of the superstructural reflections.

The SAED patterns of the linear independent zone axis orientations [010], [110] and [111] are depicted in Fig. 7a–c, respectively. All the patterns are indexed according to the cubic perovskite structure *Pm* − *3m*. In agreement with the results of the XRD analysis, the [110] zone axis pattern shows the (h + ½, k + ½, l + ½) type superlattice reflections, confirming the presence of an actual *Fm* − *3m* symmetry. To further investigate the domain size with *Fm* − *3m* symmetry, i.e. coherence length of Fe and W cations ordering on the B lattice sites, a dark field image using a single (h + ½, k + ½, l + ½) type superlattice reflection was acquired, as depicted in Fig. 8. The bright contrast resembles the domains with Fe and W cation ordering that causes the selected (h + ½, k + ½, l + ½) type superlattice reflections. By measuring the excited areas in the DF image, the domain size is estimated to be between one and two nanometers.

**Figure 9:**
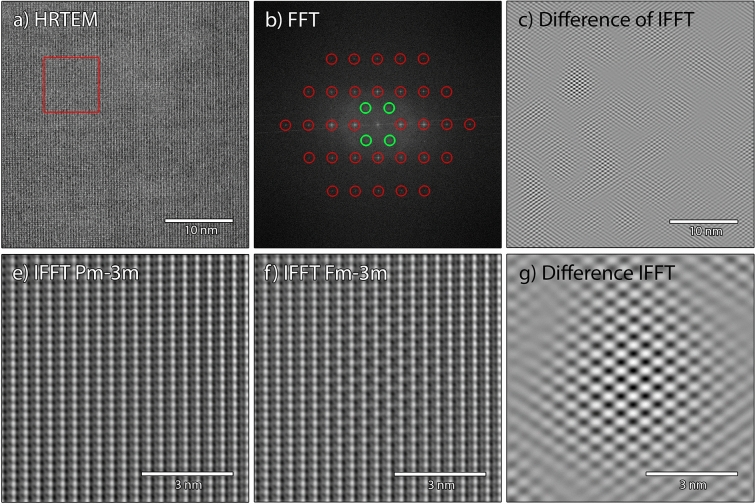
Fourier analysis of the HRTEM image in the [110] orientation. (**a**) unprocessed HRTEM image and (**b**) fast Fourier transform of HRTEM image. The frequencies marked with red circles describe the *Pm* − *3m* symmetry. The weak frequencies marked with green circles are caused by the *Fm* − *3m* ordering in the lattice. Areas depicted in (**e**) and (**f**) are Fourier filtered images using only the frequencies from the *Pm* − *3m* symmetry and with additional *Fm* − *3m* frequencies, respectively. The areas are taken from the position marked by the red box in the HRTEM image. (**c**) and (**g**) are differential images subtracting the *Pm* − *3m* from the *Fm* − *3m* filtered images. Areas with clear two-dimensional periodicity can be used to estimate the domain size with the *Fm* − *3m* symmetry.

To get complimentary information about the *Fm* − *3m* ordering in the *Pm* − *3m* lattice, the HRTEM micrograph depicted in Fig. 9a was analyzed by Fourier filtering. The fast Fourier transform of the HRTEM image is shown in Fig. 9b. In addition to the spatial frequencies of the *Pm* − *3m* symmetry (marked by red circles), weak frequencies caused by the *Fm* − *3m* symmetry are visible (marked with green circles). This reveals that the HRTEM image contains information about the *Fm* − *3m* ordering in the lattice, corroborating the results mentioned above. To extract this information, two Fourier filtered images were created by inverse Fourier transformation, one selecting the spatial frequencies of *Pm* − *3m* symmetry only (using a mask resembling the red circles) and the other with the additional information about *Fm* − *3m* (using a mask resembling the red and green circles). Subtracting the two images from each other exposes the *Fm* − *3m* domains where a clear two dimensional periodicity appears, as depicted in Fig. 9c. For better visibility, magnified areas of the Fourier filtered images using the *Pm* − *3m* and *Fm* − *3m* frequencies, and the differential image are depicted in Fig. 9e–g, respectively. The areas were taken from the region marked with a red square in the original HRTEM image (Fig. 9a). The domain with the *Fm* − *3m* ordering depicted in Fig. 9g has a diameter of 4 nm to 5 nm and is slightly larger than what was estimated by the contrast in the dark field image. The difference might be explained by the fact that both images represent a projection of a volume on a two dimensional image since the TEM sample has a finite thickness. This makes exact measurements difficult to obtain and only possible for very thin samples in the order of the domain itself. The thickness of the TEM sample is unknown, but certainly exceeds the dimensions of the *Fm* − *3m* domain.

**Figure 10:**
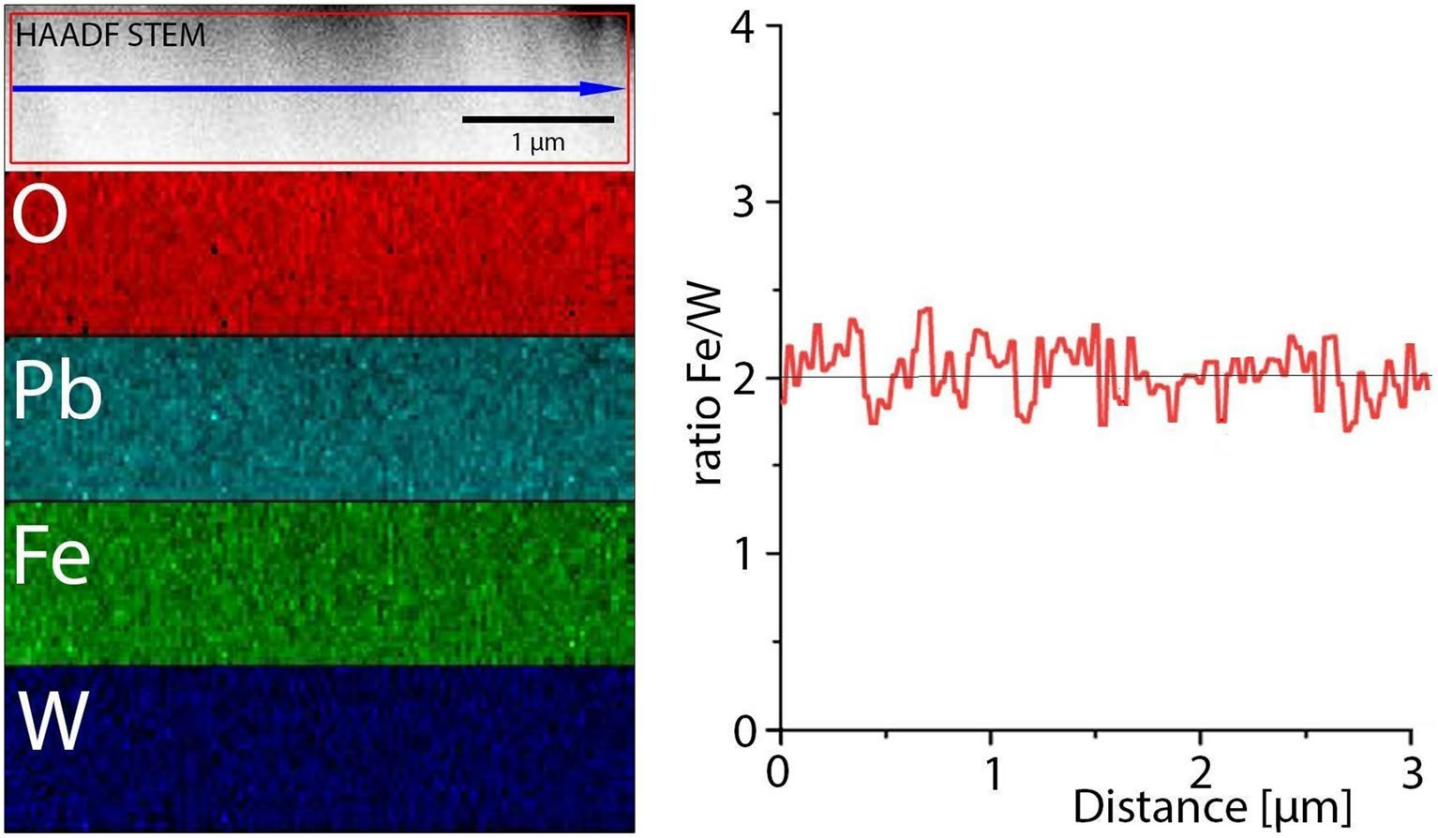
EDX mapping of the Pb(Fe_2/3_W_1/3_)O_3_ Perovskite. The line scan of the Fe/W ratio has been extracted from the map along the blue line and integrated over the area of the red box as show in the upper HAADF STEM image.

Figure 10 shows the result of the EDX analysis in terms of colored elemental maps, showing the elemental distributions according to the labels. No apparent intensity change is observed in the elemental maps for the different elements, testifying a uniform elemental distribution. The line scan was extracted along the blue line as shown in the upper HAADF STEM image and integrated within the area marked by the red box. The concentration of the different elements appears to be uniform along the line and the ratio of Fe/W is closed to 2. Deviation from this nominal value could be related with Pb deficiency and the valence balance rule of cations in the PFWO structure. Some differences from the results of the XRF analysis can be attributed to the standardless quantification in the EDX spectroscopy using theoretical Cliff–Lorimer factors provided by the software ESPRIT 1.9.

The main results of TEM study include (1) in agreement with X-ray diffraction study, a long range order that can be described with the *Fm* − *3m* symmetry is detected, (2) the coherence length of that long range order is in the order of 1–2 nm and (3) no remarkable chemical inhomogeneity is found in the tested PFWO crystal, excluding the possibility of a compositional ordering arising from substitutional defects in the simple perovskite structure.

## Discussion

It is well known that the order–disorder phase transition behavior in Pb-based perovskites can be attributed to a number of factors, such as compositional and (or) thermal fluctuations, structural defects, etc. To some extent, the degree of cation ordering can be modified by appropriate synthesis conditions and/or subsequent thermal treatments, which has significant effects on the physical properties of such relaxor compounds as PFWO^57–60^. Therefore, understanding the mechanism of cationic order–disorder phase transition and determining the phase transition temperature are of great importance for tuning the properties of PFWO. Atomic ordering in Pb-containing perovskites has been a topic of intensive studies for a number of decades, but the majority of the work was carried out on experimental studies of the effects of the ordering on the dielectric properties of the compounds. Thus, fewer publications dealt with crystal chemical mechanisms for the appearance of cation ordering, while taking into account the structure and the cationic sizes and charges.

The difference in the charges between the B’ and B” cations is the key factor influencing cationic ordering^57^. If the difference in the oxidation states is two or less, all the ordering possibilities (i.e. complete rock-salt ordering, partial rock-salt ordering and complete disorder) are possible, dominated by the completely disordered and the partially ordered arrangements though^57^. If the difference is larger than two, highly ordered structures are generally observed. Hence, PFWO can have different ordering arrangements. Other factors, such as the preparation conditions, ionization potentials, etc., could also affect the degree of ordering^56–59^. In addition, cationic coordination geometry and the size ratio of A-cation/B-cation could also possibly influence the degree of ordering^57–60^. Interestingly, in many studied PFWO ceramics a random distribution of cations over the octahedral site was found. Obviously, the criteria mentioned above, though generally valid, may not apply to some complex perovskites (including PFWO). According to Galasso^61^, the ratio s = r_B1_ − r_B2_/r_B1_ determines the ordering in complex perovskites: if s is greater than 0.09, the cationic distribution should be ordered. Although it applies to many perovskites, PFWO (s = 0.07) is rare exception.

The degree of long-range ordering is clearly related to the size ratio of A/B. Generally speaking, the disorder on the B cationic positions in double perovskites results in regions where the sets of adjacent highly charged cations (W^6+^ for PFWO) give rise to electrostatic repulsion that promotes the long-range ordering. This is realized when the temperature is high enough to cause the cationic diffusion. In PFWO, the repulsion between the highly charged W^6+^ cations is partially shielded by the Fe^3+^ cations of double amount, such that the above-mentioned electrostatic driving force for long-range ordering is significant only when the distances between the B cations are sufficiently short. In this process, the A cation size plays an importance role, as it affects the unit-cell size and thereby the distances between the B cations. In comparison, for the Sr-based counterpart, the B–B bond lengths are sizably smaller than those observed in the corresponding PFWO compound, which explains why Sr_3_Fe_2_WO_9_ exhibits a higher degree of long-range ordering^62^. The same tendency was found in Ca_3_Fe_2_WO_9_ where the W^6+^  − W^6+^ electrostatic repulsions become even more significant^63^.

Information about antiferromagnetism in PFWO was obtained by simulations [see the details in Refs.^13,64^, assuming different degrees of disorder between the Fe^3+^ and W^6+^ ions in the octahedral sites of the PFWO lattice. The value of T_N_ was calculated to be 355 K at S = 1 (full ordered, with formula Pb[Fe]_0*.*5_[Fe_1*/*3_W_2*/*3_]_0*.*5_O_3_) and 406 K^18^ or 425 K^64^ (full disordered, with formula Pb[Fe_2*/*3_W_1*/*3_]_0*.*5_[Fe_2*/*3_W_1*/*3_]_0*.*5_O_3_)*.* The experimental value of T_N_, obtained in^13,14^ for our crystals, was found to be 350–363 K, which would suggest the possibility of a fully ordered structure in PFWO. Nevertheless, it is most probable that the ordering in PFWO is only partial. Even if the sublattice composition would correspond to the formula with *S* = 1, there could be disorder mainly in the second sublattice. So, it is more correct to write the structural formula of PFWO as Pb[Fe_1−x_W_x_]_0*.*5_[Fe_1*/*3+x_W_2*/*3−x_]_0*.*5_O_3_*,* where 0 < x < 1*/*3. It is evident that if the cationic ordering degree is different, the properties (such as temperatures transitions, relaxor ferroelectric behavior and magnetic proprieties) will be also different. The differences between the calculated and experimental values of T_N_, and between the different ceramic samples prepared by different methods can be connected with different degrees of partial ion ordering in PFWO.

Our attempts to make a ferrimagnet PFWO based on the unequal occupation by magnetic Fe cations of two distinct but similar sites have succeeded. In PFWO there is a partial mixing of the cations such that the *4a* and *4b* sites are only 69% and 64% occupied by Fe^3+^. This is not surprising given that there are smaller differences in size between Fe^3+^ (0.645 A) and W^6+^ (0.6 A). The cation ordering in PFWO can be enhanced by both thermal treatment (thermal annealing and quenching) and cation substitution^37–48^. In Ref.^3^, we can find the Mössbauer spectrum of PFWO consisting of two sextets observed in the phase pure ceramics fired in oxygen, while the spectrum consists of a single sextet for the sample fired in air. The existence of a single sextet was due to fully disordered arrangement of Fe^3+^ ions, while the two sextets were associated with a partial ordering of the Fe^3+^ and W^6+^ cations. The formation of two magnetic subsystems might be due to nonequivalent surroundings of the iron ions in the partially ordered and disordered regions.

We now discuss the structural phase transition *Pm* − *3m* → *Fm* − *3m*. According to group theoretical prediction the ordered structure is generated by a one-component order parameter ^65^. At the indicated phase transition, two processes associated with the same order parameter will be realized: *B-*cations in the ordered structure are divided into two types (transformation of positions *1b* to positions *4a* and *4b*) and displacement in the anionic sublattice (from position *3c* to position *24e*). From the X-ray diffraction data it is more convenient to consider the occupation factor of B-sites as an order parameter, but from the point of view of group theoretical analysis these two processes (ordering in the B-sublattice and the indicated displacement of the anions) are absolutely equivalent. The change in the cell volume is a consequence of the ordering of cations in the B sublattice^66^.

The actual structural mechanism of the transformation in PFWO remains unknown and the important factors for triggering the *Pm* − *3m* → *Fm* − *3m* transformation are still not fully clear. The transformation occurs by a dilatational mechanism owing to a uniform distortion of the simple cubic lattice by extension of the structure along the main crystallographic directions. Probably, there is no remarkable energy barrier for such a transformation because the cation coordination number is not changing. The nature of phase transformation which could be related with oxygen stoichiometry requires additional investigation.

## Conclusions

A combination of synchrotron X-ray single crystal diffraction and high-resolution electron microscopy techniques has allowed us to reveal the partial ordering in the crystal structure of complex perovskite PFWO for the first time. It is found that the title compound crystallizes in a cubic symmetry with the *Fm* − *3m* space group, showing some degree of long-range ordering. The B-type of cation stacking sequence was experimentally found as the following: (0.69Fe + 0.31 W) − (0.64Fe + 0.36 W) − (0.69Fe + 0.31 W). The presence of some degree of long-range ordering is believed to be related with the smaller unit cell and B–B distances, promoting the electrostatic repulsions between highly charged W^6+^ cations which is the driving force for the long range B-site ordering.

Successful attempts have been made to form a ferrimagnet PFWO based on the unequal occupation by magnetic Fe cations of two distinct but similar sites. The 6s^2^ lone pair of Pb^2+^ cations and the strong covalent character of Pb–O bonds stabilize the ordered structure. Because the partial structural order cannot induce complete chemical order, the dielectric properties of PFWO exhibit a typical relaxor ferroelectric behavior. Our finding of the partial cationic ordering in PFWO provides a clearer picture of the crystal chemistry of Pb-based complex perovskites, which in turn will help understand and tune their multifunctional properties.

### Methods

#### Crystal growth

Single crystals of PFWO were grown from high temperature solution using PbO or (PbO–B_2_O_3_) as flux by a slow cooling from 1,030 to 850 °C at 1–5 °C/h. The growth results strongly depend on the flux composition. The presence of B_2_O_3_ not only increases the dissolving power of the melt, but also gives an optimum degree of complex formation and viscosity^23,24^. The addition of small amounts of B_2_O_3_ (up to 1 wt%) to the PbO flux gives rise to single crystals of pure PFWO showing clean facets with {100}growth steps and a good optical isotropy without inclusions or internal stress, hence suitable for the subsequent physical characterization. More details on the PFWO crystal growth can be found in Refs.^23,24^.

As the crystals are grown from high temperatures, a disordered structure was expected to form first. During the subsequent slow cooling process, the ordering then starts via a diffusion mechanism. Thus, some degree of ordering may be expected to be present even in the as-grown crystals of PFWO. The actual degree of ordering for as-grown crystals strongly depends upon the time of crystallization to achieve an equilibrium degree of order–order via diffusion.

### Analysis of chemical composition

An ICP-AES Jobin–Yvon JY 70 spectrometer was used to determine the composition of single crystals. The PFWO crystals were fused with LiBO_2_ and dissolved in HNO_3_. Ten measurements per sample were made and the average values were obtained with standard deviation (SD). The typical analytical precision was better than 1% of the measured values. The total amount of impurity cations did not exceed 0.06 atom %. According to the elemental analyses carried out on different crystallites, the cationic concentrations in the PFWO samples were found to be Pb_0.98(2)_Fe_0.67(2)_W_0.35(2)_O_3_ (the total number of cations was normalized to be 2). These values are very close to the expected ratios and permit us to conclude that the composition of the samples is very close to the nominal stoichiometry.

A PANalytical Epsilon 3^XLE^ EDXRF spectrometer was also used to measure the cationic concentrations of the samples before X-ray diffraction experiments. This spectrometer is equipped with a high-resolution SDD-10 Detector, a 50 kV 3 mA rhodium anode X-ray tube, a helium purge, a spinner, 6 filters and a 10-position removable sample changer. Each powder sample was transferred into a sample cup assembled with a high transmission Prolene supporting foil (4 µm). All analyses were conducted in a helium/air environment and the total counting time was 15 min. The oxygen content in the samples was monitored by means of iodometric titration. The final composition of the single crystal samples was found to be very close to stoichiometric composition, Pb_0.99(3)_Fe_0.68(3)_W_0.33(3)_O_δ_, with the oxygen content δ = 2.992(7)–3.005(7), within the uncertainty of the iodometric titration.

### Synchrotron X-ray diffraction

A prismatic single crystal was mounted on a 50 µm glass fiber tip with Epoxy glue. The crystal size is available from the CIF files. The intensity data were collected at the Advanced Photon Source on Beamline 15ID-D of NSF’s ChemMatCARS Sector 15 using Huber 3 circles diffractometer with kappa angle offset 60 and equipped with Pilatus3X 1 M (CdTe) detector at temperature of 100(1) K with an Oxford Cryojet. An unattenuated beam with a wavelength of 0.41328 Å (30 keV) was used with 0.3–1.0 s exposure times, and data were collected with multiple ϕ scans at 0.2–0.3° increments with ω and κ offsets using a 13 cm detector distance. The samples were measured at 100 K from 0 to 360° in phi, counting at 0.3 s/frame, in steps of 0.3°/frame at two separate omega positions (− 180°, − 140°). The samples were then heated to 280 K and later to 450 K. Full data sets for structural refinement were collected at 100, 280, and 400 K using counts at 1.0 s/frame, in increments of 0.2°/frame. To improve the statistical reliability of the measured data, each frame was measured four times and merged into one frame.

Evaluation of the initial X-ray diffraction (XRD) images and reciprocal lattice construction was performed to ascertain the crystal quality. Data images were converted to Bruker format and integration was performed with APEX II^67^ suite software. Data reduction was performed using SAINT v.8.32B software. Scaling and absorption correction was performed by a multi-scan method implemented in SADABS v.2013 program included in the APEX suite. The structural solution and refinements were carried out with SHELX-2014^68,69^ software using the XPREP utility for the space group determination, and the XT and XL programs for the structural resolution and refinement, respectively. For all of the structures, the metal and oxygen atoms were found from Fourier difference map and refined anisotropically and in each case the occupancy was determined through free refinement.

### Transmission electron microscopy

Two samples were prepared for transmission electron microscopy (TEM) studies using a focused ion beam and scanning electron microscope (FIB-SEM, FEI Strata DB235). To obtain samples in the [010], [110] and [111] zone axis orientations, the single crystals were roughly oriented in the FIB-SEM with respect to its facetation. The extracted lamellas were then attached to a Cu lift-out grid and thinned to electron transparency with a final polishing step using a 3 kV Ga beam.

Selected area electron diffraction (SAED), dark field imaging, HRTEM and energy dispersive X-ray (EDX) spectroscopy analyses were conducted on a probe corrected FEI Titan Themis S/TEM operated at 200 kV and equipped with the four detector SuperX EDX system. The acquired spectral image was quantified with a standardless method using theoretical Cliff–Lorimer factors provided by the ESPRIT 1.9 software developed by Bruker.

## Supplementary information

Supplementary Information 1.
