# Supplementary material for: Structure of Pb(Fe2/3W1/3)O3 single crystals with partial cation order
Source: Sci Rep. 2020 Sep 3;10:14567. doi: 10.1038/s41598-020-71438-4 (PMC7471969; doi:10.1038/s41598-020-71438-4)
Supplement: Supplementary file 1 — Supplementary Information 1. [file 41598_2020_71438_MOESM1_ESM.pdf]

Scientific Reports

Supplementary Materials

for

Structure of $Pb(Fe_{2/3}W_{1/3})O_3$ single crystals with partial cation order

S. A. Ivanov[a,b] * A. I. Stash[c,d], L. Riekehr[e], Y. S. Chen[d] and Z.-G. Ye[f] *

[a] Department of Chemistry, M.V. Lomonosov Moscow State University, Leninskie Gory 1/3, Moscow 119991, Russia

[b] Department of Engineering Sciences, Solid State Physics. Angstrom Laboratory, Uppsala University, Box 534, SE-751 21 Uppsala, Sweden

[c] A. N. Nesmeyanov Institute of Organoelement Compounds of Russian Academy of Science, 119991, Vavilov st., 28, Moscow, Russia

[d] ChemMatCARS Beamline, The University of Chicago, Advanced Photon Source, Argonne, Illinois 60439, United States

[e] Department of Engineering Sciences, Solid State Electronics. Angstrom Laboratory, Uppsala University, Box 534, SE-751 21 Uppsala, Sweden

[f] Department of Chemistry and 4D LABS, Simon Fraser University, 8888 University Drive, Burnaby, B.C., V5A 1S6, Canada

* Correspondence and requests for materials should be addressed to S. A. I. (isa@angstrom.uu.se) and Z.-G.Y. (zye@sfu.ca)

Table of Contents:

1) Structural data of $Pb(Fe_{2/3}W_{1/3})O_3$ at 100 K, "data_PbFeWO_100", in CIF format (pp. 2-18).

2) Structural data of $Pb(Fe_{2/3}W_{1/3})O_3$ at 280 K, "data_PbFeWO_280", in CIF format (pp. 19-35).

3) Structural data of $Pb(Fe_{2/3}W_{1/3})O_3$ at 400 K, "data_PbFeWO_400", in CIF format (pp. 36-52).

```
data_PbFeWO_100

_audit_creation_method            'SHELXL-2014/7'
_chemical_name_systematic         ?
_chemical_name_common             ?
_chemical_melting_point           ?
_chemical_formula_moiety          ?
_chemical_formula_sum
 'Fe0.67 O3 Pb W0.33'
_chemical_formula_weight          353.60

loop_
 _atom_type_symbol
 _atom_type_description
 _atom_type_scat_dispersion_real
 _atom_type_scat_dispersion_imag
 _atom_type_scat_source
 'O'  'O'   0.0017   0.0018
 'International Tables Vol C Tables 4.2.6.8 and 6.1.1.4'
 'Fe'  'Fe'   0.1986   0.3065
 'International Tables Vol C Tables 4.2.6.8 and 6.1.1.4'
 'W'  'W'  -0.4869   2.7805
 'International Tables Vol C Tables 4.2.6.8 and 6.1.1.4'
 'Pb'  'Pb'  -0.5384   4.2194
 'International Tables Vol C Tables 4.2.6.8 and 6.1.1.4'

_space_group_crystal_system       cubic
_space_group_IT_number            225
_space_group_name_H-M_alt         'F m -3 m'
_space_group_name_Hall            '-F 4 2 3'

_shelx_space_group_comment
;
The symmetry employed for this shelxl refinement is uniquely
defined
by the following loop, which should always be used as a source of
symmetry information in preference to the above space-group names.
They are only intended as comments.
;

loop_
 _space_group_symop_operation_xyz
 'x, y, z'
 '-x, -y, z'
 '-x, y, -z'
 'x, -y, -z'
 'z, x, y'
 'z, -x, -y'
 '-z, -x, y'
 '-z, x, -y'
 'y, z, x'
 '-y, z, -x'
 'y, -z, -x'
```

```
'-y, -z, x'
'y, x, -z'
'-y, -x, -z'
'y, -x, z'
'-y, x, z'
'x, z, -y'
'-x, z, y'
'-x, -z, -y'
'x, -z, y'
'z, y, -x'
'z, -y, x'
'-z, y, x'
'-z, -y, -x'
'x, y+1/2, z+1/2'
'-x, -y+1/2, z+1/2'
'-x, y+1/2, -z+1/2'
'x, -y+1/2, -z+1/2'
'z, x+1/2, y+1/2'
'z, -x+1/2, -y+1/2'
'-z, -x+1/2, y+1/2'
'-z, x+1/2, -y+1/2'
'y, z+1/2, x+1/2'
'-y, z+1/2, -x+1/2'
'y, -z+1/2, -x+1/2'
'-y, -z+1/2, x+1/2'
'y, x+1/2, -z+1/2'
'-y, -x+1/2, -z+1/2'
'y, -x+1/2, z+1/2'
'-y, x+1/2, z+1/2'
'x, z+1/2, -y+1/2'
'-x, z+1/2, y+1/2'
'-x, -z+1/2, -y+1/2'
'x, -z+1/2, y+1/2'
'z, y+1/2, -x+1/2'
'z, -y+1/2, x+1/2'
'-z, y+1/2, x+1/2'
'-z, -y+1/2, -x+1/2'
'x+1/2, y, z+1/2'
'-x+1/2, -y, z+1/2'
'-x+1/2, y, -z+1/2'
'x+1/2, -y, -z+1/2'
'z+1/2, x, y+1/2'
'z+1/2, -x, -y+1/2'
'-z+1/2, -x, y+1/2'
'-z+1/2, x, -y+1/2'
'y+1/2, z, x+1/2'
'-y+1/2, z, -x+1/2'
'y+1/2, -z, -x+1/2'
'-y+1/2, -z, x+1/2'
'y+1/2, x, -z+1/2'
'-y+1/2, -x, -z+1/2'
'y+1/2, -x, z+1/2'
'-y+1/2, x, z+1/2'
'x+1/2, z, -y+1/2'
```

```
'-x+1/2, z, y+1/2'
'-x+1/2, -z, -y+1/2'
'x+1/2, -z, y+1/2'
'z+1/2, y, -x+1/2'
'z+1/2, -y, x+1/2'
'-z+1/2, y, x+1/2'
'-z+1/2, -y, -x+1/2'
'x+1/2, y+1/2, z'
'-x+1/2, -y+1/2, z'
'-x+1/2, y+1/2, -z'
'x+1/2, -y+1/2, -z'
'z+1/2, x+1/2, y'
'z+1/2, -x+1/2, -y'
'-z+1/2, -x+1/2, y'
'-z+1/2, x+1/2, -y'
'y+1/2, z+1/2, x'
'-y+1/2, z+1/2, -x'
'y+1/2, -z+1/2, -x'
'-y+1/2, -z+1/2, x'
'y+1/2, x+1/2, -z'
'-y+1/2, -x+1/2, -z'
'y+1/2, -x+1/2, z'
'-y+1/2, x+1/2, z'
'x+1/2, z+1/2, -y'
'-x+1/2, z+1/2, y'
'-x+1/2, -z+1/2, -y'
'x+1/2, -z+1/2, y'
'z+1/2, y+1/2, -x'
'z+1/2, -y+1/2, x'
'-z+1/2, y+1/2, x'
'-z+1/2, -y+1/2, -x'
'-x, -y, -z'
'x, y, -z'
'x, -y, z'
'-x, y, z'
'-z, -x, -y'
'-z, x, y'
'z, x, -y'
'z, -x, y'
'-y, -z, -x'
'y, -z, x'
'-y, z, x'
'y, z, -x'
'-y, -x, z'
'y, x, z'
'-y, x, -z'
'y, -x, -z'
'-x, -z, y'
'x, -z, -y'
'x, z, y'
'-x, z, -y'
'-z, -y, x'
'-z, y, -x'
'z, -y, -x'
```

'z, y, x'
'-x, -y+1/2, -z+1/2'
'x, y+1/2, -z+1/2'
'x, -y+1/2, z+1/2'
'-x, y+1/2, z+1/2'
'-z, -x+1/2, -y+1/2'
'-z, x+1/2, y+1/2'
'z, x+1/2, -y+1/2'
'z, -x+1/2, y+1/2'
'-y, -z+1/2, -x+1/2'
'y, -z+1/2, x+1/2'
'-y, z+1/2, x+1/2'
'y, z+1/2, -x+1/2'
'-y, -x+1/2, z+1/2'
'y, x+1/2, z+1/2'
'-y, x+1/2, -z+1/2'
'y, -x+1/2, -z+1/2'
'-x, -z+1/2, y+1/2'
'x, -z+1/2, -y+1/2'
'x, z+1/2, y+1/2'
'-x, z+1/2, -y+1/2'
'-z, -y+1/2, x+1/2'
'-z, y+1/2, -x+1/2'
'z, -y+1/2, -x+1/2'
'z, y+1/2, x+1/2'
'-x+1/2, -y, -z+1/2'
'x+1/2, y, -z+1/2'
'x+1/2, -y, z+1/2'
'-x+1/2, y, z+1/2'
'-z+1/2, -x, -y+1/2'
'-z+1/2, x, y+1/2'
'z+1/2, x, -y+1/2'
'z+1/2, -x, y+1/2'
'-y+1/2, -z, -x+1/2'
'y+1/2, -z, x+1/2'
'-y+1/2, z, x+1/2'
'y+1/2, z, -x+1/2'
'-y+1/2, -x, z+1/2'
'y+1/2, x, z+1/2'
'-y+1/2, x, -z+1/2'
'y+1/2, -x, -z+1/2'
'-x+1/2, -z, y+1/2'
'x+1/2, -z, -y+1/2'
'x+1/2, z, y+1/2'
'-x+1/2, z, -y+1/2'
'-z+1/2, -y, x+1/2'
'-z+1/2, y, -x+1/2'
'z+1/2, -y, -x+1/2'
'z+1/2, y, x+1/2'
'-x+1/2, -y+1/2, -z'
'x+1/2, y+1/2, -z'
'x+1/2, -y+1/2, z'
'-x+1/2, y+1/2, z'
'-z+1/2, -x+1/2, -y'

```
 '-z+1/2, x+1/2, y'
 'z+1/2, x+1/2, -y'
 'z+1/2, -x+1/2, y'
 '-y+1/2, -z+1/2, -x'
 'y+1/2, -z+1/2, x'
 '-y+1/2, z+1/2, x'
 'y+1/2, z+1/2, -x'
 '-y+1/2, -x+1/2, z'
 'y+1/2, x+1/2, z'
 '-y+1/2, x+1/2, -z'
 'y+1/2, -x+1/2, -z'
 '-x+1/2, -z+1/2, y'
 'x+1/2, -z+1/2, -y'
 'x+1/2, z+1/2, y'
 '-x+1/2, z+1/2, -y'
 '-z+1/2, -y+1/2, x'
 '-z+1/2, y+1/2, -x'
 'z+1/2, -y+1/2, -x'
 'z+1/2, y+1/2, x'

_cell_length_a                    7.9537(4)
_cell_length_b                    7.9537(4)
_cell_length_c                    7.9537(4)
_cell_angle_alpha                 90
_cell_angle_beta                  90
_cell_angle_gamma                 90
_cell_volume                      503.16(8)
_cell_formula_units_Z             8
_cell_measurement_temperature     100(2)
_cell_measurement_reflns_used     2944
_cell_measurement_theta_min       2.978
_cell_measurement_theta_max       23.523

_exptl_crystal_description        prizm
_exptl_crystal_colour             dark
_exptl_crystal_density_meas       ?
_exptl_crystal_density_method     ?
_exptl_crystal_density_diffrn     9.336
_exptl_crystal_F_000              1184
_exptl_transmission_factor_min    ?
_exptl_transmission_factor_max    ?
_exptl_crystal_size_max           0.070
_exptl_crystal_size_mid           0.020
_exptl_crystal_size_min           0.010
_exptl_absorpt_coefficient_mu     20.899
_shelx_estimated_absorpt_T_min    0.322
_shelx_estimated_absorpt_T_max    0.818
_exptl_absorpt_correction_type    multi-scan
_exptl_absorpt_correction_T_min   0.271
_exptl_absorpt_correction_T_max   0.427
_exptl_absorpt_process_details
;
 SADABS-2014/4 - Bruker AXS area detector scaling
 and absorption correction
```

```
;
_diffrn_ambient_temperature       100(2)
_diffrn_radiation_wavelength      0.41328
_diffrn_radiation_type            'synchrotron'
_diffrn_source                    'Advanced photon source'
_diffrn_radiation_monochromator   'diamond (111)'
_diffrn_measurement_device_type
;
Huber 3 circle diffractometer with PILATUS3 X CdTe 1M detector
;
_diffrn_measurement_method        '\f-scan'
_diffrn_detector_area_resol_mean  ?
_diffrn_standards_number          0
_diffrn_standards_interval_count  0
_diffrn_standards_interval_time   0
_diffrn_standards_decay_%         0.0
_diffrn_reflns_number             1676
_diffrn_reflns_av_unetI/netI      0.0220
_diffrn_reflns_av_R_equivalents   0.0454
_diffrn_reflns_limit_h_min        -14
_diffrn_reflns_limit_h_max        14
_diffrn_reflns_limit_k_min        -11
_diffrn_reflns_limit_k_max        11
_diffrn_reflns_limit_l_min        -13
_diffrn_reflns_limit_l_max        13
_diffrn_reflns_theta_min          2.579
_diffrn_reflns_theta_max          23.097
_diffrn_reflns_theta_full         14.357
_diffrn_measured_fraction_theta_max   0.953
_diffrn_measured_fraction_theta_full  0.975
_diffrn_reflns_Laue_measured_fraction_max    0.953
_diffrn_reflns_Laue_measured_fraction_full   0.975
_diffrn_reflns_point_group_measured_fraction_max   0.953
_diffrn_reflns_point_group_measured_fraction_full  0.975
_reflns_number_total              121
_reflns_number_gt                 117
_reflns_threshold_expression      'I > 2\s(I)'
_reflns_Friedel_coverage          0.000
_reflns_Friedel_fraction_max      .
_reflns_Friedel_fraction_full     .

_reflns_special_details
;
 Reflections were merged by SHELXL according to the crystal
 class for the calculation of statistics and refinement.

 _reflns_Friedel_fraction is defined as the number of unique
 Friedel pairs measured divided by the number that would be
 possible theoretically, ignoring centric projections and
 systematic absences.
;

_computing_cell_refinement        'SAINT V8.32B (Bruker AXS Inc.,
2013)'
```

```
_computing_data_collection        ?
_computing_data_reduction         'SAINT V8.32B (Bruker AXS Inc.,
2013)'
_computing_molecular_graphics     'Bruker SHELXTL (Bruker AXS
Inc., 2013)'
_computing_structure_refinement   'SHELXL-2014/7 (Sheldrick,
2014)'
_computing_structure_solution     'SHELXT-2014/4 (Sheldrick,
2014)'
_computing_publication_material   ?
_refine_special_details           ?
_refine_ls_structure_factor_coef  Fsqd
_refine_ls_matrix_type            full
_refine_ls_weighting_scheme       calc
_refine_ls_weighting_details
'w=1/[\s^2^(Fo^2^)+(0.0299P)^2^+3.7027P] where P=(Fo^2^+2Fc^2^)/3'
_atom_sites_solution_primary      ?
_atom_sites_solution_secondary    ?
_atom_sites_solution_hydrogens    .
_refine_ls_hydrogen_treatment     undef
_refine_ls_extinction_method      'SHELXL-2014/7 (Sheldrick 2014'
_refine_ls_extinction_coef        0.29(3)
_refine_ls_extinction_expression
 'Fc^*^=kFc[1+0.001xFc^2^\l^3^/sin(2\q)]^-1/4^'
_refine_ls_number_reflns          121
_refine_ls_number_parameters      11
_refine_ls_number_restraints      0
_refine_ls_R_factor_all           0.0218
_refine_ls_R_factor_gt            0.0216
_refine_ls_wR_factor_ref          0.0741
_refine_ls_wR_factor_gt           0.0740
_refine_ls_goodness_of_fit_ref    1.289
_refine_ls_restrained_S_all       1.289
_refine_ls_shift/su_max           0.000
_refine_ls_shift/su_mean          0.000

loop_
 _atom_site_label
 _atom_site_type_symbol
 _atom_site_fract_x
 _atom_site_fract_y
 _atom_site_fract_z
 _atom_site_U_iso_or_equiv
 _atom_site_adp_type
 _atom_site_occupancy
 _atom_site_site_symmetry_order
 _atom_site_calc_flag
 _atom_site_refinement_flags_posn
 _atom_site_refinement_flags_adp
 _atom_site_refinement_flags_occupancy
 _atom_site_disorder_assembly
 _atom_site_disorder_group
Pb1 Pb 0.2201(2) 0.2500 0.2500 0.0206(6) Uani 0.1667 4 d S T P . .
W1 W 0.0000 0.0000 0.0000 0.0089(3) Uani 0.3148 48 d S T P . .
```

```
Fe1 Fe 0.0000 0.0000 0.0000 0.0089(3) Uani 0.685 48 d S T P . .
W2 W 0.5000 0.5000 0.5000 0.0082(4) Uani 0.3509 48 d S T P . .
Fe2 Fe 0.5000 0.5000 0.5000 0.0082(4) Uani 0.649 48 d S T P . .
O1 O 0.2502(3) 0.0000 0.0000 0.0163(7) Uani 1 8 d S T P . .

loop_
 _atom_site_aniso_label
 _atom_site_aniso_U_11
 _atom_site_aniso_U_22
 _atom_site_aniso_U_33
 _atom_site_aniso_U_23
 _atom_site_aniso_U_13
 _atom_site_aniso_U_12
Pb1 0.0134(7) 0.0242(6) 0.0242(6) -0.00083(11) 0.000 0.000
W1 0.0089(3) 0.0089(3) 0.0089(3) 0.000 0.000 0.000
Fe1 0.0089(3) 0.0089(3) 0.0089(3) 0.000 0.000 0.000
W2 0.0082(4) 0.0082(4) 0.0082(4) 0.000 0.000 0.000
Fe2 0.0082(4) 0.0082(4) 0.0082(4) 0.000 0.000 0.000
O1 0.0089(14) 0.0200(10) 0.0200(10) 0.000 0.000 0.000

_geom_special_details
;
 All esds (except the esd in the dihedral angle between two l.s.
planes)
 are estimated using the full covariance matrix.  The cell esds
are taken
 into account individually in the estimation of esds in distances,
angles
 and torsion angles; correlations between esds in cell parameters
are only
 used when they are defined by crystal symmetry.  An approximate
(isotropic)
 treatment of cell esds is used for estimating esds involving l.s.
planes.
;

loop_
 _geom_bond_atom_site_label_1
 _geom_bond_atom_site_label_2
 _geom_bond_distance
 _geom_bond_site_symmetry_2
 _geom_bond_publ_flag
Pb1 Pb1 0.336(2) 58 ?
Pb1 Pb1 0.336(2) 30 ?
Pb1 Pb1 0.336(2) 9 ?
Pb1 Pb1 0.336(2) 5 ?
Pb1 Pb1 0.476(3) 74 ?
Pb1 O1 2.6493(10) 9 ?
Pb1 O1 2.6493(10) 5 ?
Pb1 O1 2.6493(10) 129 ?
Pb1 O1 2.6493(10) 125 ?
Pb1 W1 3.3125(8) . ?
Pb1 Fe1 3.3125(8) . ?
Pb1 Fe1 3.3125(8) 25 ?
```

W1 O1 1.990(2) 105 ?
W1 O1 1.990(2) 9 ?
W1 O1 1.990(2) 101 ?
W1 O1 1.990(2) 5 ?
W1 O1 1.990(2) 97 ?
W1 O1 1.990(2) . ?
W1 Pb1 3.3125(8) 101 ?
W1 Pb1 3.3125(8) 5 ?
W1 Pb1 3.3125(8) 97 ?
W1 Pb1 3.3125(8) 105 ?
W1 Pb1 3.3125(8) 9 ?
W1 Pb1 3.3124(8) 25_544 ?
Fe1 O1 1.990(2) 105 ?
Fe1 O1 1.990(2) 9 ?
Fe1 O1 1.990(2) 101 ?
Fe1 O1 1.990(2) 5 ?
Fe1 O1 1.990(2) 97 ?
Fe1 O1 1.990(2) . ?
Fe1 Pb1 3.3125(8) 101 ?
Fe1 Pb1 3.3125(8) 5 ?
Fe1 Pb1 3.3125(8) 97 ?
Fe1 Pb1 3.3125(8) 105 ?
Fe1 Pb1 3.3125(8) 9 ?
Fe1 Pb1 3.3124(8) 25_544 ?
W2 O1 1.987(2) 177_556 ?
W2 O1 1.987(2) 81 ?
W2 O1 1.987(2) 149_565 ?
W2 O1 1.987(2) 53 ?
W2 O1 1.987(2) 121_655 ?
W2 O1 1.987(2) 25 ?
W2 Pb1 3.3125(8) 126_655 ?
W2 Pb1 3.3125(8) 30 ?
W2 Pb1 3.3125(8) 74 ?
W2 Pb1 3.3125(8) 154_565 ?
W2 Pb1 3.3125(8) 58 ?
W2 Pb1 3.3125(8) 170_556 ?
Fe2 O1 1.987(2) 177_556 ?
Fe2 O1 1.987(2) 81 ?
Fe2 O1 1.987(2) 149_565 ?
Fe2 O1 1.987(2) 53 ?
Fe2 O1 1.987(2) 121_655 ?
Fe2 O1 1.987(2) 25 ?
Fe2 Pb1 3.3125(8) 126_655 ?
Fe2 Pb1 3.3125(8) 30 ?
Fe2 Pb1 3.3125(8) 74 ?
Fe2 Pb1 3.3125(8) 154_565 ?
Fe2 Pb1 3.3125(8) 58 ?
Fe2 Pb1 3.3125(8) 170_556 ?
O1 Fe2 1.987(2) 25_544 ?
O1 W2 1.987(2) 25_544 ?
O1 Pb1 2.6492(10) 5 ?
O1 Pb1 2.6492(10) 6 ?
O1 Pb1 2.6492(10) 82_545 ?
O1 Pb1 2.6492(10) 9 ?

```
O1 Pb1 2.6492(10) 150_554 ?
O1 Pb1 2.6492(10) 177 ?
O1 Pb1 2.6492(10) 106 ?
O1 Pb1 2.6492(10) 149 ?

loop_
 _geom_angle_atom_site_label_1
 _geom_angle_atom_site_label_2
 _geom_angle_atom_site_label_3
 _geom_angle
 _geom_angle_site_symmetry_1
 _geom_angle_site_symmetry_3
 _geom_angle_publ_flag
Pb1 Pb1 Pb1 60.002(1) 58 30 ?
Pb1 Pb1 Pb1 90.003(1) 58 9 ?
Pb1 Pb1 Pb1 60.002(1) 30 9 ?
Pb1 Pb1 Pb1 60.0 58 5 ?
Pb1 Pb1 Pb1 90.003(1) 30 5 ?
Pb1 Pb1 Pb1 60.0 9 5 ?
Pb1 Pb1 Pb1 45.0 58 74 ?
Pb1 Pb1 Pb1 45.0 30 74 ?
Pb1 Pb1 Pb1 45.001(1) 9 74 ?
Pb1 Pb1 Pb1 45.001(1) 5 74 ?
Pb1 Pb1 O1 117.83(4) 58 9 ?
Pb1 Pb1 O1 176.36(3) 30 9 ?
Pb1 Pb1 O1 117.88(4) 9 9 ?
Pb1 Pb1 O1 86.36(3) 5 9 ?
Pb1 Pb1 O1 131.36(3) 74 9 ?
Pb1 Pb1 O1 176.36(3) 58 5 ?
Pb1 Pb1 O1 117.83(4) 30 5 ?
Pb1 Pb1 O1 86.36(3) 9 5 ?
Pb1 Pb1 O1 117.88(4) 5 5 ?
Pb1 Pb1 O1 131.36(3) 74 5 ?
O1 Pb1 O1 64.16(8) 9 5 ?
Pb1 Pb1 O1 117.88(4) 58 129 ?
Pb1 Pb1 O1 86.36(3) 30 129 ?
Pb1 Pb1 O1 117.83(4) 9 129 ?
Pb1 Pb1 O1 176.36(2) 5 129 ?
Pb1 Pb1 O1 131.36(3) 74 129 ?
O1 Pb1 O1 97.28(5) 9 129 ?
O1 Pb1 O1 64.06(8) 5 129 ?
Pb1 Pb1 O1 86.36(3) 58 125 ?
Pb1 Pb1 O1 117.88(4) 30 125 ?
Pb1 Pb1 O1 176.36(3) 9 125 ?
Pb1 Pb1 O1 117.83(4) 5 125 ?
Pb1 Pb1 O1 131.36(3) 74 125 ?
O1 Pb1 O1 64.06(8) 9 125 ?
O1 Pb1 O1 97.28(5) 5 125 ?
O1 Pb1 O1 64.16(8) 129 125 ?
Pb1 Pb1 W1 142.956(13) 58 . ?
Pb1 Pb1 W1 142.956(13) 30 . ?
Pb1 Pb1 W1 87.09(2) 9 . ?
Pb1 Pb1 W1 87.09(2) 5 . ?
Pb1 Pb1 W1 121.90(2) 74 . ?
```

O1 Pb1 W1 36.92(5) 9 . ?
O1 Pb1 W1 36.92(5) 5 . ?
O1 Pb1 W1 95.80(5) 129 . ?
O1 Pb1 W1 95.80(5) 125 . ?
Pb1 Pb1 Fe1 142.956(13) 58 . ?
Pb1 Pb1 Fe1 142.956(13) 30 . ?
Pb1 Pb1 Fe1 87.09(2) 9 . ?
Pb1 Pb1 Fe1 87.09(2) 5 . ?
Pb1 Pb1 Fe1 121.90(2) 74 . ?
O1 Pb1 Fe1 36.92(5) 9 . ?
O1 Pb1 Fe1 36.92(5) 5 . ?
O1 Pb1 Fe1 95.80(5) 129 . ?
O1 Pb1 Fe1 95.80(5) 125 . ?
W1 Pb1 Fe1 0.0 . . ?
Pb1 Pb1 Fe1 87.09(2) 58 25 ?
Pb1 Pb1 Fe1 87.09(2) 30 25 ?
Pb1 Pb1 Fe1 142.956(13) 9 25 ?
Pb1 Pb1 Fe1 142.956(13) 5 25 ?
Pb1 Pb1 Fe1 121.90(2) 74 25 ?
O1 Pb1 Fe1 95.80(5) 9 25 ?
O1 Pb1 Fe1 95.80(5) 5 25 ?
O1 Pb1 Fe1 36.92(5) 129 25 ?
O1 Pb1 Fe1 36.92(5) 125 25 ?
W1 Pb1 Fe1 116.19(5) . 25 ?
Fe1 Pb1 Fe1 116.19(5) . 25 ?
O1 W1 O1 180.0 105 9 ?
O1 W1 O1 90.0 105 101 ?
O1 W1 O1 90.0 9 101 ?
O1 W1 O1 90.0 105 5 ?
O1 W1 O1 90.0 9 5 ?
O1 W1 O1 180.0 101 5 ?
O1 W1 O1 90.0 105 97 ?
O1 W1 O1 90.0 9 97 ?
O1 W1 O1 90.0 101 97 ?
O1 W1 O1 90.0 5 97 ?
O1 W1 O1 90.0 105 . ?
O1 W1 O1 90.0 9 . ?
O1 W1 O1 90.0 101 . ?
O1 W1 O1 90.0 5 . ?
O1 W1 O1 180.0 97 . ?
O1 W1 Pb1 53.109(11) 105 101 ?
O1 W1 Pb1 126.891(11) 9 101 ?
O1 W1 Pb1 58.10(2) 101 101 ?
O1 W1 Pb1 121.90(2) 5 101 ?
O1 W1 Pb1 53.109(11) 97 101 ?
O1 W1 Pb1 126.891(11) . 101 ?
O1 W1 Pb1 126.891(11) 105 5 ?
O1 W1 Pb1 53.109(11) 9 5 ?
O1 W1 Pb1 121.90(2) 101 5 ?
O1 W1 Pb1 58.10(2) 5 5 ?
O1 W1 Pb1 126.891(11) 97 5 ?
O1 W1 Pb1 53.109(11) . 5 ?
Pb1 W1 Pb1 180.0 101 5 ?
O1 W1 Pb1 53.109(11) 105 97 ?

O1 W1 Pb1 126.891(11) 9 97 ?
O1 W1 Pb1 53.109(11) 101 97 ?
O1 W1 Pb1 126.891(11) 5 97 ?
O1 W1 Pb1 58.10(2) 97 97 ?
O1 W1 Pb1 121.90(2) . 97 ?
Pb1 W1 Pb1 5.82(4) 101 97 ?
Pb1 W1 Pb1 174.18(4) 5 97 ?
O1 W1 Pb1 58.10(2) 105 105 ?
O1 W1 Pb1 121.90(2) 9 105 ?
O1 W1 Pb1 53.109(11) 101 105 ?
O1 W1 Pb1 126.891(11) 5 105 ?
O1 W1 Pb1 53.109(11) 97 105 ?
O1 W1 Pb1 126.891(11) . 105 ?
Pb1 W1 Pb1 5.82(4) 101 105 ?
Pb1 W1 Pb1 174.18(4) 5 105 ?
Pb1 W1 Pb1 5.82(4) 97 105 ?
O1 W1 Pb1 121.90(2) 105 9 ?
O1 W1 Pb1 58.10(2) 9 9 ?
O1 W1 Pb1 126.891(11) 101 9 ?
O1 W1 Pb1 53.109(11) 5 9 ?
O1 W1 Pb1 126.891(11) 97 9 ?
O1 W1 Pb1 53.109(11) . 9 ?
Pb1 W1 Pb1 174.18(4) 101 9 ?
Pb1 W1 Pb1 5.82(4) 5 9 ?
Pb1 W1 Pb1 174.18(4) 97 9 ?
Pb1 W1 Pb1 180.00(5) 105 9 ?
O1 W1 Pb1 53.109(11) 105 25_544 ?
O1 W1 Pb1 126.891(11) 9 25_544 ?
O1 W1 Pb1 53.109(11) 101 25_544 ?
O1 W1 Pb1 126.891(11) 5 25_544 ?
O1 W1 Pb1 121.90(2) 97 25_544 ?
O1 W1 Pb1 58.10(2) . 25_544 ?
Pb1 W1 Pb1 68.879(11) 101 25_544 ?
Pb1 W1 Pb1 111.121(11) 5 25_544 ?
Pb1 W1 Pb1 63.81(5) 97 25_544 ?
Pb1 W1 Pb1 68.879(11) 105 25_544 ?
Pb1 W1 Pb1 111.121(11) 9 25_544 ?
O1 Fe1 O1 180.0 105 9 ?
O1 Fe1 O1 90.0 105 101 ?
O1 Fe1 O1 90.0 9 101 ?
O1 Fe1 O1 90.0 105 5 ?
O1 Fe1 O1 90.0 9 5 ?
O1 Fe1 O1 180.0 101 5 ?
O1 Fe1 O1 90.0 105 97 ?
O1 Fe1 O1 90.0 9 97 ?
O1 Fe1 O1 90.0 101 97 ?
O1 Fe1 O1 90.0 5 97 ?
O1 Fe1 O1 90.0 105 . ?
O1 Fe1 O1 90.0 9 . ?
O1 Fe1 O1 90.0 101 . ?
O1 Fe1 O1 90.0 5 . ?
O1 Fe1 O1 180.0 97 . ?
O1 Fe1 Pb1 53.109(11) 105 101 ?
O1 Fe1 Pb1 126.891(11) 9 101 ?

```
O1 Fe1 Pb1 58.10(2) 101 101 ?
O1 Fe1 Pb1 121.90(2) 5 101 ?
O1 Fe1 Pb1 53.109(11) 97 101 ?
O1 Fe1 Pb1 126.891(11) . 101 ?
O1 Fe1 Pb1 126.891(11) 105 5 ?
O1 Fe1 Pb1 53.109(11) 9 5 ?
O1 Fe1 Pb1 121.90(2) 101 5 ?
O1 Fe1 Pb1 58.10(2) 5 5 ?
O1 Fe1 Pb1 126.891(11) 97 5 ?
O1 Fe1 Pb1 53.109(11) . 5 ?
Pb1 Fe1 Pb1 180.0 101 5 ?
O1 Fe1 Pb1 53.109(11) 105 97 ?
O1 Fe1 Pb1 126.891(11) 9 97 ?
O1 Fe1 Pb1 53.109(11) 101 97 ?
O1 Fe1 Pb1 126.891(11) 5 97 ?
O1 Fe1 Pb1 58.10(2) 97 97 ?
O1 Fe1 Pb1 121.90(2) . 97 ?
Pb1 Fe1 Pb1 5.82(4) 101 97 ?
Pb1 Fe1 Pb1 174.18(4) 5 97 ?
O1 Fe1 Pb1 58.10(2) 105 105 ?
O1 Fe1 Pb1 121.90(2) 9 105 ?
O1 Fe1 Pb1 53.109(11) 101 105 ?
O1 Fe1 Pb1 126.891(11) 5 105 ?
O1 Fe1 Pb1 53.109(11) 97 105 ?
O1 Fe1 Pb1 126.891(11) . 105 ?
Pb1 Fe1 Pb1 5.82(4) 101 105 ?
Pb1 Fe1 Pb1 174.18(4) 5 105 ?
Pb1 Fe1 Pb1 5.82(4) 97 105 ?
O1 Fe1 Pb1 121.90(2) 105 9 ?
O1 Fe1 Pb1 58.10(2) 9 9 ?
O1 Fe1 Pb1 126.891(11) 101 9 ?
O1 Fe1 Pb1 53.109(11) 5 9 ?
O1 Fe1 Pb1 126.891(11) 97 9 ?
O1 Fe1 Pb1 53.109(11) . 9 ?
Pb1 Fe1 Pb1 174.18(4) 101 9 ?
Pb1 Fe1 Pb1 5.82(4) 5 9 ?
Pb1 Fe1 Pb1 174.18(4) 97 9 ?
Pb1 Fe1 Pb1 180.00(5) 105 9 ?
O1 Fe1 Pb1 53.109(11) 105 25_544 ?
O1 Fe1 Pb1 126.891(11) 9 25_544 ?
O1 Fe1 Pb1 53.109(11) 101 25_544 ?
O1 Fe1 Pb1 126.891(11) 5 25_544 ?
O1 Fe1 Pb1 121.90(2) 97 25_544 ?
O1 Fe1 Pb1 58.10(2) . 25_544 ?
Pb1 Fe1 Pb1 68.879(11) 101 25_544 ?
Pb1 Fe1 Pb1 111.121(11) 5 25_544 ?
Pb1 Fe1 Pb1 63.81(5) 97 25_544 ?
Pb1 Fe1 Pb1 68.879(11) 105 25_544 ?
Pb1 Fe1 Pb1 111.121(11) 9 25_544 ?
O1 W2 O1 180.0 177_556 81 ?
O1 W2 O1 90.0 177_556 149_565 ?
O1 W2 O1 90.0 81 149_565 ?
O1 W2 O1 90.0 177_556 53 ?
O1 W2 O1 90.0 81 53 ?
```

O1 W2 O1 180.0 149_565 53 ?
O1 W2 O1 90.0 177_556 121_655 ?
O1 W2 O1 90.0 81 121_655 ?
O1 W2 O1 90.000(1) 149_565 121_655 ?
O1 W2 O1 90.0 53 121_655 ?
O1 W2 O1 90.0 177_556 25 ?
O1 W2 O1 90.0 81 25 ?
O1 W2 O1 90.0 149_565 25 ?
O1 W2 O1 90.000(1) 53 25 ?
O1 W2 O1 180.0 121_655 25 ?
O1 W2 Pb1 53.109(11) 177_556 126_655 ?
O1 W2 Pb1 126.891(11) 81 126_655 ?
O1 W2 Pb1 58.10(2) 149_565 126_655 ?
O1 W2 Pb1 121.90(2) 53 126_655 ?
O1 W2 Pb1 53.109(11) 121_655 126_655 ?
O1 W2 Pb1 126.891(11) 25 126_655 ?
O1 W2 Pb1 126.891(11) 177_556 30 ?
O1 W2 Pb1 53.109(11) 81 30 ?
O1 W2 Pb1 121.90(2) 149_565 30 ?
O1 W2 Pb1 58.10(2) 53 30 ?
O1 W2 Pb1 126.891(11) 121_655 30 ?
O1 W2 Pb1 53.109(11) 25 30 ?
Pb1 W2 Pb1 180.0 126_655 30 ?
O1 W2 Pb1 126.891(11) 177_556 74 ?
O1 W2 Pb1 53.109(11) 81 74 ?
O1 W2 Pb1 126.891(11) 149_565 74 ?
O1 W2 Pb1 53.109(11) 53 74 ?
O1 W2 Pb1 121.90(2) 121_655 74 ?
O1 W2 Pb1 58.10(2) 25 74 ?
Pb1 W2 Pb1 174.18(4) 126_655 74 ?
Pb1 W2 Pb1 5.82(4) 30 74 ?
O1 W2 Pb1 58.10(2) 177_556 154_565 ?
O1 W2 Pb1 121.90(2) 81 154_565 ?
O1 W2 Pb1 53.109(11) 149_565 154_565 ?
O1 W2 Pb1 126.891(11) 53 154_565 ?
O1 W2 Pb1 53.109(11) 121_655 154_565 ?
O1 W2 Pb1 126.891(11) 25 154_565 ?
Pb1 W2 Pb1 5.82(4) 126_655 154_565 ?
Pb1 W2 Pb1 174.18(4) 30 154_565 ?
Pb1 W2 Pb1 174.18(4) 74 154_565 ?
O1 W2 Pb1 121.90(2) 177_556 58 ?
O1 W2 Pb1 58.10(2) 81 58 ?
O1 W2 Pb1 126.891(11) 149_565 58 ?
O1 W2 Pb1 53.109(11) 53 58 ?
O1 W2 Pb1 126.891(11) 121_655 58 ?
O1 W2 Pb1 53.109(11) 25 58 ?
Pb1 W2 Pb1 174.18(4) 126_655 58 ?
Pb1 W2 Pb1 5.82(4) 30 58 ?
Pb1 W2 Pb1 5.82(4) 74 58 ?
Pb1 W2 Pb1 180.0 154_565 58 ?
O1 W2 Pb1 53.109(11) 177_556 170_556 ?
O1 W2 Pb1 126.891(11) 81 170_556 ?
O1 W2 Pb1 53.109(11) 149_565 170_556 ?
O1 W2 Pb1 126.891(11) 53 170_556 ?

O1 W2 Pb1 58.10(2) 121_655 170_556 ?
O1 W2 Pb1 121.90(2) 25 170_556 ?
Pb1 W2 Pb1 5.82(4) 126_655 170_556 ?
Pb1 W2 Pb1 174.18(4) 30 170_556 ?
Pb1 W2 Pb1 180.0 74 170_556 ?
Pb1 W2 Pb1 5.82(4) 154_565 170_556 ?
Pb1 W2 Pb1 174.18(4) 58 170_556 ?
O1 Fe2 O1 180.0 177_556 81 ?
O1 Fe2 O1 90.0 177_556 149_565 ?
O1 Fe2 O1 90.0 81 149_565 ?
O1 Fe2 O1 90.0 177_556 53 ?
O1 Fe2 O1 90.0 81 53 ?
O1 Fe2 O1 180.0 149_565 53 ?
O1 Fe2 O1 90.0 177_556 121_655 ?
O1 Fe2 O1 90.0 81 121_655 ?
O1 Fe2 O1 90.000(1) 149_565 121_655 ?
O1 Fe2 O1 90.0 53 121_655 ?
O1 Fe2 O1 90.0 177_556 25 ?
O1 Fe2 O1 90.0 81 25 ?
O1 Fe2 O1 90.0 149_565 25 ?
O1 Fe2 O1 90.000(1) 53 25 ?
O1 Fe2 O1 180.0 121_655 25 ?
O1 Fe2 Pb1 53.109(11) 177_556 126_655 ?
O1 Fe2 Pb1 126.891(11) 81 126_655 ?
O1 Fe2 Pb1 58.10(2) 149_565 126_655 ?
O1 Fe2 Pb1 121.90(2) 53 126_655 ?
O1 Fe2 Pb1 53.109(11) 121_655 126_655 ?
O1 Fe2 Pb1 126.891(11) 25 126_655 ?
O1 Fe2 Pb1 126.891(11) 177_556 30 ?
O1 Fe2 Pb1 53.109(11) 81 30 ?
O1 Fe2 Pb1 121.90(2) 149_565 30 ?
O1 Fe2 Pb1 58.10(2) 53 30 ?
O1 Fe2 Pb1 126.891(11) 121_655 30 ?
O1 Fe2 Pb1 53.109(11) 25 30 ?
Pb1 Fe2 Pb1 180.0 126_655 30 ?
O1 Fe2 Pb1 126.891(11) 177_556 74 ?
O1 Fe2 Pb1 53.109(11) 81 74 ?
O1 Fe2 Pb1 126.891(11) 149_565 74 ?
O1 Fe2 Pb1 53.109(11) 53 74 ?
O1 Fe2 Pb1 121.90(2) 121_655 74 ?
O1 Fe2 Pb1 58.10(2) 25 74 ?
Pb1 Fe2 Pb1 174.18(4) 126_655 74 ?
Pb1 Fe2 Pb1 5.82(4) 30 74 ?
O1 Fe2 Pb1 58.10(2) 177_556 154_565 ?
O1 Fe2 Pb1 121.90(2) 81 154_565 ?
O1 Fe2 Pb1 53.109(11) 149_565 154_565 ?
O1 Fe2 Pb1 126.891(11) 53 154_565 ?
O1 Fe2 Pb1 53.109(11) 121_655 154_565 ?
O1 Fe2 Pb1 126.891(11) 25 154_565 ?
Pb1 Fe2 Pb1 5.82(4) 126_655 154_565 ?
Pb1 Fe2 Pb1 174.18(4) 30 154_565 ?
Pb1 Fe2 Pb1 174.18(4) 74 154_565 ?
O1 Fe2 Pb1 121.90(2) 177_556 58 ?
O1 Fe2 Pb1 58.10(2) 81 58 ?

O1 Fe2 Pb1 126.891(11) 149_565 58 ?
O1 Fe2 Pb1 53.109(11) 53 58 ?
O1 Fe2 Pb1 126.891(11) 121_655 58 ?
O1 Fe2 Pb1 53.109(11) 25 58 ?
Pb1 Fe2 Pb1 174.18(4) 126_655 58 ?
Pb1 Fe2 Pb1 5.82(4) 30 58 ?
Pb1 Fe2 Pb1 5.82(4) 74 58 ?
Pb1 Fe2 Pb1 180.0 154_565 58 ?
O1 Fe2 Pb1 53.109(11) 177_556 170_556 ?
O1 Fe2 Pb1 126.891(11) 81 170_556 ?
O1 Fe2 Pb1 53.109(11) 149_565 170_556 ?
O1 Fe2 Pb1 126.891(11) 53 170_556 ?
O1 Fe2 Pb1 58.10(2) 121_655 170_556 ?
O1 Fe2 Pb1 121.90(2) 25 170_556 ?
Pb1 Fe2 Pb1 5.82(4) 126_655 170_556 ?
Pb1 Fe2 Pb1 174.18(4) 30 170_556 ?
Pb1 Fe2 Pb1 180.0 74 170_556 ?
Pb1 Fe2 Pb1 5.82(4) 154_565 170_556 ?
Pb1 Fe2 Pb1 174.18(4) 58 170_556 ?
Fe2 O1 W2 0.0 25_544 25_544 ?
Fe2 O1 W1 180.0 25_544 . ?
W2 O1 W1 180.0 25_544 . ?
Fe2 O1 Fe1 180.0 25_544 . ?
W2 O1 Fe1 180.0 25_544 . ?
W1 O1 Fe1 0.0 . . ?
Fe2 O1 Pb1 90.03(4) 25_544 5 ?
W2 O1 Pb1 90.03(4) 25_544 5 ?
W1 O1 Pb1 89.97(4) . 5 ?
Fe1 O1 Pb1 89.97(4) . 5 ?
Fe2 O1 Pb1 90.03(4) 25_544 6 ?
W2 O1 Pb1 90.03(4) 25_544 6 ?
W1 O1 Pb1 89.97(4) . 6 ?
Fe1 O1 Pb1 89.97(4) . 6 ?
Pb1 O1 Pb1 179.94(9) 5 6 ?
Fe2 O1 Pb1 90.03(4) 25_544 82_545 ?
W2 O1 Pb1 90.03(4) 25_544 82_545 ?
W1 O1 Pb1 89.97(4) . 82_545 ?
Fe1 O1 Pb1 89.97(4) . 82_545 ?
Pb1 O1 Pb1 172.72(5) 5 82_545 ?
Pb1 O1 Pb1 7.28(5) 6 82_545 ?
Fe2 O1 Pb1 90.03(4) 25_544 9 ?
W2 O1 Pb1 90.03(4) 25_544 9 ?
W1 O1 Pb1 89.97(4) . 9 ?
Fe1 O1 Pb1 89.97(4) . 9 ?
Pb1 O1 Pb1 7.28(5) 5 9 ?
Pb1 O1 Pb1 172.72(5) 6 9 ?
Pb1 O1 Pb1 179.94(9) 82_545 9 ?
Fe2 O1 Pb1 90.03(4) 25_544 150_554 ?
W2 O1 Pb1 90.03(4) 25_544 150_554 ?
W1 O1 Pb1 89.97(4) . 150_554 ?
Fe1 O1 Pb1 89.97(4) . 150_554 ?
Pb1 O1 Pb1 97.28(5) 5 150_554 ?
Pb1 O1 Pb1 82.72(5) 6 150_554 ?
Pb1 O1 Pb1 90.0 82_545 150_554 ?

```
Pb1 O1 Pb1 90.0 9 150_554 ?
Fe2 O1 Pb1 90.03(4) 25_544 177 ?
W2 O1 Pb1 90.03(4) 25_544 177 ?
W1 O1 Pb1 89.97(4) . 177 ?
Fe1 O1 Pb1 89.97(4) . 177 ?
Pb1 O1 Pb1 90.0 5 177 ?
Pb1 O1 Pb1 90.0 6 177 ?
Pb1 O1 Pb1 97.28(5) 82_545 177 ?
Pb1 O1 Pb1 82.72(5) 9 177 ?
Pb1 O1 Pb1 7.28(5) 150_554 177 ?
Fe2 O1 Pb1 90.03(4) 25_544 106 ?
W2 O1 Pb1 90.03(4) 25_544 106 ?
W1 O1 Pb1 89.97(4) . 106 ?
Fe1 O1 Pb1 89.97(4) . 106 ?
Pb1 O1 Pb1 90.0 5 106 ?
Pb1 O1 Pb1 90.0 6 106 ?
Pb1 O1 Pb1 82.72(5) 82_545 106 ?
Pb1 O1 Pb1 97.28(5) 9 106 ?
Pb1 O1 Pb1 172.72(5) 150_554 106 ?
Pb1 O1 Pb1 179.94(9) 177 106 ?
Fe2 O1 Pb1 90.03(4) 25_544 149 ?
W2 O1 Pb1 90.03(4) 25_544 149 ?
W1 O1 Pb1 89.97(4) . 149 ?
Fe1 O1 Pb1 89.97(4) . 149 ?
Pb1 O1 Pb1 82.72(5) 5 149 ?
Pb1 O1 Pb1 97.28(5) 6 149 ?
Pb1 O1 Pb1 90.0 82_545 149 ?
Pb1 O1 Pb1 90.0 9 149 ?
Pb1 O1 Pb1 179.94(9) 150_554 149 ?
Pb1 O1 Pb1 172.72(5) 177 149 ?
Pb1 O1 Pb1 7.28(5) 106 149 ?

_refine_diff_density_max    1.402
_refine_diff_density_min   -1.639
_refine_diff_density_rms    0.261
```

```
data_PbFeWO_280
# start Validation Reply Form

_vrf_PLAT029_PbFeWO_280
;
PROBLEM: _diffrn_measured_fraction_theta_full value Low . 0.950
Why?
RESPONSE: This problem is related to the feature of the PILATUS3 X
CdTe 1M
detector, which has a mask from non-working areas. To predict in
advance
what the number of reflections will be under this mask is
difficult until
the completion of the data set.
;

# end Validation Reply Form

_audit_creation_method            'SHELXL-2014/7'
_chemical_name_systematic         ?
_chemical_name_common             ?
_chemical_melting_point           ?
_chemical_formula_moiety          ?
_chemical_formula_sum
 'Fe0.67 O3 Pb W0.33'
_chemical_formula_weight          353.60

loop_
 _atom_type_symbol
 _atom_type_description
 _atom_type_scat_dispersion_real
 _atom_type_scat_dispersion_imag
 _atom_type_scat_source
 'O'  'O'   0.0017   0.0018
 'International Tables Vol C Tables 4.2.6.8 and 6.1.1.4'
 'Fe'  'Fe'   0.1986   0.3065
 'International Tables Vol C Tables 4.2.6.8 and 6.1.1.4'
 'W'  'W'  -0.4869   2.7805
 'International Tables Vol C Tables 4.2.6.8 and 6.1.1.4'
 'Pb'  'Pb'  -0.5384   4.2194
 'International Tables Vol C Tables 4.2.6.8 and 6.1.1.4'

_space_group_crystal_system       cubic
_space_group_IT_number            225
_space_group_name_H-M_alt         'F m -3 m'
_space_group_name_Hall            '-F 4 2 3'

_shelx_space_group_comment
;
The symmetry employed for this shelxl refinement is uniquely
defined
by the following loop, which should always be used as a source of
symmetry information in preference to the above space-group names.
```

```
They are only intended as comments.
;

loop_
 _space_group_symop_operation_xyz
 'x, y, z'
 '-x, -y, z'
 '-x, y, -z'
 'x, -y, -z'
 'z, x, y'
 'z, -x, -y'
 '-z, -x, y'
 '-z, x, -y'
 'y, z, x'
 '-y, z, -x'
 'y, -z, -x'
 '-y, -z, x'
 'y, x, -z'
 '-y, -x, -z'
 'y, -x, z'
 '-y, x, z'
 'x, z, -y'
 '-x, z, y'
 '-x, -z, -y'
 'x, -z, y'
 'z, y, -x'
 'z, -y, x'
 '-z, y, x'
 '-z, -y, -x'
 'x, y+1/2, z+1/2'
 '-x, -y+1/2, z+1/2'
 '-x, y+1/2, -z+1/2'
 'x, -y+1/2, -z+1/2'
 'z, x+1/2, y+1/2'
 'z, -x+1/2, -y+1/2'
 '-z, -x+1/2, y+1/2'
 '-z, x+1/2, -y+1/2'
 'y, z+1/2, x+1/2'
 '-y, z+1/2, -x+1/2'
 'y, -z+1/2, -x+1/2'
 '-y, -z+1/2, x+1/2'
 'y, x+1/2, -z+1/2'
 '-y, -x+1/2, -z+1/2'
 'y, -x+1/2, z+1/2'
 '-y, x+1/2, z+1/2'
 'x, z+1/2, -y+1/2'
 '-x, z+1/2, y+1/2'
 '-x, -z+1/2, -y+1/2'
 'x, -z+1/2, y+1/2'
 'z, y+1/2, -x+1/2'
 'z, -y+1/2, x+1/2'
 '-z, y+1/2, x+1/2'
 '-z, -y+1/2, -x+1/2'
 'x+1/2, y, z+1/2'
```

```
'-x+1/2, -y, z+1/2'
'-x+1/2, y, -z+1/2'
'x+1/2, -y, -z+1/2'
'z+1/2, x, y+1/2'
'z+1/2, -x, -y+1/2'
'-z+1/2, -x, y+1/2'
'-z+1/2, x, -y+1/2'
'y+1/2, z, x+1/2'
'-y+1/2, z, -x+1/2'
'y+1/2, -z, -x+1/2'
'-y+1/2, -z, x+1/2'
'y+1/2, x, -z+1/2'
'-y+1/2, -x, -z+1/2'
'y+1/2, -x, z+1/2'
'-y+1/2, x, z+1/2'
'x+1/2, z, -y+1/2'
'-x+1/2, z, y+1/2'
'-x+1/2, -z, -y+1/2'
'x+1/2, -z, y+1/2'
'z+1/2, y, -x+1/2'
'z+1/2, -y, x+1/2'
'-z+1/2, y, x+1/2'
'-z+1/2, -y, -x+1/2'
'x+1/2, y+1/2, z'
'-x+1/2, -y+1/2, z'
'-x+1/2, y+1/2, -z'
'x+1/2, -y+1/2, -z'
'z+1/2, x+1/2, y'
'z+1/2, -x+1/2, -y'
'-z+1/2, -x+1/2, y'
'-z+1/2, x+1/2, -y'
'y+1/2, z+1/2, x'
'-y+1/2, z+1/2, -x'
'y+1/2, -z+1/2, -x'
'-y+1/2, -z+1/2, x'
'y+1/2, x+1/2, -z'
'-y+1/2, -x+1/2, -z'
'y+1/2, -x+1/2, z'
'-y+1/2, x+1/2, z'
'x+1/2, z+1/2, -y'
'-x+1/2, z+1/2, y'
'-x+1/2, -z+1/2, -y'
'x+1/2, -z+1/2, y'
'z+1/2, y+1/2, -x'
'z+1/2, -y+1/2, x'
'-z+1/2, y+1/2, x'
'-z+1/2, -y+1/2, -x'
'-x, -y, -z'
'x, y, -z'
'x, -y, z'
'-x, y, z'
'-z, -x, -y'
'-z, x, y'
'z, x, -y'
```

```
'z, -x, y'
'-y, -z, -x'
'y, -z, x'
'-y, z, x'
'y, z, -x'
'-y, -x, z'
'y, x, z'
'-y, x, -z'
'y, -x, -z'
'-x, -z, y'
'x, -z, -y'
'x, z, y'
'-x, z, -y'
'-z, -y, x'
'-z, y, -x'
'z, -y, -x'
'z, y, x'
'-x, -y+1/2, -z+1/2'
'x, y+1/2, -z+1/2'
'x, -y+1/2, z+1/2'
'-x, y+1/2, z+1/2'
'-z, -x+1/2, -y+1/2'
'-z, x+1/2, y+1/2'
'z, x+1/2, -y+1/2'
'z, -x+1/2, y+1/2'
'-y, -z+1/2, -x+1/2'
'y, -z+1/2, x+1/2'
'-y, z+1/2, x+1/2'
'y, z+1/2, -x+1/2'
'-y, -x+1/2, z+1/2'
'y, x+1/2, z+1/2'
'-y, x+1/2, -z+1/2'
'y, -x+1/2, -z+1/2'
'-x, -z+1/2, y+1/2'
'x, -z+1/2, -y+1/2'
'x, z+1/2, y+1/2'
'-x, z+1/2, -y+1/2'
'-z, -y+1/2, x+1/2'
'-z, y+1/2, -x+1/2'
'z, -y+1/2, -x+1/2'
'z, y+1/2, x+1/2'
'-x+1/2, -y, -z+1/2'
'x+1/2, y, -z+1/2'
'x+1/2, -y, z+1/2'
'-x+1/2, y, z+1/2'
'-z+1/2, -x, -y+1/2'
'-z+1/2, x, y+1/2'
'z+1/2, x, -y+1/2'
'z+1/2, -x, y+1/2'
'-y+1/2, -z, -x+1/2'
'y+1/2, -z, x+1/2'
'-y+1/2, z, x+1/2'
'y+1/2, z, -x+1/2'
'-y+1/2, -x, z+1/2'
```

```
 'y+1/2, x, z+1/2'
 '-y+1/2, x, -z+1/2'
 'y+1/2, -x, -z+1/2'
 '-x+1/2, -z, y+1/2'
 'x+1/2, -z, -y+1/2'
 'x+1/2, z, y+1/2'
 '-x+1/2, z, -y+1/2'
 '-z+1/2, -y, x+1/2'
 '-z+1/2, y, -x+1/2'
 'z+1/2, -y, -x+1/2'
 'z+1/2, y, x+1/2'
 '-x+1/2, -y+1/2, -z'
 'x+1/2, y+1/2, -z'
 'x+1/2, -y+1/2, z'
 '-x+1/2, y+1/2, z'
 '-z+1/2, -x+1/2, -y'
 '-z+1/2, x+1/2, y'
 'z+1/2, x+1/2, -y'
 'z+1/2, -x+1/2, y'
 '-y+1/2, -z+1/2, -x'
 'y+1/2, -z+1/2, x'
 '-y+1/2, z+1/2, x'
 'y+1/2, z+1/2, -x'
 '-y+1/2, -x+1/2, z'
 'y+1/2, x+1/2, z'
 '-y+1/2, x+1/2, -z'
 'y+1/2, -x+1/2, -z'
 '-x+1/2, -z+1/2, y'
 'x+1/2, -z+1/2, -y'
 'x+1/2, z+1/2, y'
 '-x+1/2, z+1/2, -y'
 '-z+1/2, -y+1/2, x'
 '-z+1/2, y+1/2, -x'
 'z+1/2, -y+1/2, -x'
 'z+1/2, y+1/2, x'

_cell_length_a                    7.9582(4)
_cell_length_b                    7.9582(4)
_cell_length_c                    7.9582(4)
_cell_angle_alpha                 90
_cell_angle_beta                  90
_cell_angle_gamma                 90
_cell_volume                      504.02(8)
_cell_formula_units_Z             8
_cell_measurement_temperature     280(2)
_cell_measurement_reflns_used     2652
_cell_measurement_theta_min       2.977
_cell_measurement_theta_max       23.505

_exptl_crystal_description        prizm
_exptl_crystal_colour             dark
_exptl_crystal_density_meas       ?
_exptl_crystal_density_method     ?
_exptl_crystal_density_diffrn     9.320
```

```
_exptl_crystal_F_000              1184
_exptl_transmission_factor_min    ?
_exptl_transmission_factor_max    ?
_exptl_crystal_size_max           0.070
_exptl_crystal_size_mid           0.020
_exptl_crystal_size_min           0.010
_exptl_absorpt_coefficient_mu     20.863
_shelx_estimated_absorpt_T_min    0.323
_shelx_estimated_absorpt_T_max    0.819
_exptl_absorpt_correction_type    multi-scan
_exptl_absorpt_correction_T_min   0.300
_exptl_absorpt_correction_T_max   0.428
_exptl_absorpt_process_details
;
 SADABS-2014/4 - Bruker AXS area detector scaling
 and absorption correction
;
_diffrn_ambient_temperature       280(2)
_diffrn_radiation_wavelength      0.41328
_diffrn_radiation_type            'synchrotron'
_diffrn_source                    'Advanced photon source'
_diffrn_radiation_monochromator   'diamond (111)'
_diffrn_measurement_device_type
;
Huber 3 circle diffractometer with PILATUS3 X CdTe 1M detector
;
_diffrn_measurement_method        '\f-scan'
_diffrn_detector_area_resol_mean  ?
_diffrn_standards_number          0
_diffrn_standards_interval_count  0
_diffrn_standards_interval_time   0
_diffrn_standards_decay_%         0.0
_diffrn_reflns_number             2633
_diffrn_reflns_av_unetI/netI      0.0153
_diffrn_reflns_av_R_equivalents   0.0460
_diffrn_reflns_limit_h_min        -13
_diffrn_reflns_limit_h_max        13
_diffrn_reflns_limit_k_min        -14
_diffrn_reflns_limit_k_max        14
_diffrn_reflns_limit_l_min        -12
_diffrn_reflns_limit_l_max        10
_diffrn_reflns_theta_min          2.578
_diffrn_reflns_theta_max          23.929
_diffrn_reflns_theta_full         14.357
_diffrn_measured_fraction_theta_max   0.934
_diffrn_measured_fraction_theta_full  0.950
_diffrn_reflns_Laue_measured_fraction_max    0.934
_diffrn_reflns_Laue_measured_fraction_full   0.950
_diffrn_reflns_point_group_measured_fraction_max   0.934
_diffrn_reflns_point_group_measured_fraction_full  0.950
_reflns_number_total              128
_reflns_number_gt                 123
_reflns_threshold_expression      'I > 2\s(I)'
_reflns_Friedel_coverage          0.000
```

```
_reflns_Friedel_fraction_max      .
_reflns_Friedel_fraction_full     .

_reflns_special_details
;
 Reflections were merged by SHELXL according to the crystal
 class for the calculation of statistics and refinement.

 _reflns_Friedel_fraction is defined as the number of unique
 Friedel pairs measured divided by the number that would be
 possible theoretically, ignoring centric projections and
 systematic absences.
;

_computing_data_collection        ?
_computing_cell_refinement        ?
_computing_data_reduction         ?
_computing_structure_solution     ?
_computing_structure_refinement 'SHELXL-2014/7 (Sheldrick, 2014)'
_computing_molecular_graphics     ?
_computing_publication_material   ?
_refine_special_details           ?
_refine_ls_structure_factor_coef  Fsqd
_refine_ls_matrix_type            full
_refine_ls_weighting_scheme       calc
_refine_ls_weighting_details
'w=1/[\s^2^(Fo^2^)+(0.0165P)^2^+7.4852P] where P=(Fo^2^+2Fc^2^)/3'
_atom_sites_solution_primary      ?
_atom_sites_solution_secondary    ?
_atom_sites_solution_hydrogens    .
_refine_ls_hydrogen_treatment     undef
_refine_ls_extinction_method      'SHELXL-2014/7 (Sheldrick 2014'
_refine_ls_extinction_coef        0.62(5)
_refine_ls_extinction_expression
 'Fc^*^=kFc[1+0.001xFc^2^\l^3^/sin(2\q)]^-1/4^'
_refine_ls_number_reflns          128
_refine_ls_number_parameters      11
_refine_ls_number_restraints      0
_refine_ls_R_factor_all           0.0188
_refine_ls_R_factor_gt            0.0186
_refine_ls_wR_factor_ref          0.0577
_refine_ls_wR_factor_gt           0.0577
_refine_ls_goodness_of_fit_ref    1.290
_refine_ls_restrained_S_all       1.290
_refine_ls_shift/su_max           0.000
_refine_ls_shift/su_mean          0.000

loop_
 _atom_site_label
 _atom_site_type_symbol
 _atom_site_fract_x
 _atom_site_fract_y
 _atom_site_fract_z
 _atom_site_U_iso_or_equiv
```

```
 _atom_site_adp_type
 _atom_site_occupancy
 _atom_site_site_symmetry_order
 _atom_site_calc_flag
 _atom_site_refinement_flags_posn
 _atom_site_refinement_flags_adp
 _atom_site_refinement_flags_occupancy
 _atom_site_disorder_assembly
 _atom_site_disorder_group
Pb1 Pb 0.21978(17) 0.2500 0.2500 0.0184(5) Uani 0.1667 4 d S T P .
.
W1 W 0.0000 0.0000 0.0000 0.0068(3) Uani 0.3072 48 d S T P . .
Fe1 Fe 0.0000 0.0000 0.0000 0.0068(3) Uani 0.6926 48 d S T P . .
W2 W 0.5000 0.5000 0.5000 0.0066(3) Uani 0.359 48 d S T P . .
Fe2 Fe 0.5000 0.5000 0.5000 0.0066(3) Uani 0.6408 48 d S T P . .
O1 O 0.2502(3) 0.0000 0.0000 0.0143(7) Uani 1 8 d S T P . .

loop_
 _atom_site_aniso_label
 _atom_site_aniso_U_11
 _atom_site_aniso_U_22
 _atom_site_aniso_U_33
 _atom_site_aniso_U_23
 _atom_site_aniso_U_13
 _atom_site_aniso_U_12
Pb1 0.0111(6) 0.0220(5) 0.0220(5) -0.00093(14) 0.000 0.000
W1 0.0068(3) 0.0068(3) 0.0068(3) 0.000 0.000 0.000
Fe1 0.0068(3) 0.0068(3) 0.0068(3) 0.000 0.000 0.000
W2 0.0066(3) 0.0066(3) 0.0066(3) 0.000 0.000 0.000
Fe2 0.0066(3) 0.0066(3) 0.0066(3) 0.000 0.000 0.000
O1 0.0078(14) 0.0175(11) 0.0175(11) 0.000 0.000 0.000

_geom_special_details
;
 All esds (except the esd in the dihedral angle between two l.s.
planes)
 are estimated using the full covariance matrix.  The cell esds
are taken
 into account individually in the estimation of esds in distances,
angles
 and torsion angles; correlations between esds in cell parameters
are only
 used when they are defined by crystal symmetry.  An approximate
(isotropic)
 treatment of cell esds is used for estimating esds involving l.s.
planes.
;

loop_
 _geom_bond_atom_site_label_1
 _geom_bond_atom_site_label_2
 _geom_bond_distance
 _geom_bond_site_symmetry_2
 _geom_bond_publ_flag
```

```
Pb1 Pb1 0.340(2) 58 ?
Pb1 Pb1 0.340(2) 30 ?
Pb1 Pb1 0.340(2) 9 ?
Pb1 Pb1 0.340(2) 5 ?
Pb1 Pb1 0.481(3) 74 ?
Pb1 O1 2.6490(9) 9 ?
Pb1 O1 2.6490(9) 5 ?
Pb1 O1 2.6490(9) 129 ?
Pb1 O1 2.6490(9) 125 ?
Pb1 W1 3.3130(8) . ?
Pb1 Fe1 3.3130(8) . ?
Pb1 Fe1 3.3130(8) 25 ?
W1 O1 1.991(3) 105 ?
W1 O1 1.991(3) 9 ?
W1 O1 1.991(3) 101 ?
W1 O1 1.991(3) 5 ?
W1 O1 1.991(3) 97 ?
W1 O1 1.991(3) . ?
W1 Pb1 3.3129(8) 97 ?
W1 Pb1 3.3129(8) 105 ?
W1 Pb1 3.3129(8) 9 ?
W1 Pb1 3.3129(8) 101 ?
W1 Pb1 3.3129(8) 5 ?
W1 Pb1 3.3129(8) 25_544 ?
Fe1 O1 1.991(3) 105 ?
Fe1 O1 1.991(3) 9 ?
Fe1 O1 1.991(3) 101 ?
Fe1 O1 1.991(3) 5 ?
Fe1 O1 1.991(3) 97 ?
Fe1 O1 1.991(3) . ?
Fe1 Pb1 3.3129(8) 97 ?
Fe1 Pb1 3.3129(8) 105 ?
Fe1 Pb1 3.3129(8) 9 ?
Fe1 Pb1 3.3129(8) 101 ?
Fe1 Pb1 3.3129(8) 5 ?
Fe1 Pb1 3.3129(8) 25_544 ?
W2 O1 1.988(3) 177_556 ?
W2 O1 1.988(3) 81 ?
W2 O1 1.988(3) 149_565 ?
W2 O1 1.988(3) 53 ?
W2 O1 1.988(3) 121_655 ?
W2 O1 1.988(3) 25 ?
W2 Pb1 3.3129(8) 74 ?
W2 Pb1 3.3129(8) 154_565 ?
W2 Pb1 3.3129(8) 170_556 ?
W2 Pb1 3.3129(8) 58 ?
W2 Pb1 3.3129(8) 126_655 ?
W2 Pb1 3.3129(8) 30 ?
Fe2 O1 1.988(3) 177_556 ?
Fe2 O1 1.988(3) 81 ?
Fe2 O1 1.988(3) 149_565 ?
Fe2 O1 1.988(3) 53 ?
Fe2 O1 1.988(3) 121_655 ?
Fe2 O1 1.988(3) 25 ?
```

```
Fe2 Pb1 3.3129(8) 74 ?
Fe2 Pb1 3.3129(8) 154_565 ?
Fe2 Pb1 3.3129(8) 170_556 ?
Fe2 Pb1 3.3129(8) 58 ?
Fe2 Pb1 3.3129(8) 126_655 ?
Fe2 Pb1 3.3129(8) 30 ?
O1 Fe2 1.988(3) 25_544 ?
O1 W2 1.988(3) 25_544 ?
O1 Pb1 2.6490(9) 5 ?
O1 Pb1 2.6490(9) 6 ?
O1 Pb1 2.6490(9) 82_545 ?
O1 Pb1 2.6490(9) 9 ?
O1 Pb1 2.6490(9) 150_554 ?
O1 Pb1 2.6490(9) 177 ?
O1 Pb1 2.6490(9) 106 ?
O1 Pb1 2.6490(9) 149 ?

loop_
 _geom_angle_atom_site_label_1
 _geom_angle_atom_site_label_2
 _geom_angle_atom_site_label_3
 _geom_angle
 _geom_angle_site_symmetry_1
 _geom_angle_site_symmetry_3
 _geom_angle_publ_flag
Pb1 Pb1 Pb1 60.004(1) 58 30 ?
Pb1 Pb1 Pb1 90.007(1) 58 9 ?
Pb1 Pb1 Pb1 60.004(1) 30 9 ?
Pb1 Pb1 Pb1 60.0 58 5 ?
Pb1 Pb1 Pb1 90.007(1) 30 5 ?
Pb1 Pb1 Pb1 60.0 9 5 ?
Pb1 Pb1 Pb1 45.0 58 74 ?
Pb1 Pb1 Pb1 45.0 30 74 ?
Pb1 Pb1 Pb1 45.0 9 74 ?
Pb1 Pb1 Pb1 45.0 5 74 ?
Pb1 Pb1 O1 117.80(5) 58 9 ?
Pb1 Pb1 O1 176.32(2) 30 9 ?
Pb1 Pb1 O1 117.86(5) 9 9 ?
Pb1 Pb1 O1 86.32(2) 5 9 ?
Pb1 Pb1 O1 131.32(2) 74 9 ?
Pb1 Pb1 O1 176.32(2) 58 5 ?
Pb1 Pb1 O1 117.80(5) 30 5 ?
Pb1 Pb1 O1 86.32(2) 9 5 ?
Pb1 Pb1 O1 117.86(5) 5 5 ?
Pb1 Pb1 O1 131.32(2) 74 5 ?
O1 Pb1 O1 64.22(10) 9 5 ?
Pb1 Pb1 O1 117.86(5) 58 129 ?
Pb1 Pb1 O1 86.32(2) 30 129 ?
Pb1 Pb1 O1 117.80(5) 9 129 ?
Pb1 Pb1 O1 176.32(2) 5 129 ?
Pb1 Pb1 O1 131.32(2) 74 129 ?
O1 Pb1 O1 97.36(5) 9 129 ?
O1 Pb1 O1 64.09(10) 5 129 ?
Pb1 Pb1 O1 86.32(2) 58 125 ?
```

Pb1 Pb1 O1 117.86(5) 30 125 ?
Pb1 Pb1 O1 176.32(2) 9 125 ?
Pb1 Pb1 O1 117.80(5) 5 125 ?
Pb1 Pb1 O1 131.32(2) 74 125 ?
O1 Pb1 O1 64.09(10) 9 125 ?
O1 Pb1 O1 97.36(5) 5 125 ?
O1 Pb1 O1 64.22(10) 129 125 ?
Pb1 Pb1 W1 142.935(12) 58 . ?
Pb1 Pb1 W1 142.935(11) 30 . ?
Pb1 Pb1 W1 87.054(18) 9 . ?
Pb1 Pb1 W1 87.054(18) 5 . ?
Pb1 Pb1 W1 121.87(2) 74 . ?
O1 Pb1 W1 36.95(6) 9 . ?
O1 Pb1 W1 36.95(6) 5 . ?
O1 Pb1 W1 95.86(5) 129 . ?
O1 Pb1 W1 95.86(5) 125 . ?
Pb1 Pb1 Fe1 142.935(12) 58 . ?
Pb1 Pb1 Fe1 142.935(11) 30 . ?
Pb1 Pb1 Fe1 87.054(18) 9 . ?
Pb1 Pb1 Fe1 87.054(18) 5 . ?
Pb1 Pb1 Fe1 121.87(2) 74 . ?
O1 Pb1 Fe1 36.95(6) 9 . ?
O1 Pb1 Fe1 36.95(6) 5 . ?
O1 Pb1 Fe1 95.86(5) 129 . ?
O1 Pb1 Fe1 95.86(5) 125 . ?
W1 Pb1 Fe1 0.0 . . ?
Pb1 Pb1 Fe1 87.054(18) 58 25 ?
Pb1 Pb1 Fe1 87.054(18) 30 25 ?
Pb1 Pb1 Fe1 142.935(11) 9 25 ?
Pb1 Pb1 Fe1 142.935(11) 5 25 ?
Pb1 Pb1 Fe1 121.87(2) 74 25 ?
O1 Pb1 Fe1 95.86(5) 9 25 ?
O1 Pb1 Fe1 95.86(5) 5 25 ?
O1 Pb1 Fe1 36.95(6) 129 25 ?
O1 Pb1 Fe1 36.95(6) 125 25 ?
W1 Pb1 Fe1 116.27(4) . 25 ?
Fe1 Pb1 Fe1 116.27(4) . 25 ?
O1 W1 O1 180.0 105 9 ?
O1 W1 O1 90.0 105 101 ?
O1 W1 O1 90.0 9 101 ?
O1 W1 O1 90.0 105 5 ?
O1 W1 O1 90.0 9 5 ?
O1 W1 O1 180.0 101 5 ?
O1 W1 O1 90.0 105 97 ?
O1 W1 O1 90.0 9 97 ?
O1 W1 O1 90.0 101 97 ?
O1 W1 O1 90.0 5 97 ?
O1 W1 O1 90.0 105 . ?
O1 W1 O1 90.0 9 . ?
O1 W1 O1 90.0 101 . ?
O1 W1 O1 90.0 5 . ?
O1 W1 O1 180.0 97 . ?
O1 W1 Pb1 53.091(10) 105 97 ?
O1 W1 Pb1 126.909(10) 9 97 ?

O1 W1 Pb1 53.091(10) 101 97 ?
O1 W1 Pb1 126.909(10) 5 97 ?
O1 W1 Pb1 58.13(2) 97 97 ?
O1 W1 Pb1 121.87(2) . 97 ?
O1 W1 Pb1 58.13(2) 105 105 ?
O1 W1 Pb1 121.87(2) 9 105 ?
O1 W1 Pb1 53.091(10) 101 105 ?
O1 W1 Pb1 126.909(10) 5 105 ?
O1 W1 Pb1 53.091(10) 97 105 ?
O1 W1 Pb1 126.909(10) . 105 ?
Pb1 W1 Pb1 5.89(4) 97 105 ?
O1 W1 Pb1 121.87(2) 105 9 ?
O1 W1 Pb1 58.13(2) 9 9 ?
O1 W1 Pb1 126.909(10) 101 9 ?
O1 W1 Pb1 53.091(10) 5 9 ?
O1 W1 Pb1 126.909(10) 97 9 ?
O1 W1 Pb1 53.091(10) . 9 ?
Pb1 W1 Pb1 174.11(4) 97 9 ?
Pb1 W1 Pb1 180.0 105 9 ?
O1 W1 Pb1 53.091(10) 105 101 ?
O1 W1 Pb1 126.909(10) 9 101 ?
O1 W1 Pb1 58.13(2) 101 101 ?
O1 W1 Pb1 121.87(2) 5 101 ?
O1 W1 Pb1 53.091(10) 97 101 ?
O1 W1 Pb1 126.909(10) . 101 ?
Pb1 W1 Pb1 5.89(4) 97 101 ?
Pb1 W1 Pb1 5.89(4) 105 101 ?
Pb1 W1 Pb1 174.11(4) 9 101 ?
O1 W1 Pb1 126.909(10) 105 5 ?
O1 W1 Pb1 53.091(10) 9 5 ?
O1 W1 Pb1 121.87(2) 101 5 ?
O1 W1 Pb1 58.13(2) 5 5 ?
O1 W1 Pb1 126.909(10) 97 5 ?
O1 W1 Pb1 53.091(10) . 5 ?
Pb1 W1 Pb1 174.11(4) 97 5 ?
Pb1 W1 Pb1 174.11(4) 105 5 ?
Pb1 W1 Pb1 5.89(4) 9 5 ?
Pb1 W1 Pb1 180.0 101 5 ?
O1 W1 Pb1 53.091(10) 105 25_544 ?
O1 W1 Pb1 126.909(10) 9 25_544 ?
O1 W1 Pb1 53.091(10) 101 25_544 ?
O1 W1 Pb1 126.909(10) 5 25_544 ?
O1 W1 Pb1 121.87(2) 97 25_544 ?
O1 W1 Pb1 58.13(2) . 25_544 ?
Pb1 W1 Pb1 63.73(4) 97 25_544 ?
Pb1 W1 Pb1 68.860(10) 105 25_544 ?
Pb1 W1 Pb1 111.140(10) 9 25_544 ?
Pb1 W1 Pb1 68.860(10) 101 25_544 ?
Pb1 W1 Pb1 111.140(10) 5 25_544 ?
O1 Fe1 O1 180.0 105 9 ?
O1 Fe1 O1 90.0 105 101 ?
O1 Fe1 O1 90.0 9 101 ?
O1 Fe1 O1 90.0 105 5 ?
O1 Fe1 O1 90.0 9 5 ?

O1 Fe1 O1 180.0 101 5 ?
O1 Fe1 O1 90.0 105 97 ?
O1 Fe1 O1 90.0 9 97 ?
O1 Fe1 O1 90.0 101 97 ?
O1 Fe1 O1 90.0 5 97 ?
O1 Fe1 O1 90.0 105 . ?
O1 Fe1 O1 90.0 9 . ?
O1 Fe1 O1 90.0 101 . ?
O1 Fe1 O1 90.0 5 . ?
O1 Fe1 O1 180.0 97 . ?
O1 Fe1 Pb1 53.091(10) 105 97 ?
O1 Fe1 Pb1 126.909(10) 9 97 ?
O1 Fe1 Pb1 53.091(10) 101 97 ?
O1 Fe1 Pb1 126.909(10) 5 97 ?
O1 Fe1 Pb1 58.13(2) 97 97 ?
O1 Fe1 Pb1 121.87(2) . 97 ?
O1 Fe1 Pb1 58.13(2) 105 105 ?
O1 Fe1 Pb1 121.87(2) 9 105 ?
O1 Fe1 Pb1 53.091(10) 101 105 ?
O1 Fe1 Pb1 126.909(10) 5 105 ?
O1 Fe1 Pb1 53.091(10) 97 105 ?
O1 Fe1 Pb1 126.909(10) . 105 ?
Pb1 Fe1 Pb1 5.89(4) 97 105 ?
O1 Fe1 Pb1 121.87(2) 105 9 ?
O1 Fe1 Pb1 58.13(2) 9 9 ?
O1 Fe1 Pb1 126.909(10) 101 9 ?
O1 Fe1 Pb1 53.091(10) 5 9 ?
O1 Fe1 Pb1 126.909(10) 97 9 ?
O1 Fe1 Pb1 53.091(10) . 9 ?
Pb1 Fe1 Pb1 174.11(4) 97 9 ?
Pb1 Fe1 Pb1 180.0 105 9 ?
O1 Fe1 Pb1 53.091(10) 105 101 ?
O1 Fe1 Pb1 126.909(10) 9 101 ?
O1 Fe1 Pb1 58.13(2) 101 101 ?
O1 Fe1 Pb1 121.87(2) 5 101 ?
O1 Fe1 Pb1 53.091(10) 97 101 ?
O1 Fe1 Pb1 126.909(10) . 101 ?
Pb1 Fe1 Pb1 5.89(4) 97 101 ?
Pb1 Fe1 Pb1 5.89(4) 105 101 ?
Pb1 Fe1 Pb1 174.11(4) 9 101 ?
O1 Fe1 Pb1 126.909(10) 105 5 ?
O1 Fe1 Pb1 53.091(10) 9 5 ?
O1 Fe1 Pb1 121.87(2) 101 5 ?
O1 Fe1 Pb1 58.13(2) 5 5 ?
O1 Fe1 Pb1 126.909(10) 97 5 ?
O1 Fe1 Pb1 53.091(10) . 5 ?
Pb1 Fe1 Pb1 174.11(4) 97 5 ?
Pb1 Fe1 Pb1 174.11(4) 105 5 ?
Pb1 Fe1 Pb1 5.89(4) 9 5 ?
Pb1 Fe1 Pb1 180.0 101 5 ?
O1 Fe1 Pb1 53.091(10) 105 25_544 ?
O1 Fe1 Pb1 126.909(10) 9 25_544 ?
O1 Fe1 Pb1 53.091(10) 101 25_544 ?
O1 Fe1 Pb1 126.909(10) 5 25_544 ?

O1 Fe1 Pb1 121.87(2) 97 25_544 ?
O1 Fe1 Pb1 58.13(2) . 25_544 ?
Pb1 Fe1 Pb1 63.73(4) 97 25_544 ?
Pb1 Fe1 Pb1 68.860(10) 105 25_544 ?
Pb1 Fe1 Pb1 111.140(10) 9 25_544 ?
Pb1 Fe1 Pb1 68.860(10) 101 25_544 ?
Pb1 Fe1 Pb1 111.140(10) 5 25_544 ?
O1 W2 O1 180.0 177_556 81 ?
O1 W2 O1 90.0 177_556 149_565 ?
O1 W2 O1 90.0 81 149_565 ?
O1 W2 O1 90.0 177_556 53 ?
O1 W2 O1 90.0 81 53 ?
O1 W2 O1 180.0 149_565 53 ?
O1 W2 O1 90.000(1) 177_556 121_655 ?
O1 W2 O1 90.0 81 121_655 ?
O1 W2 O1 90.000(1) 149_565 121_655 ?
O1 W2 O1 90.0 53 121_655 ?
O1 W2 O1 90.0 177_556 25 ?
O1 W2 O1 90.000(1) 81 25 ?
O1 W2 O1 90.0 149_565 25 ?
O1 W2 O1 90.000(1) 53 25 ?
O1 W2 O1 180.0 121_655 25 ?
O1 W2 Pb1 126.909(10) 177_556 74 ?
O1 W2 Pb1 53.091(10) 81 74 ?
O1 W2 Pb1 126.909(10) 149_565 74 ?
O1 W2 Pb1 53.091(10) 53 74 ?
O1 W2 Pb1 121.87(2) 121_655 74 ?
O1 W2 Pb1 58.13(2) 25 74 ?
O1 W2 Pb1 58.13(2) 177_556 154_565 ?
O1 W2 Pb1 121.87(2) 81 154_565 ?
O1 W2 Pb1 53.091(10) 149_565 154_565 ?
O1 W2 Pb1 126.909(10) 53 154_565 ?
O1 W2 Pb1 53.091(10) 121_655 154_565 ?
O1 W2 Pb1 126.909(10) 25 154_565 ?
Pb1 W2 Pb1 174.11(4) 74 154_565 ?
O1 W2 Pb1 53.091(10) 177_556 170_556 ?
O1 W2 Pb1 126.909(10) 81 170_556 ?
O1 W2 Pb1 53.091(10) 149_565 170_556 ?
O1 W2 Pb1 126.909(10) 53 170_556 ?
O1 W2 Pb1 58.13(2) 121_655 170_556 ?
O1 W2 Pb1 121.87(2) 25 170_556 ?
Pb1 W2 Pb1 180.0 74 170_556 ?
Pb1 W2 Pb1 5.89(4) 154_565 170_556 ?
O1 W2 Pb1 121.87(2) 177_556 58 ?
O1 W2 Pb1 58.13(2) 81 58 ?
O1 W2 Pb1 126.909(10) 149_565 58 ?
O1 W2 Pb1 53.091(10) 53 58 ?
O1 W2 Pb1 126.909(10) 121_655 58 ?
O1 W2 Pb1 53.091(10) 25 58 ?
Pb1 W2 Pb1 5.89(4) 74 58 ?
Pb1 W2 Pb1 180.0 154_565 58 ?
Pb1 W2 Pb1 174.11(4) 170_556 58 ?
O1 W2 Pb1 53.091(10) 177_556 126_655 ?
O1 W2 Pb1 126.909(10) 81 126_655 ?

O1 W2 Pb1 58.13(2) 149_565 126_655 ?
O1 W2 Pb1 121.87(2) 53 126_655 ?
O1 W2 Pb1 53.091(10) 121_655 126_655 ?
O1 W2 Pb1 126.909(10) 25 126_655 ?
Pb1 W2 Pb1 174.11(4) 74 126_655 ?
Pb1 W2 Pb1 5.89(4) 154_565 126_655 ?
Pb1 W2 Pb1 5.89(4) 170_556 126_655 ?
Pb1 W2 Pb1 174.11(4) 58 126_655 ?
O1 W2 Pb1 126.909(10) 177_556 30 ?
O1 W2 Pb1 53.091(10) 81 30 ?
O1 W2 Pb1 121.87(2) 149_565 30 ?
O1 W2 Pb1 58.13(2) 53 30 ?
O1 W2 Pb1 126.909(10) 121_655 30 ?
O1 W2 Pb1 53.091(10) 25 30 ?
Pb1 W2 Pb1 5.89(4) 74 30 ?
Pb1 W2 Pb1 174.11(4) 154_565 30 ?
Pb1 W2 Pb1 174.11(4) 170_556 30 ?
Pb1 W2 Pb1 5.89(4) 58 30 ?
Pb1 W2 Pb1 180.0 126_655 30 ?
O1 Fe2 O1 180.0 177_556 81 ?
O1 Fe2 O1 90.0 177_556 149_565 ?
O1 Fe2 O1 90.0 81 149_565 ?
O1 Fe2 O1 90.0 177_556 53 ?
O1 Fe2 O1 90.0 81 53 ?
O1 Fe2 O1 180.0 149_565 53 ?
O1 Fe2 O1 90.000(1) 177_556 121_655 ?
O1 Fe2 O1 90.0 81 121_655 ?
O1 Fe2 O1 90.000(1) 149_565 121_655 ?
O1 Fe2 O1 90.0 53 121_655 ?
O1 Fe2 O1 90.0 177_556 25 ?
O1 Fe2 O1 90.000(1) 81 25 ?
O1 Fe2 O1 90.0 149_565 25 ?
O1 Fe2 O1 90.000(1) 53 25 ?
O1 Fe2 O1 180.0 121_655 25 ?
O1 Fe2 Pb1 126.909(10) 177_556 74 ?
O1 Fe2 Pb1 53.091(10) 81 74 ?
O1 Fe2 Pb1 126.909(10) 149_565 74 ?
O1 Fe2 Pb1 53.091(10) 53 74 ?
O1 Fe2 Pb1 121.87(2) 121_655 74 ?
O1 Fe2 Pb1 58.13(2) 25 74 ?
O1 Fe2 Pb1 58.13(2) 177_556 154_565 ?
O1 Fe2 Pb1 121.87(2) 81 154_565 ?
O1 Fe2 Pb1 53.091(10) 149_565 154_565 ?
O1 Fe2 Pb1 126.909(10) 53 154_565 ?
O1 Fe2 Pb1 53.091(10) 121_655 154_565 ?
O1 Fe2 Pb1 126.909(10) 25 154_565 ?
Pb1 Fe2 Pb1 174.11(4) 74 154_565 ?
O1 Fe2 Pb1 53.091(10) 177_556 170_556 ?
O1 Fe2 Pb1 126.909(10) 81 170_556 ?
O1 Fe2 Pb1 53.091(10) 149_565 170_556 ?
O1 Fe2 Pb1 126.909(10) 53 170_556 ?
O1 Fe2 Pb1 58.13(2) 121_655 170_556 ?
O1 Fe2 Pb1 121.87(2) 25 170_556 ?
Pb1 Fe2 Pb1 180.0 74 170_556 ?

Pb1 Fe2 Pb1 5.89(4) 154_565 170_556 ?
O1 Fe2 Pb1 121.87(2) 177_556 58 ?
O1 Fe2 Pb1 58.13(2) 81 58 ?
O1 Fe2 Pb1 126.909(10) 149_565 58 ?
O1 Fe2 Pb1 53.091(10) 53 58 ?
O1 Fe2 Pb1 126.909(10) 121_655 58 ?
O1 Fe2 Pb1 53.091(10) 25 58 ?
Pb1 Fe2 Pb1 5.89(4) 74 58 ?
Pb1 Fe2 Pb1 180.0 154_565 58 ?
Pb1 Fe2 Pb1 174.11(4) 170_556 58 ?
O1 Fe2 Pb1 53.091(10) 177_556 126_655 ?
O1 Fe2 Pb1 126.909(10) 81 126_655 ?
O1 Fe2 Pb1 58.13(2) 149_565 126_655 ?
O1 Fe2 Pb1 121.87(2) 53 126_655 ?
O1 Fe2 Pb1 53.091(10) 121_655 126_655 ?
O1 Fe2 Pb1 126.909(10) 25 126_655 ?
Pb1 Fe2 Pb1 174.11(4) 74 126_655 ?
Pb1 Fe2 Pb1 5.89(4) 154_565 126_655 ?
Pb1 Fe2 Pb1 5.89(4) 170_556 126_655 ?
Pb1 Fe2 Pb1 174.11(4) 58 126_655 ?
O1 Fe2 Pb1 126.909(10) 177_556 30 ?
O1 Fe2 Pb1 53.091(10) 81 30 ?
O1 Fe2 Pb1 121.87(2) 149_565 30 ?
O1 Fe2 Pb1 58.13(2) 53 30 ?
O1 Fe2 Pb1 126.909(10) 121_655 30 ?
O1 Fe2 Pb1 53.091(10) 25 30 ?
Pb1 Fe2 Pb1 5.89(4) 74 30 ?
Pb1 Fe2 Pb1 174.11(4) 154_565 30 ?
Pb1 Fe2 Pb1 174.11(4) 170_556 30 ?
Pb1 Fe2 Pb1 5.89(4) 58 30 ?
Pb1 Fe2 Pb1 180.0 126_655 30 ?
Fe2 O1 W2 0.0 25_544 25_544 ?
Fe2 O1 W1 180.0 25_544 . ?
W2 O1 W1 180.0 25_544 . ?
Fe2 O1 Fe1 180.0 25_544 . ?
W2 O1 Fe1 180.0 25_544 . ?
W1 O1 Fe1 0.0 . . ?
Fe2 O1 Pb1 90.04(6) 25_544 5 ?
W2 O1 Pb1 90.04(6) 25_544 5 ?
W1 O1 Pb1 89.96(6) . 5 ?
Fe1 O1 Pb1 89.96(6) . 5 ?
Fe2 O1 Pb1 90.04(6) 25_544 6 ?
W2 O1 Pb1 90.04(6) 25_544 6 ?
W1 O1 Pb1 89.96(6) . 6 ?
Fe1 O1 Pb1 89.96(6) . 6 ?
Pb1 O1 Pb1 179.92(12) 5 6 ?
Fe2 O1 Pb1 90.04(6) 25_544 82_545 ?
W2 O1 Pb1 90.04(6) 25_544 82_545 ?
W1 O1 Pb1 89.96(6) . 82_545 ?
Fe1 O1 Pb1 89.96(6) . 82_545 ?
Pb1 O1 Pb1 172.64(5) 5 82_545 ?
Pb1 O1 Pb1 7.36(5) 6 82_545 ?
Fe2 O1 Pb1 90.04(6) 25_544 9 ?
W2 O1 Pb1 90.04(6) 25_544 9 ?

```
W1 O1 Pb1 89.96(6) . 9 ?
Fe1 O1 Pb1 89.96(6) . 9 ?
Pb1 O1 Pb1 7.36(5) 5 9 ?
Pb1 O1 Pb1 172.64(5) 6 9 ?
Pb1 O1 Pb1 179.92(12) 82_545 9 ?
Fe2 O1 Pb1 90.04(6) 25_544 150_554 ?
W2 O1 Pb1 90.04(6) 25_544 150_554 ?
W1 O1 Pb1 89.96(6) . 150_554 ?
Fe1 O1 Pb1 89.96(6) . 150_554 ?
Pb1 O1 Pb1 97.36(5) 5 150_554 ?
Pb1 O1 Pb1 82.64(5) 6 150_554 ?
Pb1 O1 Pb1 90.0 82_545 150_554 ?
Pb1 O1 Pb1 90.0 9 150_554 ?
Fe2 O1 Pb1 90.04(6) 25_544 177 ?
W2 O1 Pb1 90.04(6) 25_544 177 ?
W1 O1 Pb1 89.96(6) . 177 ?
Fe1 O1 Pb1 89.96(6) . 177 ?
Pb1 O1 Pb1 90.0 5 177 ?
Pb1 O1 Pb1 90.0 6 177 ?
Pb1 O1 Pb1 97.36(5) 82_545 177 ?
Pb1 O1 Pb1 82.64(5) 9 177 ?
Pb1 O1 Pb1 7.36(5) 150_554 177 ?
Fe2 O1 Pb1 90.04(6) 25_544 106 ?
W2 O1 Pb1 90.04(6) 25_544 106 ?
W1 O1 Pb1 89.96(6) . 106 ?
Fe1 O1 Pb1 89.96(6) . 106 ?
Pb1 O1 Pb1 90.0 5 106 ?
Pb1 O1 Pb1 90.0 6 106 ?
Pb1 O1 Pb1 82.64(5) 82_545 106 ?
Pb1 O1 Pb1 97.36(5) 9 106 ?
Pb1 O1 Pb1 172.64(5) 150_554 106 ?
Pb1 O1 Pb1 179.92(12) 177 106 ?
Fe2 O1 Pb1 90.04(6) 25_544 149 ?
W2 O1 Pb1 90.04(6) 25_544 149 ?
W1 O1 Pb1 89.96(6) . 149 ?
Fe1 O1 Pb1 89.96(6) . 149 ?
Pb1 O1 Pb1 82.64(5) 5 149 ?
Pb1 O1 Pb1 97.36(5) 6 149 ?
Pb1 O1 Pb1 90.0 82_545 149 ?
Pb1 O1 Pb1 90.0 9 149 ?
Pb1 O1 Pb1 179.92(12) 150_554 149 ?
Pb1 O1 Pb1 172.64(5) 177 149 ?
Pb1 O1 Pb1 7.36(5) 106 149 ?

_refine_diff_density_max    1.672
_refine_diff_density_min   -0.982
_refine_diff_density_rms    0.206
```

```
data_PbFeWO_400
# start Validation Reply Form

_vrf_PLAT029_PbFeWO_400
;
PROBLEM: _diffrn_measured_fraction_theta_full value Low . 0.950
Why?
RESPONSE: This problem is related to the feature of the PILATUS3 X
CdTe 1M
detector, which has a mask from non-working areas. To predict in
advance
what the number of reflections will be under this mask is
difficult until
the completion of the data set.
;

# end Validation Reply Form

_audit_creation_method            'SHELXL-2014/7'
_chemical_name_systematic         ?
_chemical_name_common             ?
_chemical_melting_point           ?
_chemical_formula_moiety          ?
_chemical_formula_sum
 'Fe0.67 O3 Pb W0.33'
_chemical_formula_weight          353.28

loop_
 _atom_type_symbol
 _atom_type_description
 _atom_type_scat_dispersion_real
 _atom_type_scat_dispersion_imag
 _atom_type_scat_source
 'O'  'O'   0.0017   0.0018
 'International Tables Vol C Tables 4.2.6.8 and 6.1.1.4'
 'Fe'  'Fe'   0.1986   0.3065
 'International Tables Vol C Tables 4.2.6.8 and 6.1.1.4'
 'W'  'W'  -0.4869   2.7805
 'International Tables Vol C Tables 4.2.6.8 and 6.1.1.4'
 'Pb'  'Pb'  -0.5384   4.2194
 'International Tables Vol C Tables 4.2.6.8 and 6.1.1.4'

_space_group_crystal_system       cubic
_space_group_IT_number            225
_space_group_name_H-M_alt         'F m -3 m'
_space_group_name_Hall            '-F 4 2 3'

_shelx_space_group_comment
;
The symmetry employed for this shelxl refinement is uniquely
defined
by the following loop, which should always be used as a source of
symmetry information in preference to the above space-group names.
```

```
They are only intended as comments.
;

loop_
 _space_group_symop_operation_xyz
 'x, y, z'
 '-x, -y, z'
 '-x, y, -z'
 'x, -y, -z'
 'z, x, y'
 'z, -x, -y'
 '-z, -x, y'
 '-z, x, -y'
 'y, z, x'
 '-y, z, -x'
 'y, -z, -x'
 '-y, -z, x'
 'y, x, -z'
 '-y, -x, -z'
 'y, -x, z'
 '-y, x, z'
 'x, z, -y'
 '-x, z, y'
 '-x, -z, -y'
 'x, -z, y'
 'z, y, -x'
 'z, -y, x'
 '-z, y, x'
 '-z, -y, -x'
 'x, y+1/2, z+1/2'
 '-x, -y+1/2, z+1/2'
 '-x, y+1/2, -z+1/2'
 'x, -y+1/2, -z+1/2'
 'z, x+1/2, y+1/2'
 'z, -x+1/2, -y+1/2'
 '-z, -x+1/2, y+1/2'
 '-z, x+1/2, -y+1/2'
 'y, z+1/2, x+1/2'
 '-y, z+1/2, -x+1/2'
 'y, -z+1/2, -x+1/2'
 '-y, -z+1/2, x+1/2'
 'y, x+1/2, -z+1/2'
 '-y, -x+1/2, -z+1/2'
 'y, -x+1/2, z+1/2'
 '-y, x+1/2, z+1/2'
 'x, z+1/2, -y+1/2'
 '-x, z+1/2, y+1/2'
 '-x, -z+1/2, -y+1/2'
 'x, -z+1/2, y+1/2'
 'z, y+1/2, -x+1/2'
 'z, -y+1/2, x+1/2'
 '-z, y+1/2, x+1/2'
 '-z, -y+1/2, -x+1/2'
 'x+1/2, y, z+1/2'
```

```
'-x+1/2, -y, z+1/2'
'-x+1/2, y, -z+1/2'
'x+1/2, -y, -z+1/2'
'z+1/2, x, y+1/2'
'z+1/2, -x, -y+1/2'
'-z+1/2, -x, y+1/2'
'-z+1/2, x, -y+1/2'
'y+1/2, z, x+1/2'
'-y+1/2, z, -x+1/2'
'y+1/2, -z, -x+1/2'
'-y+1/2, -z, x+1/2'
'y+1/2, x, -z+1/2'
'-y+1/2, -x, -z+1/2'
'y+1/2, -x, z+1/2'
'-y+1/2, x, z+1/2'
'x+1/2, z, -y+1/2'
'-x+1/2, z, y+1/2'
'-x+1/2, -z, -y+1/2'
'x+1/2, -z, y+1/2'
'z+1/2, y, -x+1/2'
'z+1/2, -y, x+1/2'
'-z+1/2, y, x+1/2'
'-z+1/2, -y, -x+1/2'
'x+1/2, y+1/2, z'
'-x+1/2, -y+1/2, z'
'-x+1/2, y+1/2, -z'
'x+1/2, -y+1/2, -z'
'z+1/2, x+1/2, y'
'z+1/2, -x+1/2, -y'
'-z+1/2, -x+1/2, y'
'-z+1/2, x+1/2, -y'
'y+1/2, z+1/2, x'
'-y+1/2, z+1/2, -x'
'y+1/2, -z+1/2, -x'
'-y+1/2, -z+1/2, x'
'y+1/2, x+1/2, -z'
'-y+1/2, -x+1/2, -z'
'y+1/2, -x+1/2, z'
'-y+1/2, x+1/2, z'
'x+1/2, z+1/2, -y'
'-x+1/2, z+1/2, y'
'-x+1/2, -z+1/2, -y'
'x+1/2, -z+1/2, y'
'z+1/2, y+1/2, -x'
'z+1/2, -y+1/2, x'
'-z+1/2, y+1/2, x'
'-z+1/2, -y+1/2, -x'
'-x, -y, -z'
'x, y, -z'
'x, -y, z'
'-x, y, z'
'-z, -x, -y'
'-z, x, y'
'z, x, -y'
```

```
'z, -x, y'
'-y, -z, -x'
'y, -z, x'
'-y, z, x'
'y, z, -x'
'-y, -x, z'
'y, x, z'
'-y, x, -z'
'y, -x, -z'
'-x, -z, y'
'x, -z, -y'
'x, z, y'
'-x, z, -y'
'-z, -y, x'
'-z, y, -x'
'z, -y, -x'
'z, y, x'
'-x, -y+1/2, -z+1/2'
'x, y+1/2, -z+1/2'
'x, -y+1/2, z+1/2'
'-x, y+1/2, z+1/2'
'-z, -x+1/2, -y+1/2'
'-z, x+1/2, y+1/2'
'z, x+1/2, -y+1/2'
'z, -x+1/2, y+1/2'
'-y, -z+1/2, -x+1/2'
'y, -z+1/2, x+1/2'
'-y, z+1/2, x+1/2'
'y, z+1/2, -x+1/2'
'-y, -x+1/2, z+1/2'
'y, x+1/2, z+1/2'
'-y, x+1/2, -z+1/2'
'y, -x+1/2, -z+1/2'
'-x, -z+1/2, y+1/2'
'x, -z+1/2, -y+1/2'
'x, z+1/2, y+1/2'
'-x, z+1/2, -y+1/2'
'-z, -y+1/2, x+1/2'
'-z, y+1/2, -x+1/2'
'z, -y+1/2, -x+1/2'
'z, y+1/2, x+1/2'
'-x+1/2, -y, -z+1/2'
'x+1/2, y, -z+1/2'
'x+1/2, -y, z+1/2'
'-x+1/2, y, z+1/2'
'-z+1/2, -x, -y+1/2'
'-z+1/2, x, y+1/2'
'z+1/2, x, -y+1/2'
'z+1/2, -x, y+1/2'
'-y+1/2, -z, -x+1/2'
'y+1/2, -z, x+1/2'
'-y+1/2, z, x+1/2'
'y+1/2, z, -x+1/2'
'-y+1/2, -x, z+1/2'
```

```
 'y+1/2, x, z+1/2'
 '-y+1/2, x, -z+1/2'
 'y+1/2, -x, -z+1/2'
 '-x+1/2, -z, y+1/2'
 'x+1/2, -z, -y+1/2'
 'x+1/2, z, y+1/2'
 '-x+1/2, z, -y+1/2'
 '-z+1/2, -y, x+1/2'
 '-z+1/2, y, -x+1/2'
 'z+1/2, -y, -x+1/2'
 'z+1/2, y, x+1/2'
 '-x+1/2, -y+1/2, -z'
 'x+1/2, y+1/2, -z'
 'x+1/2, -y+1/2, z'
 '-x+1/2, y+1/2, z'
 '-z+1/2, -x+1/2, -y'
 '-z+1/2, x+1/2, y'
 'z+1/2, x+1/2, -y'
 'z+1/2, -x+1/2, y'
 '-y+1/2, -z+1/2, -x'
 'y+1/2, -z+1/2, x'
 '-y+1/2, z+1/2, x'
 'y+1/2, z+1/2, -x'
 '-y+1/2, -x+1/2, z'
 'y+1/2, x+1/2, z'
 '-y+1/2, x+1/2, -z'
 'y+1/2, -x+1/2, -z'
 '-x+1/2, -z+1/2, y'
 'x+1/2, -z+1/2, -y'
 'x+1/2, z+1/2, y'
 '-x+1/2, z+1/2, -y'
 '-z+1/2, -y+1/2, x'
 '-z+1/2, y+1/2, -x'
 'z+1/2, -y+1/2, -x'
 'z+1/2, y+1/2, x'

_cell_length_a                    7.9686(6)
_cell_length_b                    7.9686(6)
_cell_length_c                    7.9686(6)
_cell_angle_alpha                 90
_cell_angle_beta                  90
_cell_angle_gamma                 90
_cell_volume                      505.99(11)
_cell_formula_units_Z             8
_cell_measurement_temperature     400(2)
_cell_measurement_reflns_used     2514
_cell_measurement_theta_min       2.973
_cell_measurement_theta_max       23.473

_exptl_crystal_description        prizm
_exptl_crystal_colour             dark
_exptl_crystal_density_meas       ?
_exptl_crystal_density_method     ?
_exptl_crystal_density_diffrn     9.275
```

```
_exptl_crystal_F_000              1183
_exptl_transmission_factor_min    ?
_exptl_transmission_factor_max    ?
_exptl_crystal_size_max           0.070
_exptl_crystal_size_mid           0.020
_exptl_crystal_size_min           0.010
_exptl_absorpt_coefficient_mu     20.757
_shelx_estimated_absorpt_T_min    0.324
_shelx_estimated_absorpt_T_max    0.819
_exptl_absorpt_correction_type    multi-scan
_exptl_absorpt_correction_T_min   0.269
_exptl_absorpt_correction_T_max   0.428
_exptl_absorpt_process_details
;
 SADABS-2014/4 - Bruker AXS area detector scaling
 and absorption correction
;
_diffrn_ambient_temperature       400(2)
_diffrn_radiation_wavelength      0.41328
_diffrn_radiation_type            'synchrotron'
_diffrn_source                    'Advanced photon source'
_diffrn_radiation_monochromator   'diamond (111)'
_diffrn_measurement_device_type
;
Huber 3 circle diffractometer with PILATUS3 X CdTe 1M detector
;
_diffrn_measurement_method        '\f-scan'
_diffrn_detector_area_resol_mean  ?
_diffrn_standards_number          0
_diffrn_standards_interval_count  0
_diffrn_standards_interval_time   0
_diffrn_standards_decay_%         0.0
_diffrn_reflns_number             2500
_diffrn_reflns_av_unetI/netI      0.0234
_diffrn_reflns_av_R_equivalents   0.0599
_diffrn_reflns_limit_h_min        -13
_diffrn_reflns_limit_h_max        13
_diffrn_reflns_limit_k_min        -14
_diffrn_reflns_limit_k_max        14
_diffrn_reflns_limit_l_min        -12
_diffrn_reflns_limit_l_max        10
_diffrn_reflns_theta_min          2.574
_diffrn_reflns_theta_max          24.103
_diffrn_reflns_theta_full         14.357
_diffrn_measured_fraction_theta_max   0.928
_diffrn_measured_fraction_theta_full  0.950
_diffrn_reflns_Laue_measured_fraction_max    0.928
_diffrn_reflns_Laue_measured_fraction_full   0.950
_diffrn_reflns_point_group_measured_fraction_max   0.928
_diffrn_reflns_point_group_measured_fraction_full  0.950
_reflns_number_total              129
_reflns_number_gt                 124
_reflns_threshold_expression      'I > 2\s(I)'
_reflns_Friedel_coverage          0.000
```

```
_reflns_Friedel_fraction_max      .
_reflns_Friedel_fraction_full     .

_reflns_special_details
;
 Reflections were merged by SHELXL according to the crystal
 class for the calculation of statistics and refinement.

 _reflns_Friedel_fraction is defined as the number of unique
 Friedel pairs measured divided by the number that would be
 possible theoretically, ignoring centric projections and
 systematic absences.
;

_computing_cell_refinement        'SAINT V8.32B (Bruker AXS Inc.,
2013)'
_computing_data_collection        ?
_computing_data_reduction         'SAINT V8.32B (Bruker AXS Inc.,
2013)'
_computing_molecular_graphics     'Bruker SHELXTL (Bruker AXS
Inc., 2013)'
_computing_structure_refinement   'SHELXL-2014/7 (Sheldrick,
2014)'
_computing_structure_solution     'SHELXT-2014/4 (Sheldrick,
2014)'
_computing_publication_material   ?
_refine_special_details           ?
_refine_ls_structure_factor_coef  Fsqd
_refine_ls_matrix_type            full
_refine_ls_weighting_scheme       calc
_refine_ls_weighting_details
'w=1/[\s^2^(Fo^2^)+(0.0182P)^2^+2.5921P] where P=(Fo^2^+2Fc^2^)/3'
_atom_sites_solution_primary      ?
_atom_sites_solution_secondary    ?
_atom_sites_solution_hydrogens    .
_refine_ls_hydrogen_treatment     undef
_refine_ls_extinction_method      'SHELXL-2014/7 (Sheldrick 2014'
_refine_ls_extinction_coef        0.57(4)
_refine_ls_extinction_expression
 'Fc^*^=kFc[1+0.001xFc^2^\l^3^/sin(2\q)]^-1/4^'
_refine_ls_number_reflns          129
_refine_ls_number_parameters      11
_refine_ls_number_restraints      0
_refine_ls_R_factor_all           0.0198
_refine_ls_R_factor_gt            0.0197
_refine_ls_wR_factor_ref          0.0617
_refine_ls_wR_factor_gt           0.0617
_refine_ls_goodness_of_fit_ref    1.363
_refine_ls_restrained_S_all       1.363
_refine_ls_shift/su_max           0.000
_refine_ls_shift/su_mean          0.000

loop_
 _atom_site_label
```

```
 _atom_site_type_symbol
 _atom_site_fract_x
 _atom_site_fract_y
 _atom_site_fract_z
 _atom_site_U_iso_or_equiv
 _atom_site_adp_type
 _atom_site_occupancy
 _atom_site_site_symmetry_order
 _atom_site_calc_flag
 _atom_site_refinement_flags_posn
 _atom_site_refinement_flags_adp
 _atom_site_refinement_flags_occupancy
 _atom_site_disorder_assembly
 _atom_site_disorder_group
Pb1 Pb 0.22074(19) 0.2500 0.2500 0.0217(5) Uani 0.1667 4 d S T P .
.
W1 W 0.0000 0.0000 0.0000 0.0083(3) Uani 0.3081 48 d S T P . .
Fe1 Fe 0.0000 0.0000 0.0000 0.0083(3) Uani 0.6916 48 d S T P . .
W2 W 0.5000 0.5000 0.5000 0.0079(3) Uani 0.3581 48 d S T P . .
Fe2 Fe 0.5000 0.5000 0.5000 0.0079(3) Uani 0.6418 48 d S T P . .
O1 O 0.2503(2) 0.0000 0.0000 0.0172(6) Uani 1 8 d S T P . .

loop_
 _atom_site_aniso_label
 _atom_site_aniso_U_11
 _atom_site_aniso_U_22
 _atom_site_aniso_U_33
 _atom_site_aniso_U_23
 _atom_site_aniso_U_13
 _atom_site_aniso_U_12
Pb1 0.0146(6) 0.0253(5) 0.0253(5) -0.00084(10) 0.000 0.000
W1 0.0083(3) 0.0083(3) 0.0083(3) 0.000 0.000 0.000
Fe1 0.0083(3) 0.0083(3) 0.0083(3) 0.000 0.000 0.000
W2 0.0079(3) 0.0079(3) 0.0079(3) 0.000 0.000 0.000
Fe2 0.0079(3) 0.0079(3) 0.0079(3) 0.000 0.000 0.000
O1 0.0084(12) 0.0215(9) 0.0215(9) 0.000 0.000 0.000

_geom_special_details
;
 All esds (except the esd in the dihedral angle between two l.s.
planes)
 are estimated using the full covariance matrix.  The cell esds
are taken
 into account individually in the estimation of esds in distances,
angles
 and torsion angles; correlations between esds in cell parameters
are only
 used when they are defined by crystal symmetry.  An approximate
(isotropic)
 treatment of cell esds is used for estimating esds involving l.s.
planes.
;

loop_
```

```
 _geom_bond_atom_site_label_1
 _geom_bond_atom_site_label_2
 _geom_bond_distance
 _geom_bond_site_symmetry_2
 _geom_bond_publ_flag
Pb1 Pb1 0.330(2) 58 ?
Pb1 Pb1 0.330(2) 30 ?
Pb1 Pb1 0.330(2) 9 ?
Pb1 Pb1 0.330(2) 5 ?
Pb1 Pb1 0.466(3) 74 ?
Pb1 O1 2.6576(10) 9 ?
Pb1 O1 2.6576(10) 5 ?
Pb1 O1 2.6576(10) 129 ?
Pb1 O1 2.6576(10) 125 ?
Pb1 W1 3.3214(8) . ?
Pb1 Fe1 3.3214(8) . ?
Pb1 Fe1 3.3214(8) 25 ?
W1 O1 1.9942(18) 105 ?
W1 O1 1.9942(18) 9 ?
W1 O1 1.9942(18) 101 ?
W1 O1 1.9942(18) 5 ?
W1 O1 1.9942(18) 97 ?
W1 O1 1.9942(18) . ?
W1 Pb1 3.3214(8) 97 ?
W1 Pb1 3.3214(8) 105 ?
W1 Pb1 3.3214(8) 9 ?
W1 Pb1 3.3214(8) 5 ?
W1 Pb1 3.3214(8) 101 ?
Fe1 O1 1.9942(18) 105 ?
Fe1 O1 1.9942(18) 9 ?
Fe1 O1 1.9942(18) 101 ?
Fe1 O1 1.9942(18) 5 ?
Fe1 O1 1.9942(18) 97 ?
Fe1 O1 1.9942(18) . ?
Fe1 Pb1 3.3214(8) 97 ?
Fe1 Pb1 3.3214(8) 105 ?
Fe1 Pb1 3.3214(8) 9 ?
Fe1 Pb1 3.3214(8) 5 ?
Fe1 Pb1 3.3214(8) 101 ?
W2 O1 1.9901(18) 177_556 ?
W2 O1 1.9901(18) 81 ?
W2 O1 1.9901(18) 149_565 ?
W2 O1 1.9901(18) 53 ?
W2 O1 1.9901(18) 121_655 ?
W2 O1 1.9901(18) 25 ?
W2 Pb1 3.3214(8) 74 ?
W2 Pb1 3.3214(8) 154_565 ?
W2 Pb1 3.3214(8) 170_556 ?
W2 Pb1 3.3214(8) 58 ?
W2 Pb1 3.3214(8) 126_655 ?
W2 Pb1 3.3214(8) 30 ?
Fe2 O1 1.9901(18) 177_556 ?
Fe2 O1 1.9901(18) 81 ?
Fe2 O1 1.9901(18) 149_565 ?
```

```
Fe2 O1 1.9901(18) 53 ?
Fe2 O1 1.9901(18) 121_655 ?
Fe2 O1 1.9901(18) 25 ?
Fe2 Pb1 3.3214(8) 74 ?
Fe2 Pb1 3.3214(8) 154_565 ?
Fe2 Pb1 3.3214(8) 170_556 ?
Fe2 Pb1 3.3214(8) 58 ?
Fe2 Pb1 3.3214(8) 126_655 ?
Fe2 Pb1 3.3214(8) 30 ?
O1 Fe2 1.9901(18) 25_544 ?
O1 W2 1.9901(18) 25_544 ?
O1 Pb1 2.6576(10) 5 ?
O1 Pb1 2.6576(10) 6 ?
O1 Pb1 2.6576(10) 82_545 ?
O1 Pb1 2.6576(10) 9 ?
O1 Pb1 2.6576(10) 150_554 ?
O1 Pb1 2.6576(10) 177 ?
O1 Pb1 2.6576(10) 106 ?
O1 Pb1 2.6576(10) 149 ?

loop_
 _geom_angle_atom_site_label_1
 _geom_angle_atom_site_label_2
 _geom_angle_atom_site_label_3
 _geom_angle
 _geom_angle_site_symmetry_1
 _geom_angle_site_symmetry_3
 _geom_angle_publ_flag
Pb1 Pb1 Pb1 59.998(1) 58 30 ?
Pb1 Pb1 Pb1 89.997(1) 58 9 ?
Pb1 Pb1 Pb1 59.998(1) 30 9 ?
Pb1 Pb1 Pb1 60.0 58 5 ?
Pb1 Pb1 Pb1 89.997(1) 30 5 ?
Pb1 Pb1 Pb1 60.0 9 5 ?
Pb1 Pb1 Pb1 45.0 58 74 ?
Pb1 Pb1 Pb1 45.0 30 74 ?
Pb1 Pb1 Pb1 45.0 9 74 ?
Pb1 Pb1 Pb1 45.0 5 74 ?
Pb1 Pb1 O1 117.87(4) 58 9 ?
Pb1 Pb1 O1 176.44(2) 30 9 ?
Pb1 Pb1 O1 117.94(4) 9 9 ?
Pb1 Pb1 O1 86.44(2) 5 9 ?
Pb1 Pb1 O1 131.44(2) 74 9 ?
Pb1 Pb1 O1 176.44(2) 58 5 ?
Pb1 Pb1 O1 117.87(3) 30 5 ?
Pb1 Pb1 O1 86.44(2) 9 5 ?
Pb1 Pb1 O1 117.94(3) 5 5 ?
Pb1 Pb1 O1 131.44(2) 74 5 ?
O1 Pb1 O1 64.09(7) 9 5 ?
Pb1 Pb1 O1 117.94(3) 58 129 ?
Pb1 Pb1 O1 86.44(2) 30 129 ?
Pb1 Pb1 O1 117.87(3) 9 129 ?
Pb1 Pb1 O1 176.44(2) 5 129 ?
Pb1 Pb1 O1 131.44(2) 74 129 ?
```

O1 Pb1 O1 97.11(5) 9 129 ?
O1 Pb1 O1 63.94(7) 5 129 ?
Pb1 Pb1 O1 86.44(2) 58 125 ?
Pb1 Pb1 O1 117.94(3) 30 125 ?
Pb1 Pb1 O1 176.44(2) 9 125 ?
Pb1 Pb1 O1 117.87(3) 5 125 ?
Pb1 Pb1 O1 131.44(2) 74 125 ?
O1 Pb1 O1 63.94(7) 9 125 ?
O1 Pb1 O1 97.11(5) 5 125 ?
O1 Pb1 O1 64.09(7) 129 125 ?
Pb1 Pb1 W1 142.997(12) 58 . ?
Pb1 Pb1 W1 142.997(12) 30 . ?
Pb1 Pb1 W1 87.156(19) 9 . ?
Pb1 Pb1 W1 87.156(19) 5 . ?
Pb1 Pb1 W1 121.98(2) 74 . ?
O1 Pb1 W1 36.90(4) 9 . ?
O1 Pb1 W1 36.90(4) 5 . ?
O1 Pb1 W1 95.66(4) 129 . ?
O1 Pb1 W1 95.66(4) 125 . ?
Pb1 Pb1 Fe1 142.997(12) 58 . ?
Pb1 Pb1 Fe1 142.997(12) 30 . ?
Pb1 Pb1 Fe1 87.156(19) 9 . ?
Pb1 Pb1 Fe1 87.156(19) 5 . ?
Pb1 Pb1 Fe1 121.98(2) 74 . ?
O1 Pb1 Fe1 36.90(4) 9 . ?
O1 Pb1 Fe1 36.90(4) 5 . ?
O1 Pb1 Fe1 95.66(4) 129 . ?
O1 Pb1 Fe1 95.66(4) 125 . ?
W1 Pb1 Fe1 0.0 . . ?
Pb1 Pb1 Fe1 87.156(19) 58 25 ?
Pb1 Pb1 Fe1 87.156(19) 30 25 ?
Pb1 Pb1 Fe1 142.997(12) 9 25 ?
Pb1 Pb1 Fe1 142.997(12) 5 25 ?
Pb1 Pb1 Fe1 121.98(2) 74 25 ?
O1 Pb1 Fe1 95.66(4) 9 25 ?
O1 Pb1 Fe1 95.66(4) 5 25 ?
O1 Pb1 Fe1 36.90(4) 129 25 ?
O1 Pb1 Fe1 36.90(4) 125 25 ?
W1 Pb1 Fe1 116.04(4) . 25 ?
Fe1 Pb1 Fe1 116.04(4) . 25 ?
O1 W1 O1 180.0 105 9 ?
O1 W1 O1 90.0 105 101 ?
O1 W1 O1 90.0 9 101 ?
O1 W1 O1 90.0 105 5 ?
O1 W1 O1 90.0 9 5 ?
O1 W1 O1 180.0 101 5 ?
O1 W1 O1 90.0 105 97 ?
O1 W1 O1 90.0 9 97 ?
O1 W1 O1 90.0 101 97 ?
O1 W1 O1 90.0 5 97 ?
O1 W1 O1 90.0 105 . ?
O1 W1 O1 90.0 9 . ?
O1 W1 O1 90.0 101 . ?
O1 W1 O1 90.0 5 . ?

O1 W1 O1 180.0 97 . ?
O1 W1 Pb1 126.856(10) 105 . ?
O1 W1 Pb1 53.144(10) 9 . ?
O1 W1 Pb1 126.856(10) 101 . ?
O1 W1 Pb1 53.144(10) 5 . ?
O1 W1 Pb1 121.98(2) 97 . ?
O1 W1 Pb1 58.02(2) . . ?
O1 W1 Pb1 53.144(10) 105 97 ?
O1 W1 Pb1 126.856(10) 9 97 ?
O1 W1 Pb1 53.144(10) 101 97 ?
O1 W1 Pb1 126.856(10) 5 97 ?
O1 W1 Pb1 58.02(2) 97 97 ?
O1 W1 Pb1 121.98(2) . 97 ?
Pb1 W1 Pb1 180.0 . 97 ?
O1 W1 Pb1 58.02(2) 105 105 ?
O1 W1 Pb1 121.98(2) 9 105 ?
O1 W1 Pb1 53.144(10) 101 105 ?
O1 W1 Pb1 126.856(10) 5 105 ?
O1 W1 Pb1 53.144(10) 97 105 ?
O1 W1 Pb1 126.856(10) . 105 ?
Pb1 W1 Pb1 174.31(4) . 105 ?
Pb1 W1 Pb1 5.69(4) 97 105 ?
O1 W1 Pb1 121.98(2) 105 9 ?
O1 W1 Pb1 58.02(2) 9 9 ?
O1 W1 Pb1 126.856(10) 101 9 ?
O1 W1 Pb1 53.144(10) 5 9 ?
O1 W1 Pb1 126.856(10) 97 9 ?
O1 W1 Pb1 53.144(10) . 9 ?
Pb1 W1 Pb1 5.69(4) . 9 ?
Pb1 W1 Pb1 174.31(4) 97 9 ?
Pb1 W1 Pb1 180.0 105 9 ?
O1 W1 Pb1 126.856(10) 105 5 ?
O1 W1 Pb1 53.144(10) 9 5 ?
O1 W1 Pb1 121.98(2) 101 5 ?
O1 W1 Pb1 58.02(2) 5 5 ?
O1 W1 Pb1 126.856(10) 97 5 ?
O1 W1 Pb1 53.144(10) . 5 ?
Pb1 W1 Pb1 5.69(4) . 5 ?
Pb1 W1 Pb1 174.31(4) 97 5 ?
Pb1 W1 Pb1 174.31(4) 105 5 ?
Pb1 W1 Pb1 5.69(4) 9 5 ?
O1 W1 Pb1 53.144(10) 105 101 ?
O1 W1 Pb1 126.856(10) 9 101 ?
O1 W1 Pb1 58.02(2) 101 101 ?
O1 W1 Pb1 121.98(2) 5 101 ?
O1 W1 Pb1 53.144(10) 97 101 ?
O1 W1 Pb1 126.856(10) . 101 ?
Pb1 W1 Pb1 174.31(4) . 101 ?
Pb1 W1 Pb1 5.69(4) 97 101 ?
Pb1 W1 Pb1 5.69(4) 105 101 ?
Pb1 W1 Pb1 174.31(4) 9 101 ?
Pb1 W1 Pb1 180.0 5 101 ?
O1 Fe1 O1 180.0 105 9 ?
O1 Fe1 O1 90.0 105 101 ?

O1 Fe1 O1 90.0 9 101 ?
O1 Fe1 O1 90.0 105 5 ?
O1 Fe1 O1 90.0 9 5 ?
O1 Fe1 O1 180.0 101 5 ?
O1 Fe1 O1 90.0 105 97 ?
O1 Fe1 O1 90.0 9 97 ?
O1 Fe1 O1 90.0 101 97 ?
O1 Fe1 O1 90.0 5 97 ?
O1 Fe1 O1 90.0 105 . ?
O1 Fe1 O1 90.0 9 . ?
O1 Fe1 O1 90.0 101 . ?
O1 Fe1 O1 90.0 5 . ?
O1 Fe1 O1 180.0 97 . ?
O1 Fe1 Pb1 126.856(10) 105 . ?
O1 Fe1 Pb1 53.144(10) 9 . ?
O1 Fe1 Pb1 126.856(10) 101 . ?
O1 Fe1 Pb1 53.144(10) 5 . ?
O1 Fe1 Pb1 121.98(2) 97 . ?
O1 Fe1 Pb1 58.02(2) . . ?
O1 Fe1 Pb1 53.144(10) 105 97 ?
O1 Fe1 Pb1 126.856(10) 9 97 ?
O1 Fe1 Pb1 53.144(10) 101 97 ?
O1 Fe1 Pb1 126.856(10) 5 97 ?
O1 Fe1 Pb1 58.02(2) 97 97 ?
O1 Fe1 Pb1 121.98(2) . 97 ?
Pb1 Fe1 Pb1 180.0 . 97 ?
O1 Fe1 Pb1 58.02(2) 105 105 ?
O1 Fe1 Pb1 121.98(2) 9 105 ?
O1 Fe1 Pb1 53.144(10) 101 105 ?
O1 Fe1 Pb1 126.856(10) 5 105 ?
O1 Fe1 Pb1 53.144(10) 97 105 ?
O1 Fe1 Pb1 126.856(10) . 105 ?
Pb1 Fe1 Pb1 174.31(4) . 105 ?
Pb1 Fe1 Pb1 5.69(4) 97 105 ?
O1 Fe1 Pb1 121.98(2) 105 9 ?
O1 Fe1 Pb1 58.02(2) 9 9 ?
O1 Fe1 Pb1 126.856(10) 101 9 ?
O1 Fe1 Pb1 53.144(10) 5 9 ?
O1 Fe1 Pb1 126.856(10) 97 9 ?
O1 Fe1 Pb1 53.144(10) . 9 ?
Pb1 Fe1 Pb1 5.69(4) . 9 ?
Pb1 Fe1 Pb1 174.31(4) 97 9 ?
Pb1 Fe1 Pb1 180.0 105 9 ?
O1 Fe1 Pb1 126.856(10) 105 5 ?
O1 Fe1 Pb1 53.144(10) 9 5 ?
O1 Fe1 Pb1 121.98(2) 101 5 ?
O1 Fe1 Pb1 58.02(2) 5 5 ?
O1 Fe1 Pb1 126.856(10) 97 5 ?
O1 Fe1 Pb1 53.144(10) . 5 ?
Pb1 Fe1 Pb1 5.69(4) . 5 ?
Pb1 Fe1 Pb1 174.31(4) 97 5 ?
Pb1 Fe1 Pb1 174.31(4) 105 5 ?
Pb1 Fe1 Pb1 5.69(4) 9 5 ?
O1 Fe1 Pb1 53.144(10) 105 101 ?

O1 Fe1 Pb1 126.856(10) 9 101 ?
O1 Fe1 Pb1 58.02(2) 101 101 ?
O1 Fe1 Pb1 121.98(2) 5 101 ?
O1 Fe1 Pb1 53.144(10) 97 101 ?
O1 Fe1 Pb1 126.856(10) . 101 ?
Pb1 Fe1 Pb1 174.31(4) . 101 ?
Pb1 Fe1 Pb1 5.69(4) 97 101 ?
Pb1 Fe1 Pb1 5.69(4) 105 101 ?
Pb1 Fe1 Pb1 174.31(4) 9 101 ?
Pb1 Fe1 Pb1 180.0 5 101 ?
O1 W2 O1 180.0 177_556 81 ?
O1 W2 O1 90.0 177_556 149_565 ?
O1 W2 O1 90.0 81 149_565 ?
O1 W2 O1 90.0 177_556 53 ?
O1 W2 O1 90.0 81 53 ?
O1 W2 O1 180.0 149_565 53 ?
O1 W2 O1 90.0 177_556 121_655 ?
O1 W2 O1 90.0 81 121_655 ?
O1 W2 O1 90.0 149_565 121_655 ?
O1 W2 O1 90.0 53 121_655 ?
O1 W2 O1 90.0 177_556 25 ?
O1 W2 O1 90.0 81 25 ?
O1 W2 O1 90.0 149_565 25 ?
O1 W2 O1 90.0 53 25 ?
O1 W2 O1 180.0 121_655 25 ?
O1 W2 Pb1 126.856(11) 177_556 74 ?
O1 W2 Pb1 53.144(10) 81 74 ?
O1 W2 Pb1 126.856(10) 149_565 74 ?
O1 W2 Pb1 53.144(10) 53 74 ?
O1 W2 Pb1 121.98(2) 121_655 74 ?
O1 W2 Pb1 58.02(2) 25 74 ?
O1 W2 Pb1 58.02(2) 177_556 154_565 ?
O1 W2 Pb1 121.98(2) 81 154_565 ?
O1 W2 Pb1 53.144(10) 149_565 154_565 ?
O1 W2 Pb1 126.856(10) 53 154_565 ?
O1 W2 Pb1 53.144(10) 121_655 154_565 ?
O1 W2 Pb1 126.856(10) 25 154_565 ?
Pb1 W2 Pb1 174.31(4) 74 154_565 ?
O1 W2 Pb1 53.144(10) 177_556 170_556 ?
O1 W2 Pb1 126.856(11) 81 170_556 ?
O1 W2 Pb1 53.144(10) 149_565 170_556 ?
O1 W2 Pb1 126.856(10) 53 170_556 ?
O1 W2 Pb1 58.02(2) 121_655 170_556 ?
O1 W2 Pb1 121.98(2) 25 170_556 ?
Pb1 W2 Pb1 180.0 74 170_556 ?
Pb1 W2 Pb1 5.69(4) 154_565 170_556 ?
O1 W2 Pb1 121.98(2) 177_556 58 ?
O1 W2 Pb1 58.02(2) 81 58 ?
O1 W2 Pb1 126.856(10) 149_565 58 ?
O1 W2 Pb1 53.144(10) 53 58 ?
O1 W2 Pb1 126.856(10) 121_655 58 ?
O1 W2 Pb1 53.144(10) 25 58 ?
Pb1 W2 Pb1 5.69(4) 74 58 ?
Pb1 W2 Pb1 180.0 154_565 58 ?

Pb1 W2 Pb1 174.31(4) 170_556 58 ?
O1 W2 Pb1 53.144(10) 177_556 126_655 ?
O1 W2 Pb1 126.856(11) 81 126_655 ?
O1 W2 Pb1 58.02(2) 149_565 126_655 ?
O1 W2 Pb1 121.98(2) 53 126_655 ?
O1 W2 Pb1 53.144(10) 121_655 126_655 ?
O1 W2 Pb1 126.856(10) 25 126_655 ?
Pb1 W2 Pb1 174.31(4) 74 126_655 ?
Pb1 W2 Pb1 5.69(4) 154_565 126_655 ?
Pb1 W2 Pb1 5.69(4) 170_556 126_655 ?
Pb1 W2 Pb1 174.31(4) 58 126_655 ?
O1 W2 Pb1 126.856(11) 177_556 30 ?
O1 W2 Pb1 53.144(10) 81 30 ?
O1 W2 Pb1 121.98(2) 149_565 30 ?
O1 W2 Pb1 58.02(2) 53 30 ?
O1 W2 Pb1 126.856(10) 121_655 30 ?
O1 W2 Pb1 53.144(10) 25 30 ?
Pb1 W2 Pb1 5.69(4) 74 30 ?
Pb1 W2 Pb1 174.31(4) 154_565 30 ?
Pb1 W2 Pb1 174.31(4) 170_556 30 ?
Pb1 W2 Pb1 5.69(4) 58 30 ?
Pb1 W2 Pb1 180.0 126_655 30 ?
O1 Fe2 O1 180.0 177_556 81 ?
O1 Fe2 O1 90.0 177_556 149_565 ?
O1 Fe2 O1 90.0 81 149_565 ?
O1 Fe2 O1 90.0 177_556 53 ?
O1 Fe2 O1 90.0 81 53 ?
O1 Fe2 O1 180.0 149_565 53 ?
O1 Fe2 O1 90.0 177_556 121_655 ?
O1 Fe2 O1 90.0 81 121_655 ?
O1 Fe2 O1 90.0 149_565 121_655 ?
O1 Fe2 O1 90.0 53 121_655 ?
O1 Fe2 O1 90.0 177_556 25 ?
O1 Fe2 O1 90.0 81 25 ?
O1 Fe2 O1 90.0 149_565 25 ?
O1 Fe2 O1 90.0 53 25 ?
O1 Fe2 O1 180.0 121_655 25 ?
O1 Fe2 Pb1 126.856(11) 177_556 74 ?
O1 Fe2 Pb1 53.144(10) 81 74 ?
O1 Fe2 Pb1 126.856(10) 149_565 74 ?
O1 Fe2 Pb1 53.144(10) 53 74 ?
O1 Fe2 Pb1 121.98(2) 121_655 74 ?
O1 Fe2 Pb1 58.02(2) 25 74 ?
O1 Fe2 Pb1 58.02(2) 177_556 154_565 ?
O1 Fe2 Pb1 121.98(2) 81 154_565 ?
O1 Fe2 Pb1 53.144(10) 149_565 154_565 ?
O1 Fe2 Pb1 126.856(10) 53 154_565 ?
O1 Fe2 Pb1 53.144(10) 121_655 154_565 ?
O1 Fe2 Pb1 126.856(10) 25 154_565 ?
Pb1 Fe2 Pb1 174.31(4) 74 154_565 ?
O1 Fe2 Pb1 53.144(10) 177_556 170_556 ?
O1 Fe2 Pb1 126.856(11) 81 170_556 ?
O1 Fe2 Pb1 53.144(10) 149_565 170_556 ?
O1 Fe2 Pb1 126.856(10) 53 170_556 ?

O1 Fe2 Pb1 58.02(2) 121_655 170_556 ?
O1 Fe2 Pb1 121.98(2) 25 170_556 ?
Pb1 Fe2 Pb1 180.0 74 170_556 ?
Pb1 Fe2 Pb1 5.69(4) 154_565 170_556 ?
O1 Fe2 Pb1 121.98(2) 177_556 58 ?
O1 Fe2 Pb1 58.02(2) 81 58 ?
O1 Fe2 Pb1 126.856(10) 149_565 58 ?
O1 Fe2 Pb1 53.144(10) 53 58 ?
O1 Fe2 Pb1 126.856(10) 121_655 58 ?
O1 Fe2 Pb1 53.144(10) 25 58 ?
Pb1 Fe2 Pb1 5.69(4) 74 58 ?
Pb1 Fe2 Pb1 180.0 154_565 58 ?
Pb1 Fe2 Pb1 174.31(4) 170_556 58 ?
O1 Fe2 Pb1 53.144(10) 177_556 126_655 ?
O1 Fe2 Pb1 126.856(11) 81 126_655 ?
O1 Fe2 Pb1 58.02(2) 149_565 126_655 ?
O1 Fe2 Pb1 121.98(2) 53 126_655 ?
O1 Fe2 Pb1 53.144(10) 121_655 126_655 ?
O1 Fe2 Pb1 126.856(10) 25 126_655 ?
Pb1 Fe2 Pb1 174.31(4) 74 126_655 ?
Pb1 Fe2 Pb1 5.69(4) 154_565 126_655 ?
Pb1 Fe2 Pb1 5.69(4) 170_556 126_655 ?
Pb1 Fe2 Pb1 174.31(4) 58 126_655 ?
O1 Fe2 Pb1 126.856(11) 177_556 30 ?
O1 Fe2 Pb1 53.144(10) 81 30 ?
O1 Fe2 Pb1 121.98(2) 149_565 30 ?
O1 Fe2 Pb1 58.02(2) 53 30 ?
O1 Fe2 Pb1 126.856(10) 121_655 30 ?
O1 Fe2 Pb1 53.144(10) 25 30 ?
Pb1 Fe2 Pb1 5.69(4) 74 30 ?
Pb1 Fe2 Pb1 174.31(4) 154_565 30 ?
Pb1 Fe2 Pb1 174.31(4) 170_556 30 ?
Pb1 Fe2 Pb1 5.69(4) 58 30 ?
Pb1 Fe2 Pb1 180.0 126_655 30 ?
Fe2 O1 W2 0.0 25_544 25_544 ?
Fe2 O1 W1 180.0 25_544 . ?
W2 O1 W1 180.0 25_544 . ?
Fe2 O1 Fe1 180.0 25_544 . ?
W2 O1 Fe1 180.0 25_544 . ?
W1 O1 Fe1 0.0 . . ?
Fe2 O1 Pb1 90.04(4) 25_544 5 ?
W2 O1 Pb1 90.04(4) 25_544 5 ?
W1 O1 Pb1 89.96(4) . 5 ?
Fe1 O1 Pb1 89.96(4) . 5 ?
Fe2 O1 Pb1 90.04(4) 25_544 6 ?
W2 O1 Pb1 90.04(4) 25_544 6 ?
W1 O1 Pb1 89.96(4) . 6 ?
Fe1 O1 Pb1 89.96(4) . 6 ?
Pb1 O1 Pb1 179.91(8) 5 6 ?
Fe2 O1 Pb1 90.04(4) 25_544 82_545 ?
W2 O1 Pb1 90.04(4) 25_544 82_545 ?
W1 O1 Pb1 89.96(4) . 82_545 ?
Fe1 O1 Pb1 89.96(4) . 82_545 ?
Pb1 O1 Pb1 172.89(5) 5 82_545 ?

```
Pb1 O1 Pb1 7.11(5) 6 82_545 ?
Fe2 O1 Pb1 90.04(4) 25_544 9 ?
W2 O1 Pb1 90.04(4) 25_544 9 ?
W1 O1 Pb1 89.96(4) . 9 ?
Fe1 O1 Pb1 89.96(4) . 9 ?
Pb1 O1 Pb1 7.11(5) 5 9 ?
Pb1 O1 Pb1 172.89(5) 6 9 ?
Pb1 O1 Pb1 179.91(8) 82_545 9 ?
Fe2 O1 Pb1 90.04(4) 25_544 150_554 ?
W2 O1 Pb1 90.04(4) 25_544 150_554 ?
W1 O1 Pb1 89.96(4) . 150_554 ?
Fe1 O1 Pb1 89.96(4) . 150_554 ?
Pb1 O1 Pb1 97.11(5) 5 150_554 ?
Pb1 O1 Pb1 82.89(5) 6 150_554 ?
Pb1 O1 Pb1 90.0 82_545 150_554 ?
Pb1 O1 Pb1 90.0 9 150_554 ?
Fe2 O1 Pb1 90.04(4) 25_544 177 ?
W2 O1 Pb1 90.04(4) 25_544 177 ?
W1 O1 Pb1 89.96(4) . 177 ?
Fe1 O1 Pb1 89.96(4) . 177 ?
Pb1 O1 Pb1 90.0 5 177 ?
Pb1 O1 Pb1 90.0 6 177 ?
Pb1 O1 Pb1 97.11(5) 82_545 177 ?
Pb1 O1 Pb1 82.89(5) 9 177 ?
Pb1 O1 Pb1 7.11(5) 150_554 177 ?
Fe2 O1 Pb1 90.04(4) 25_544 106 ?
W2 O1 Pb1 90.04(4) 25_544 106 ?
W1 O1 Pb1 89.96(4) . 106 ?
Fe1 O1 Pb1 89.96(4) . 106 ?
Pb1 O1 Pb1 90.0 5 106 ?
Pb1 O1 Pb1 90.0 6 106 ?
Pb1 O1 Pb1 82.89(5) 82_545 106 ?
Pb1 O1 Pb1 97.11(5) 9 106 ?
Pb1 O1 Pb1 172.89(5) 150_554 106 ?
Pb1 O1 Pb1 179.91(8) 177 106 ?
Fe2 O1 Pb1 90.04(4) 25_544 149 ?
W2 O1 Pb1 90.04(4) 25_544 149 ?
W1 O1 Pb1 89.96(4) . 149 ?
Fe1 O1 Pb1 89.96(4) . 149 ?
Pb1 O1 Pb1 82.89(5) 5 149 ?
Pb1 O1 Pb1 97.11(5) 6 149 ?
Pb1 O1 Pb1 90.0 82_545 149 ?
Pb1 O1 Pb1 90.0 9 149 ?
Pb1 O1 Pb1 179.91(8) 150_554 149 ?
Pb1 O1 Pb1 172.89(5) 177 149 ?
Pb1 O1 Pb1 7.11(5) 106 149 ?

_refine_diff_density_max    1.050
_refine_diff_density_min   -1.098
_refine_diff_density_rms    0.218
```
